# Supplementary figures and images for: A carnivorous plant genetic map: pitcher/insect-capture QTL on a genetic linkage map of Sarracenia
Source: Life Sci Alliance. 2018 Nov 29;1(6):e201800146. doi: 10.26508/lsa.201800146 (PMC6265660; doi:10.26508/lsa.201800146)

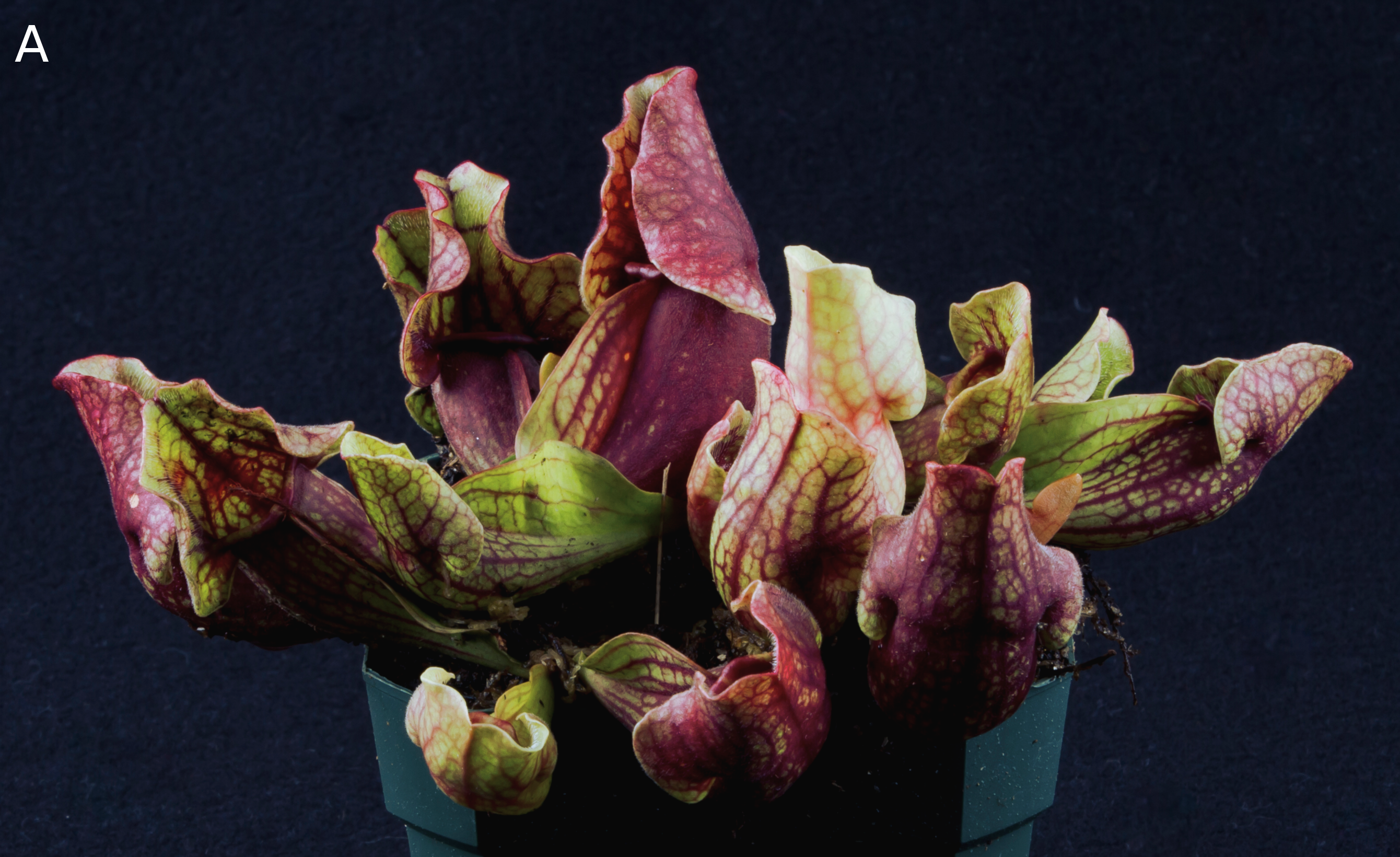

Supplement: Supplementary file 3 [file LSA-2018-00146_SdataF3.zip › Fig3A.tif]

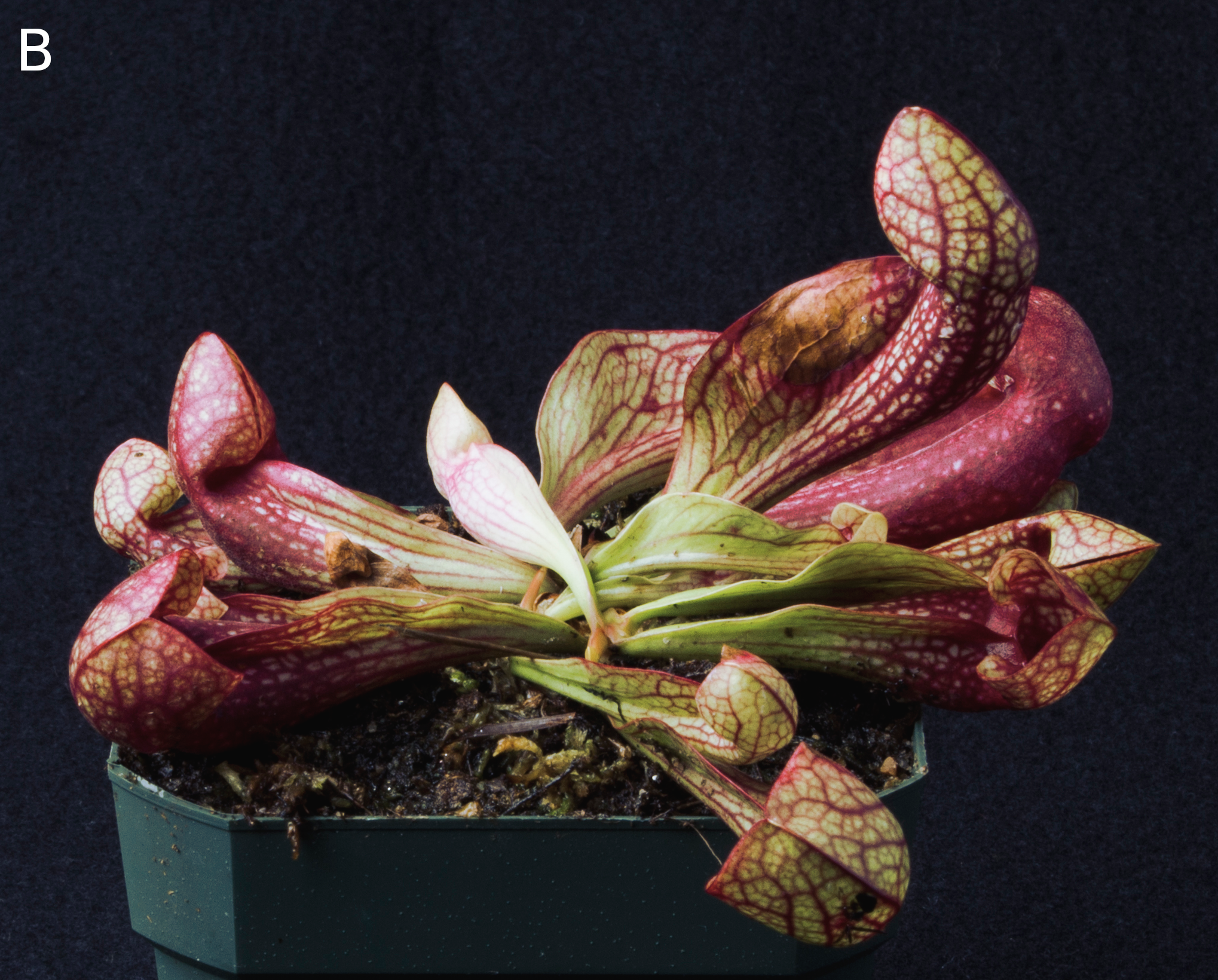

Supplement: Supplementary file 3 [file LSA-2018-00146_SdataF3.zip › Fig3B.tif]

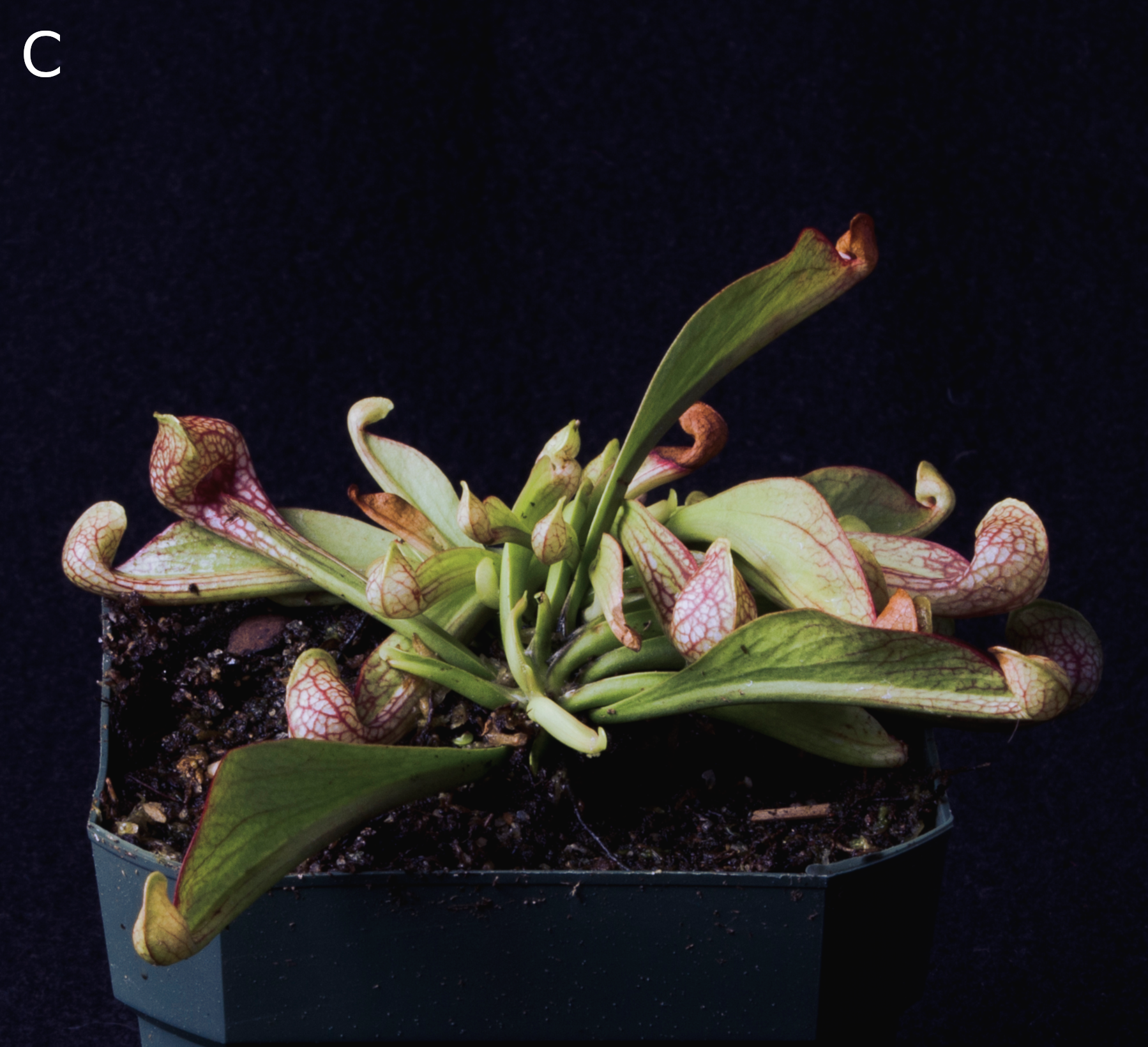

Supplement: Supplementary file 3 [file LSA-2018-00146_SdataF3.zip › Fig3C.tif]

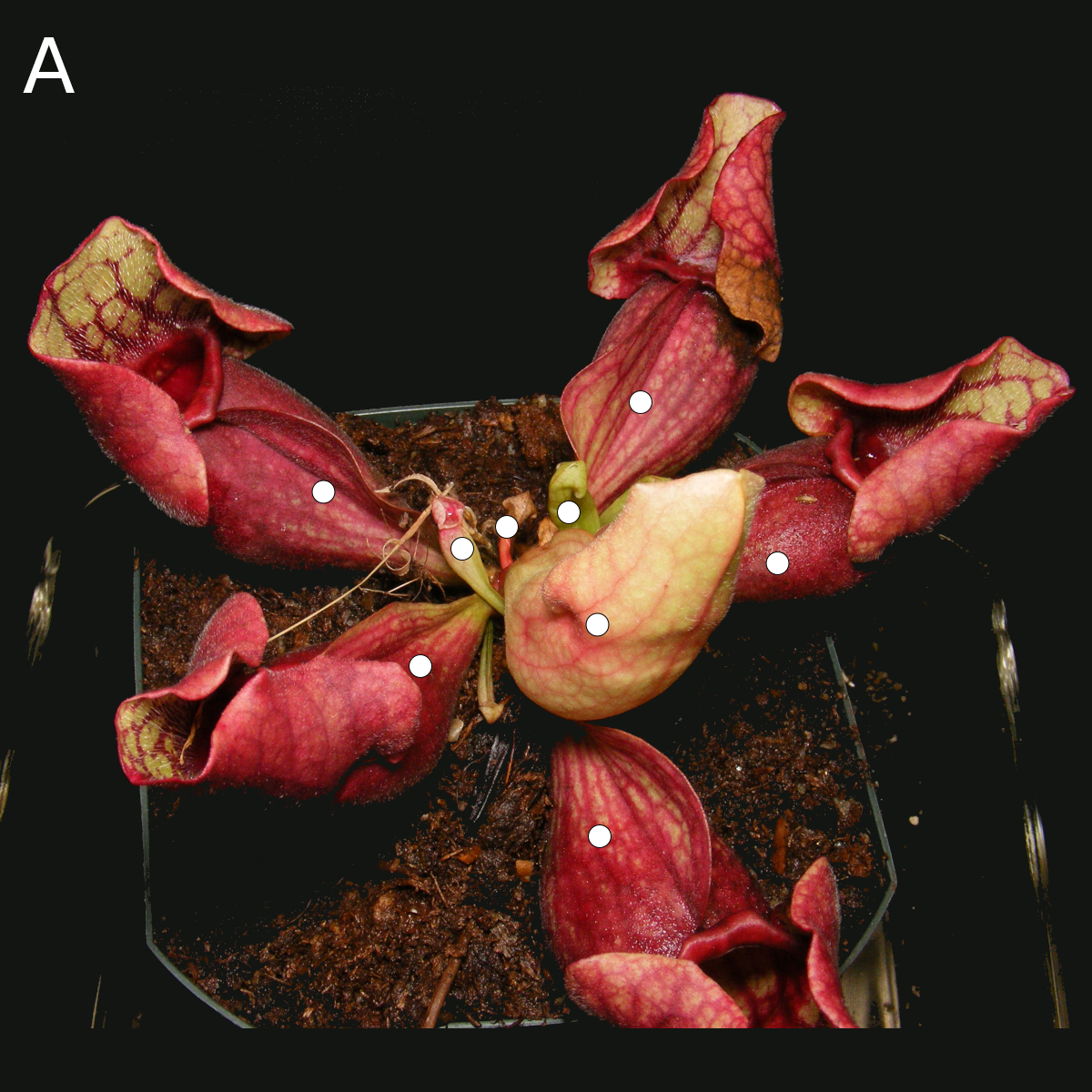

Supplement: Supplementary file 4 [file LSA-2018-00146_SdataF4.zip › Fig4A.tif]

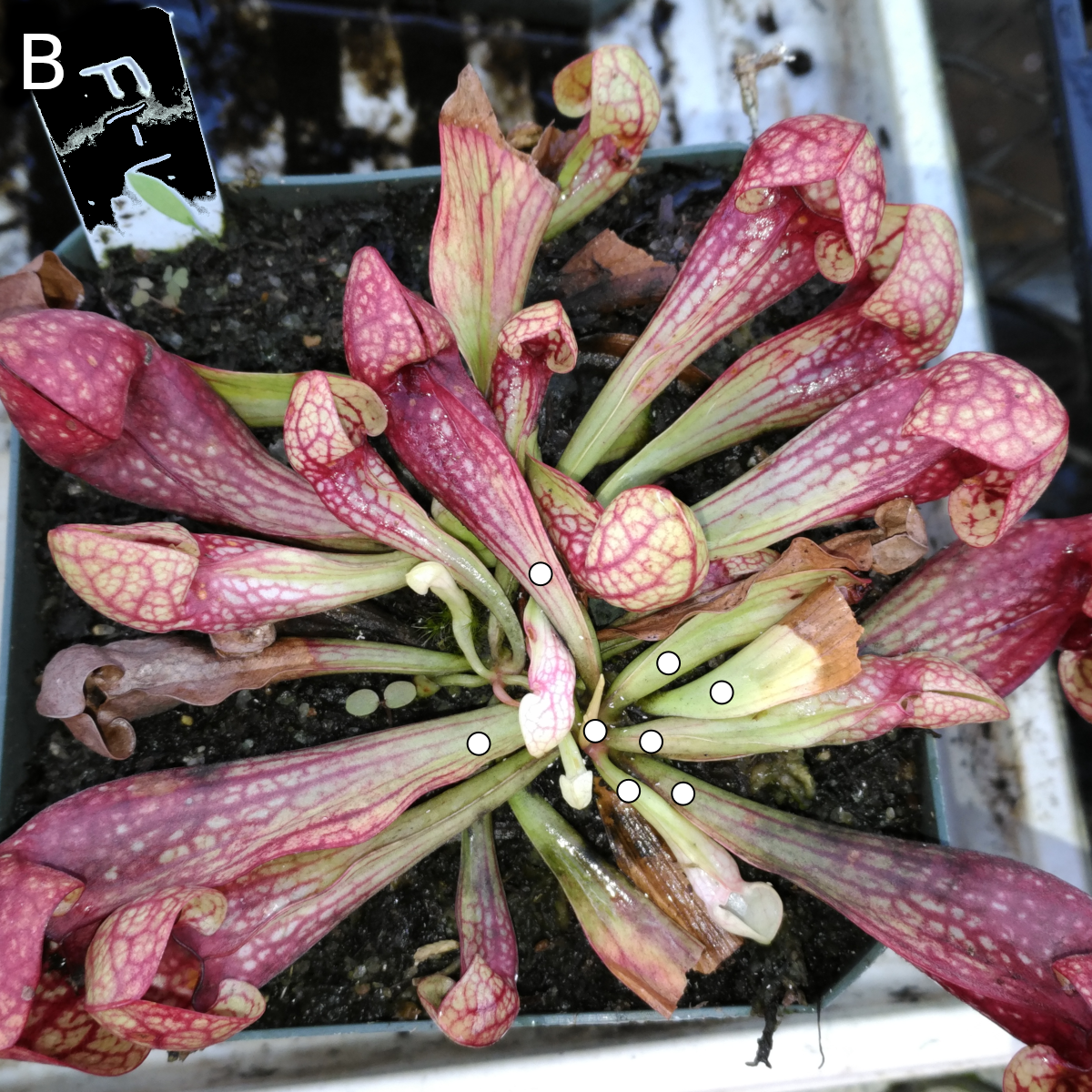

Supplement: Supplementary file 4 [file LSA-2018-00146_SdataF4.zip › Fig4B.tif]

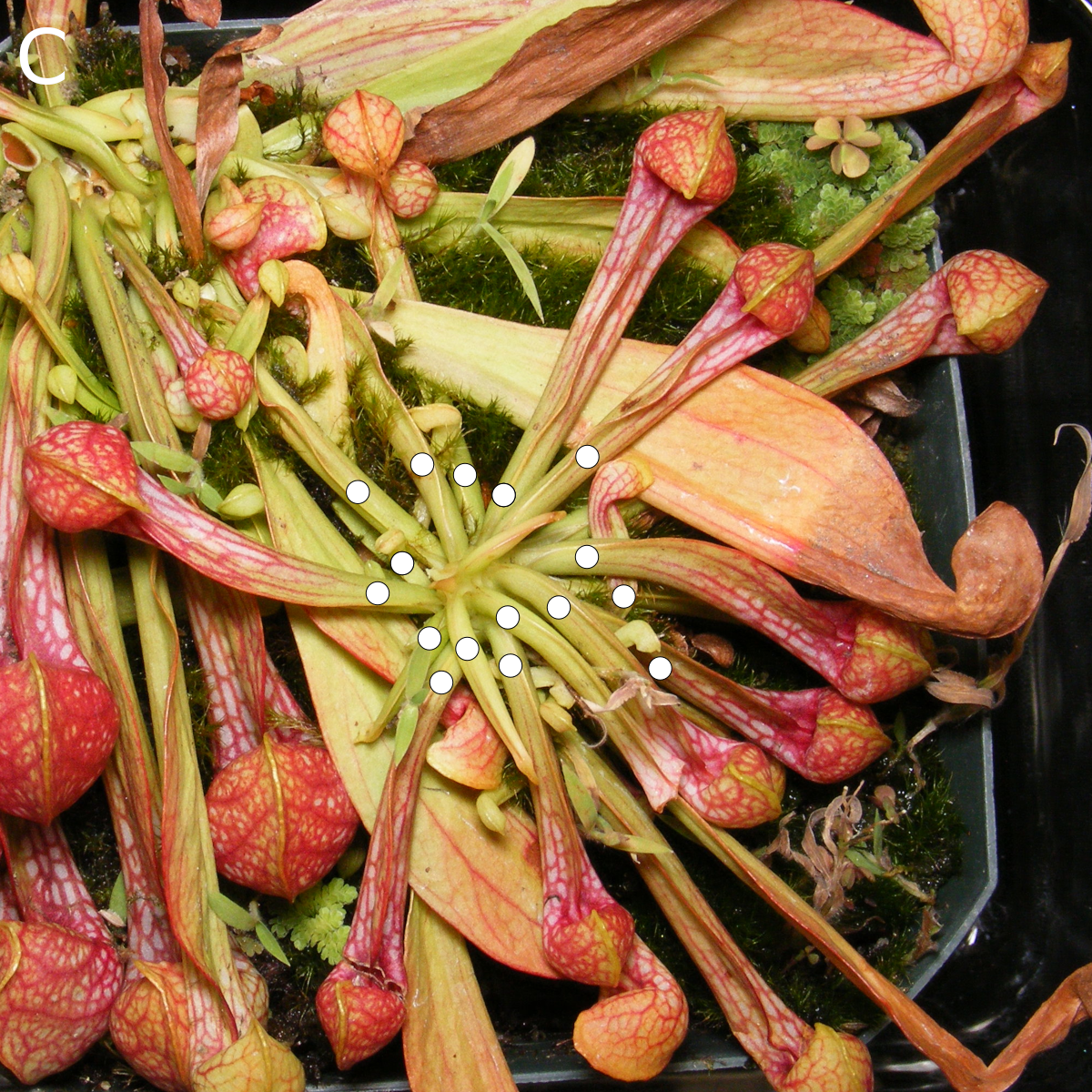

Supplement: Supplementary file 4 [file LSA-2018-00146_SdataF4.zip › Fig4C.tif]

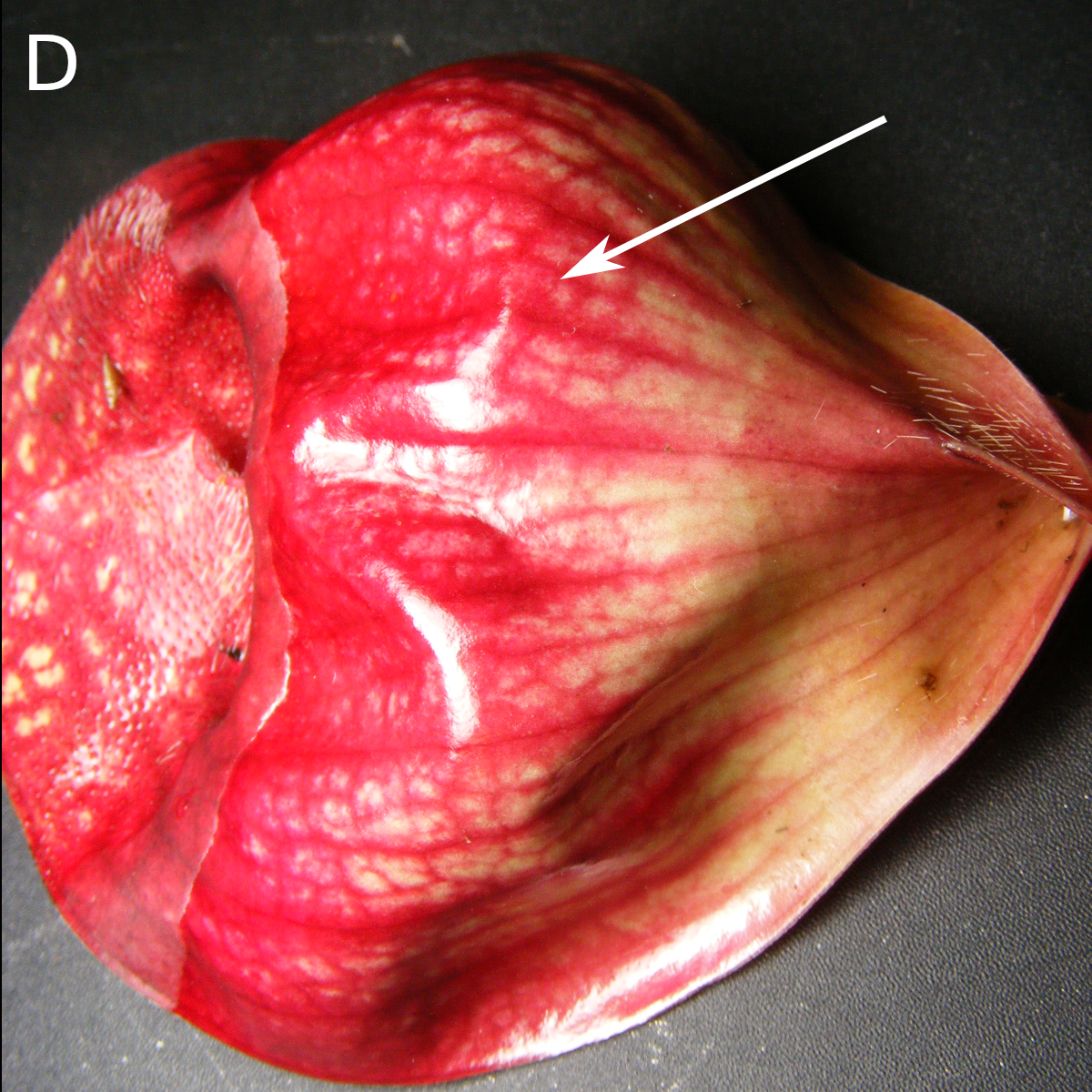

Supplement: Supplementary file 4 [file LSA-2018-00146_SdataF4.zip › Fig4D.tif]

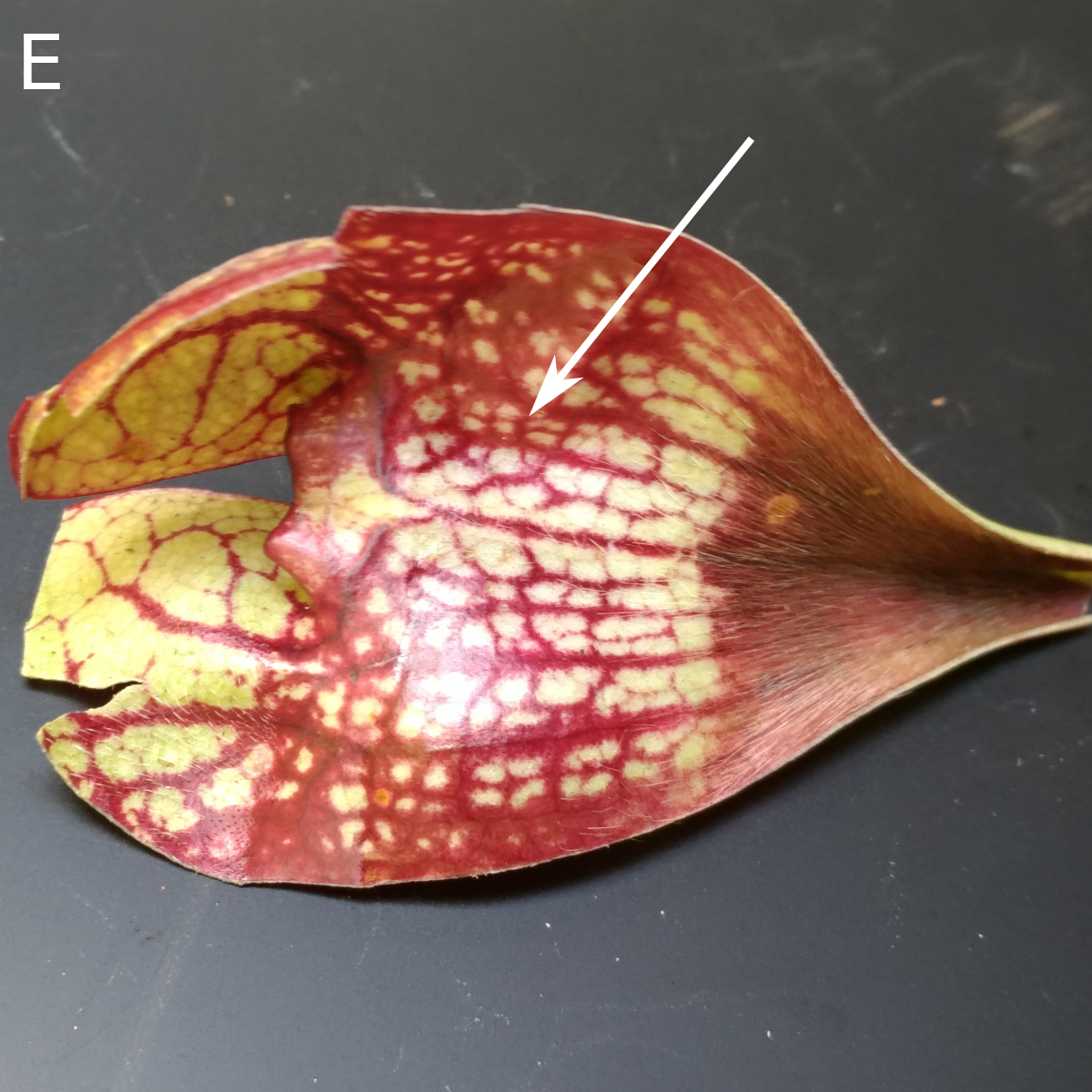

Supplement: Supplementary file 4 [file LSA-2018-00146_SdataF4.zip › Fig4E.tif]

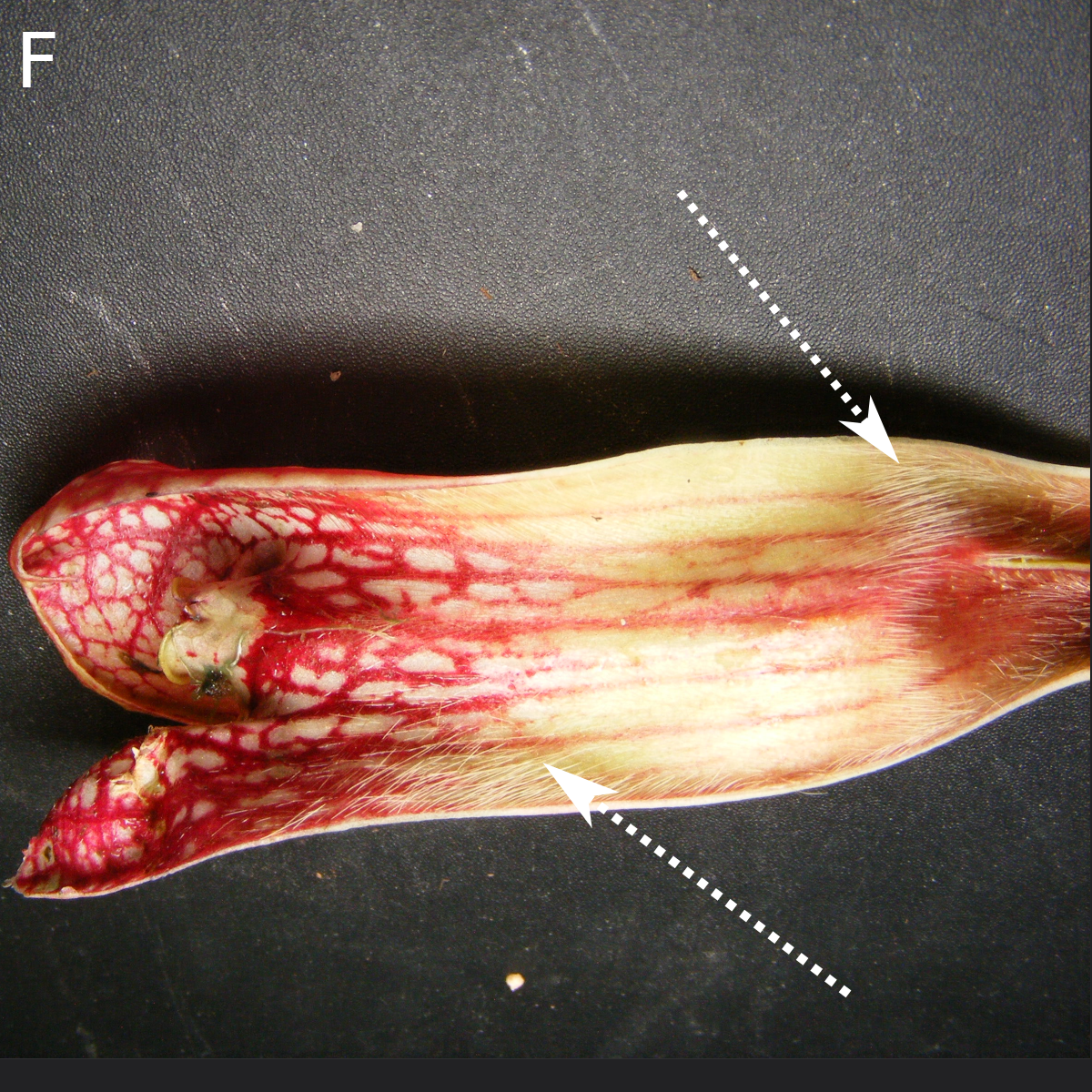

Supplement: Supplementary file 4 [file LSA-2018-00146_SdataF4.zip › Fig4F.tif]

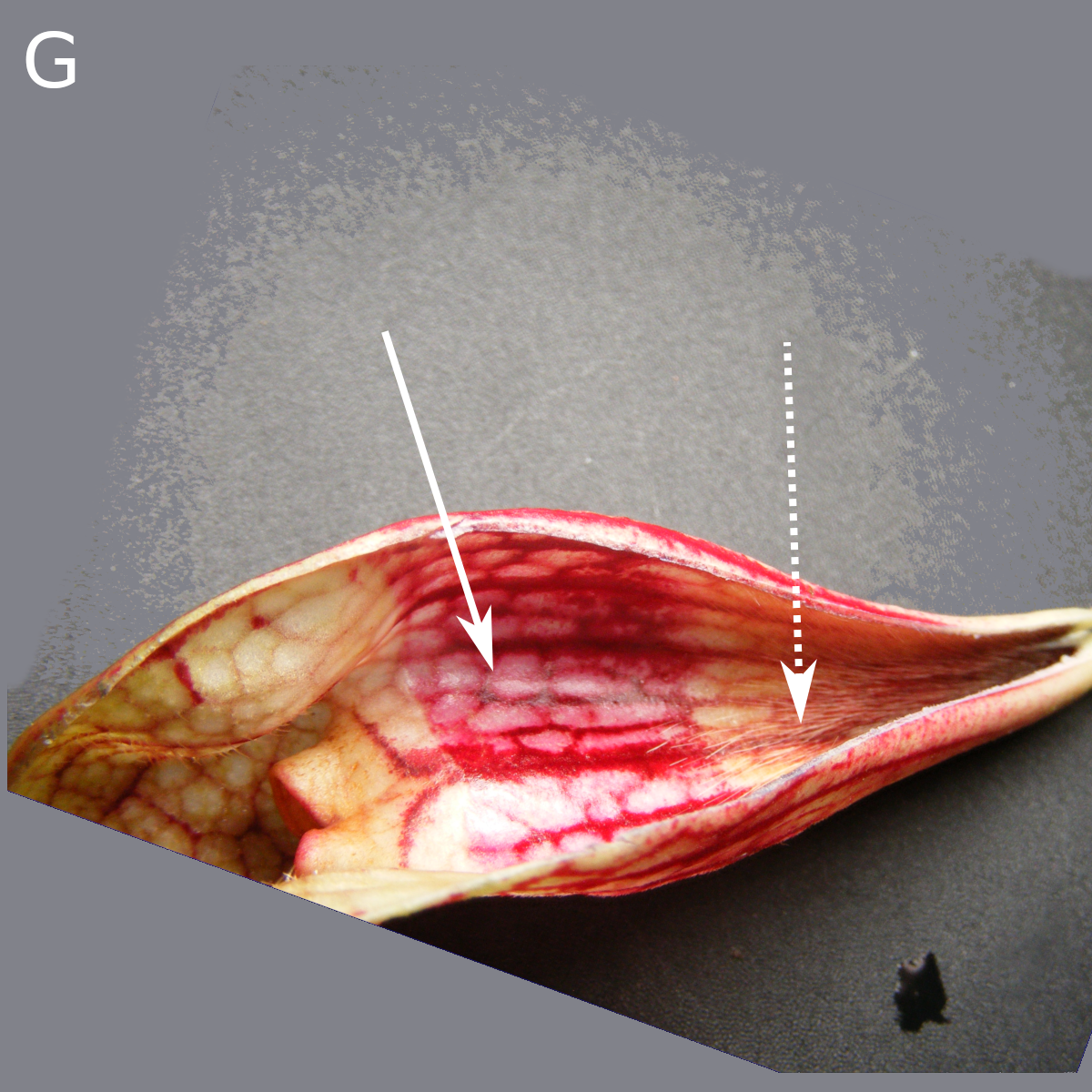

Supplement: Supplementary file 4 [file LSA-2018-00146_SdataF4.zip › Fig4G.tif]

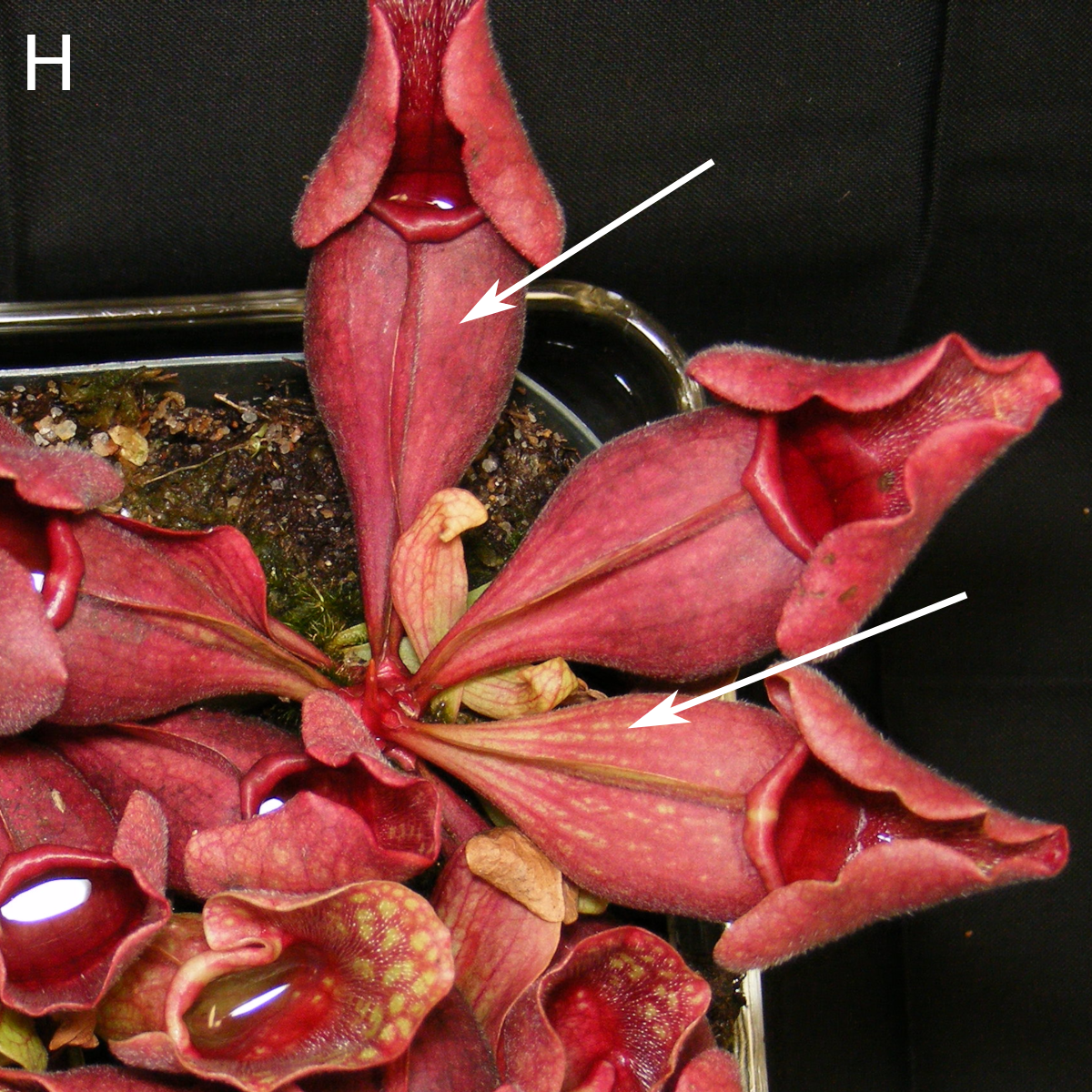

Supplement: Supplementary file 4 [file LSA-2018-00146_SdataF4.zip › Fig4H.tif]

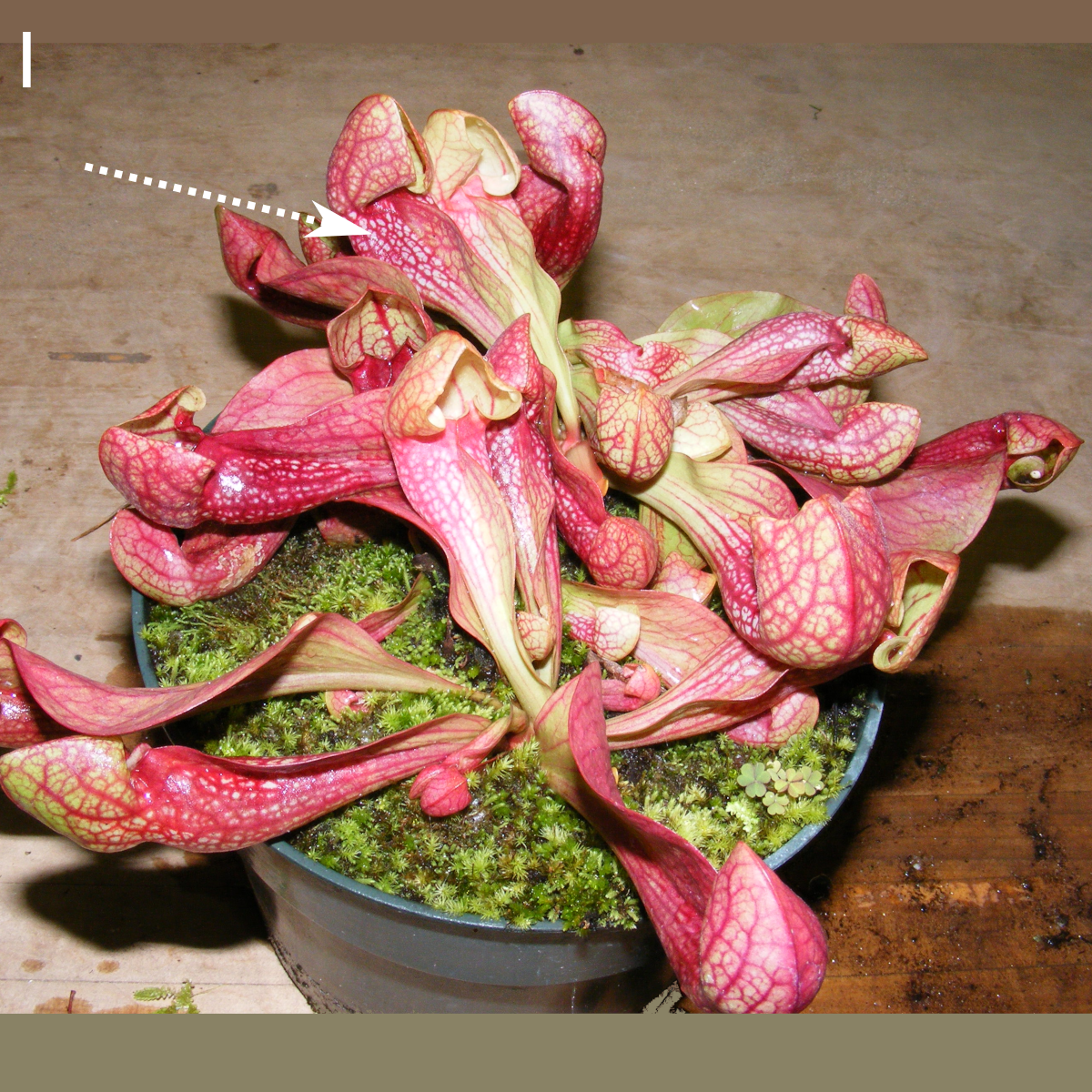

Supplement: Supplementary file 4 [file LSA-2018-00146_SdataF4.zip › Fig4I.tif]

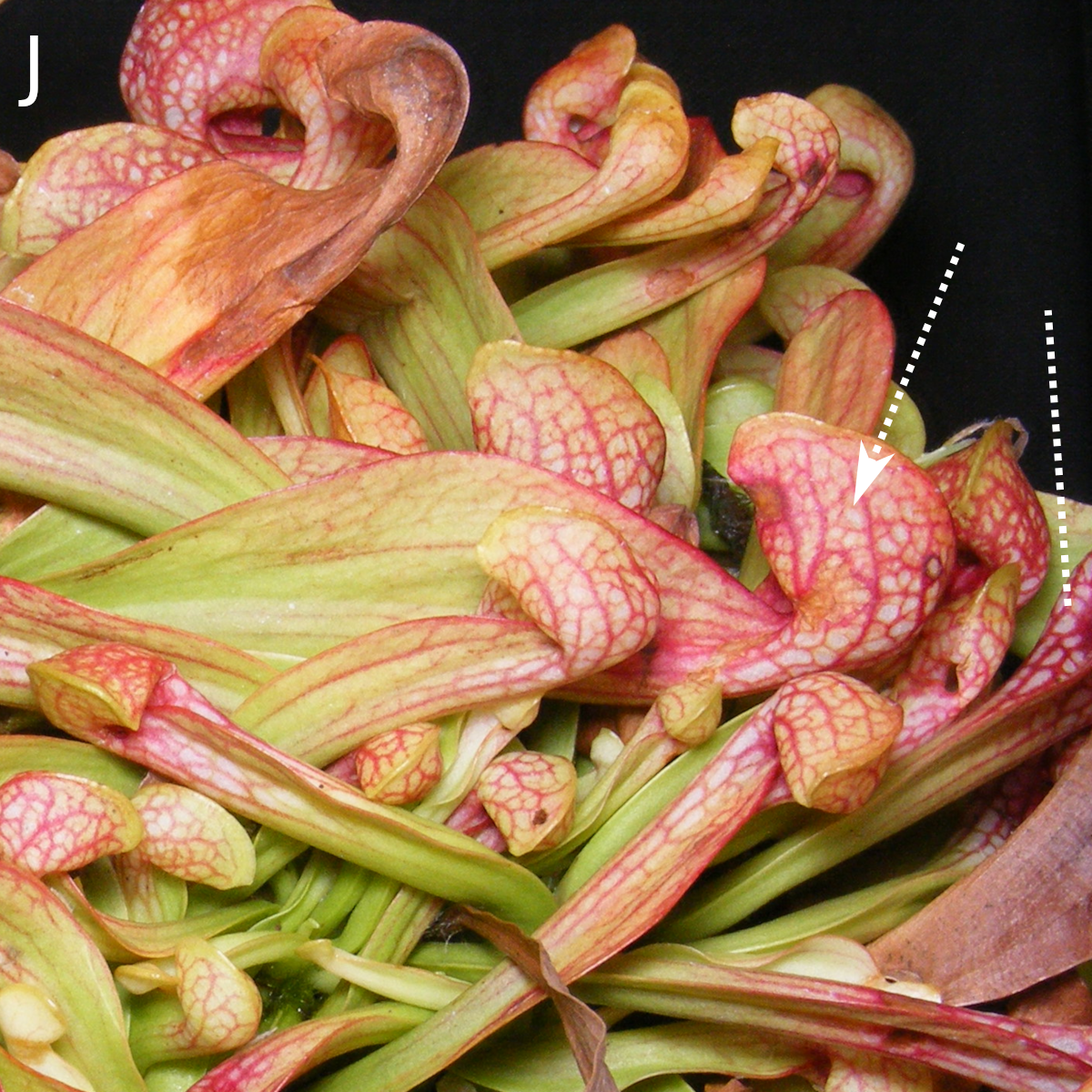

Supplement: Supplementary file 4 [file LSA-2018-00146_SdataF4.zip › Fig4J.tif]

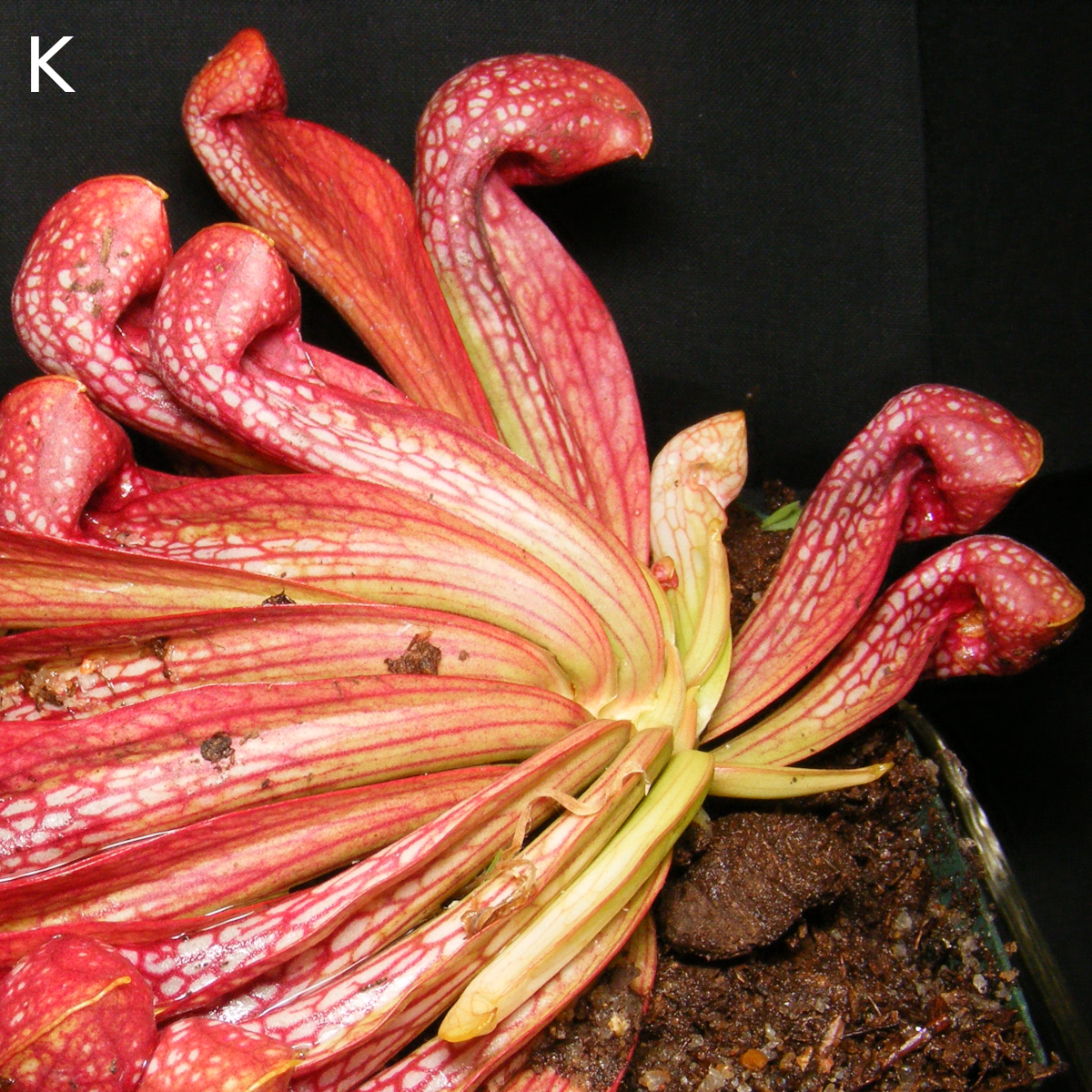

Supplement: Supplementary file 4 [file LSA-2018-00146_SdataF4.zip › Fig4K.tif]

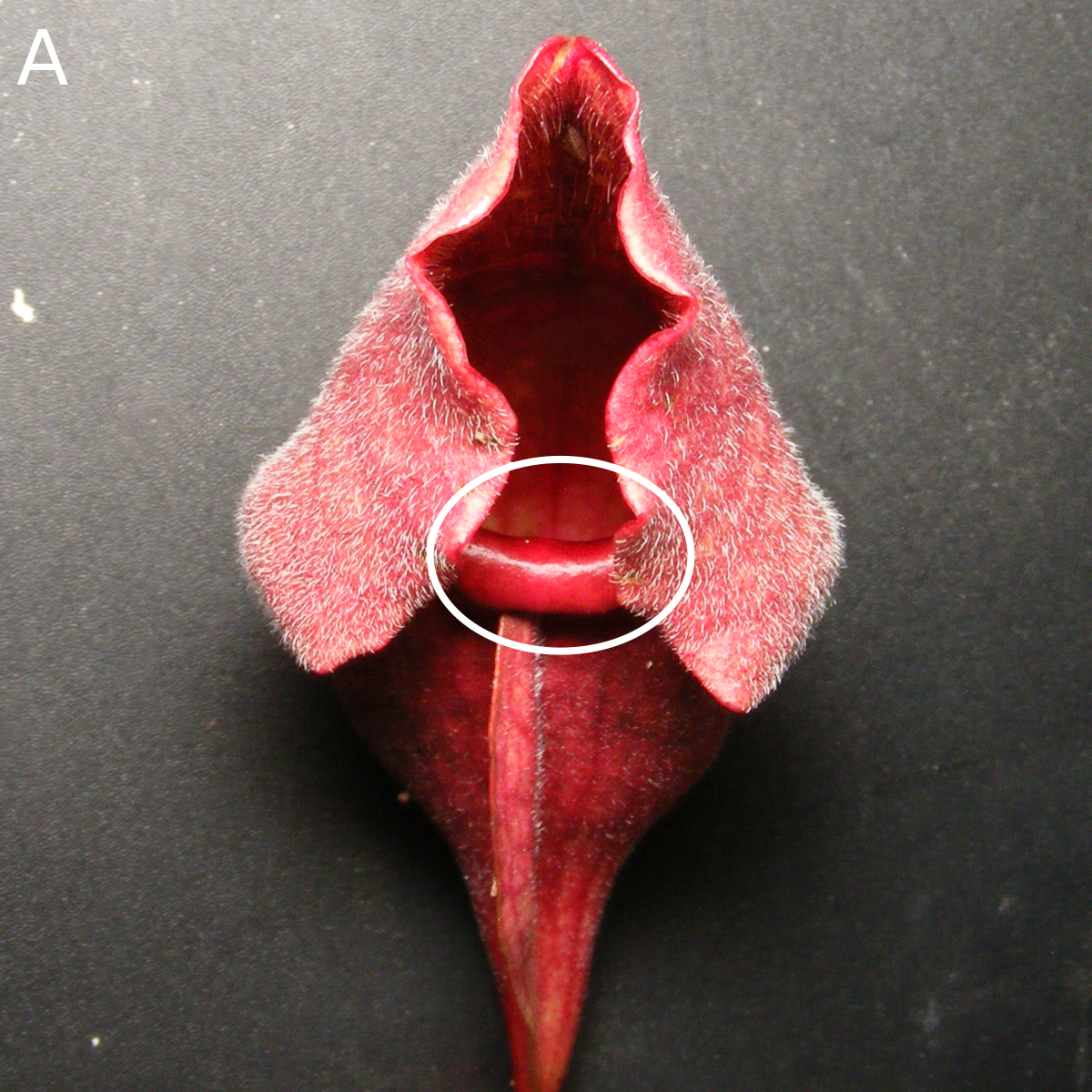

Supplement: Supplementary file 5 [file LSA-2018-00146_SdataF5.zip › Fig5A.tif]

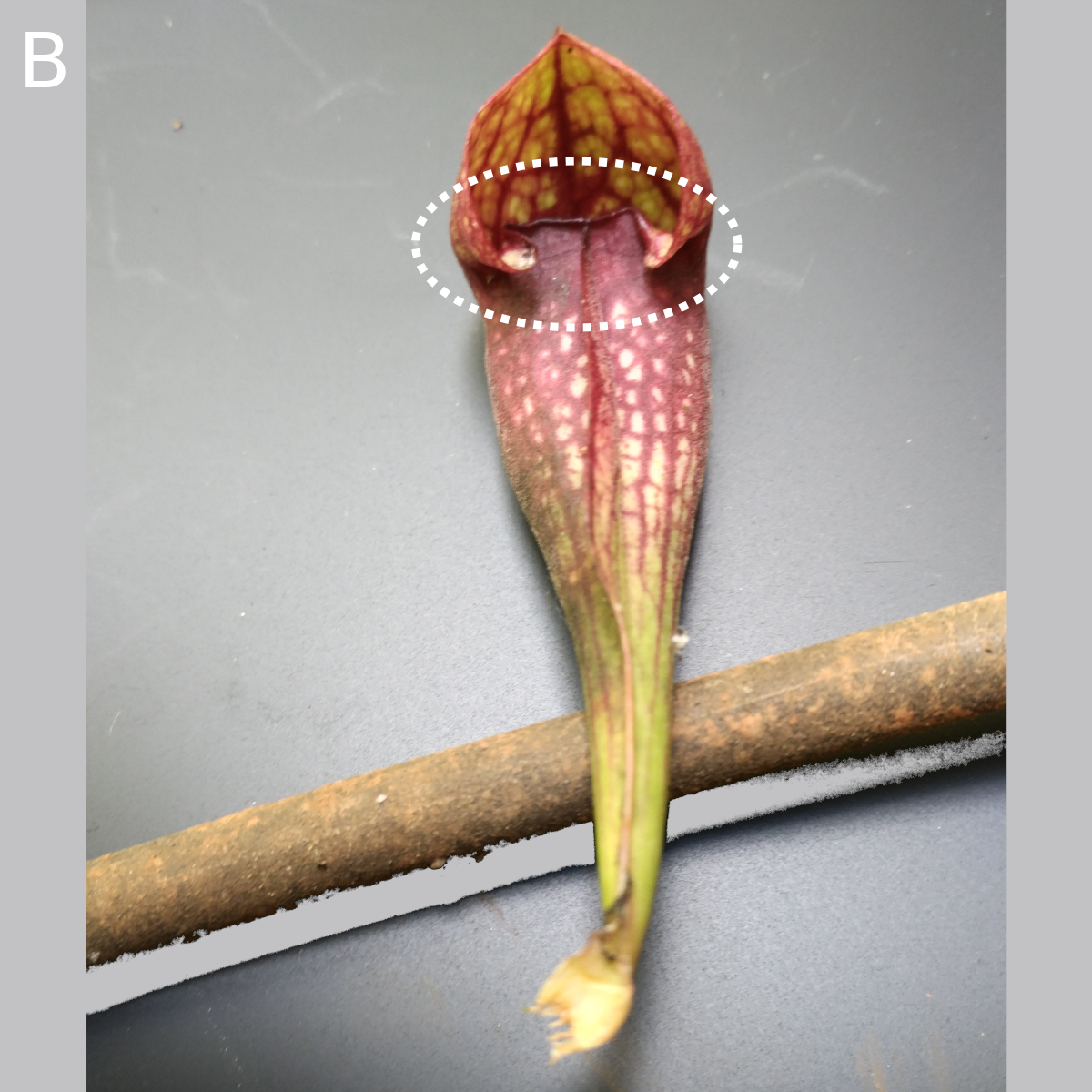

Supplement: Supplementary file 5 [file LSA-2018-00146_SdataF5.zip › Fig5B.tif]

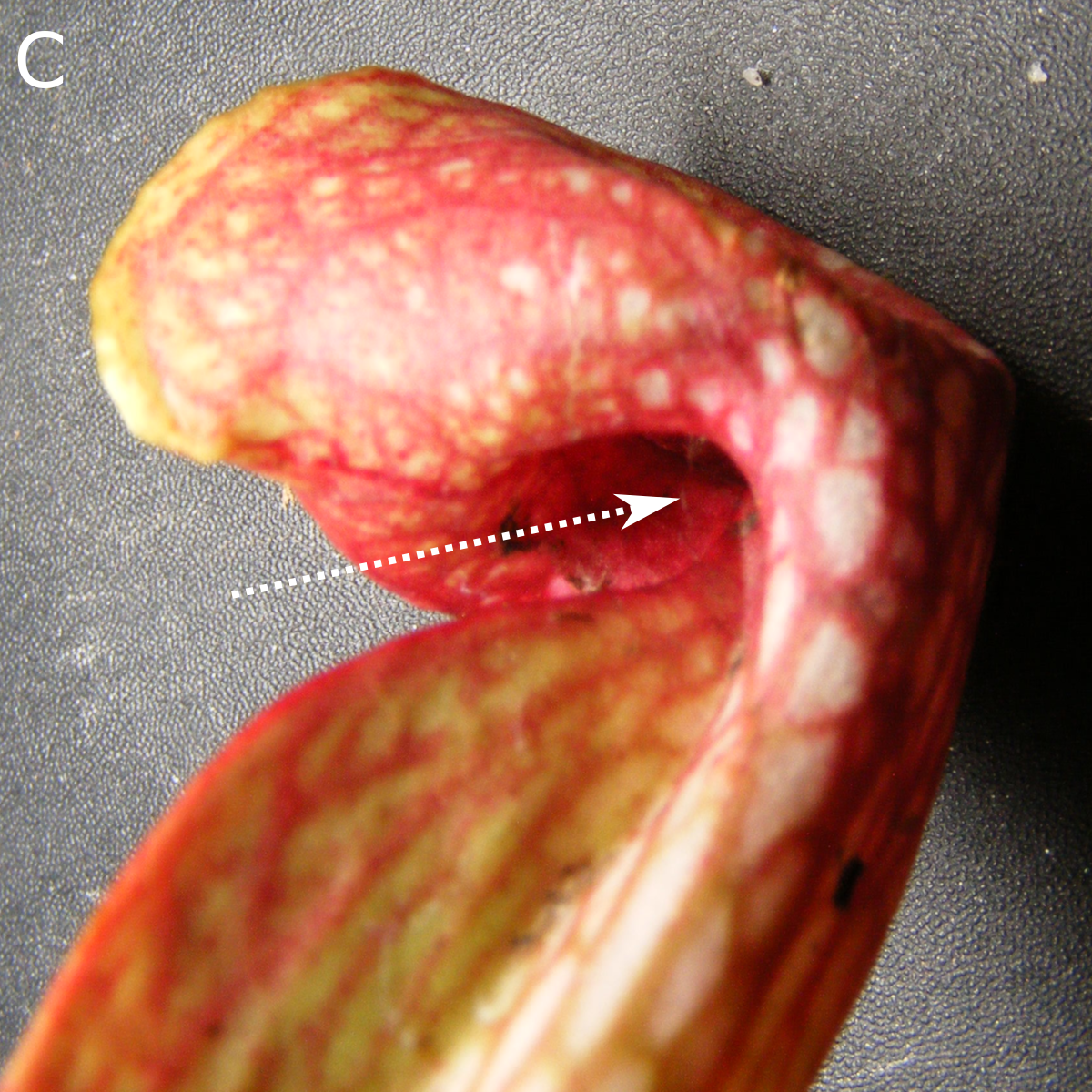

Supplement: Supplementary file 5 [file LSA-2018-00146_SdataF5.zip › Fig5C.tif]

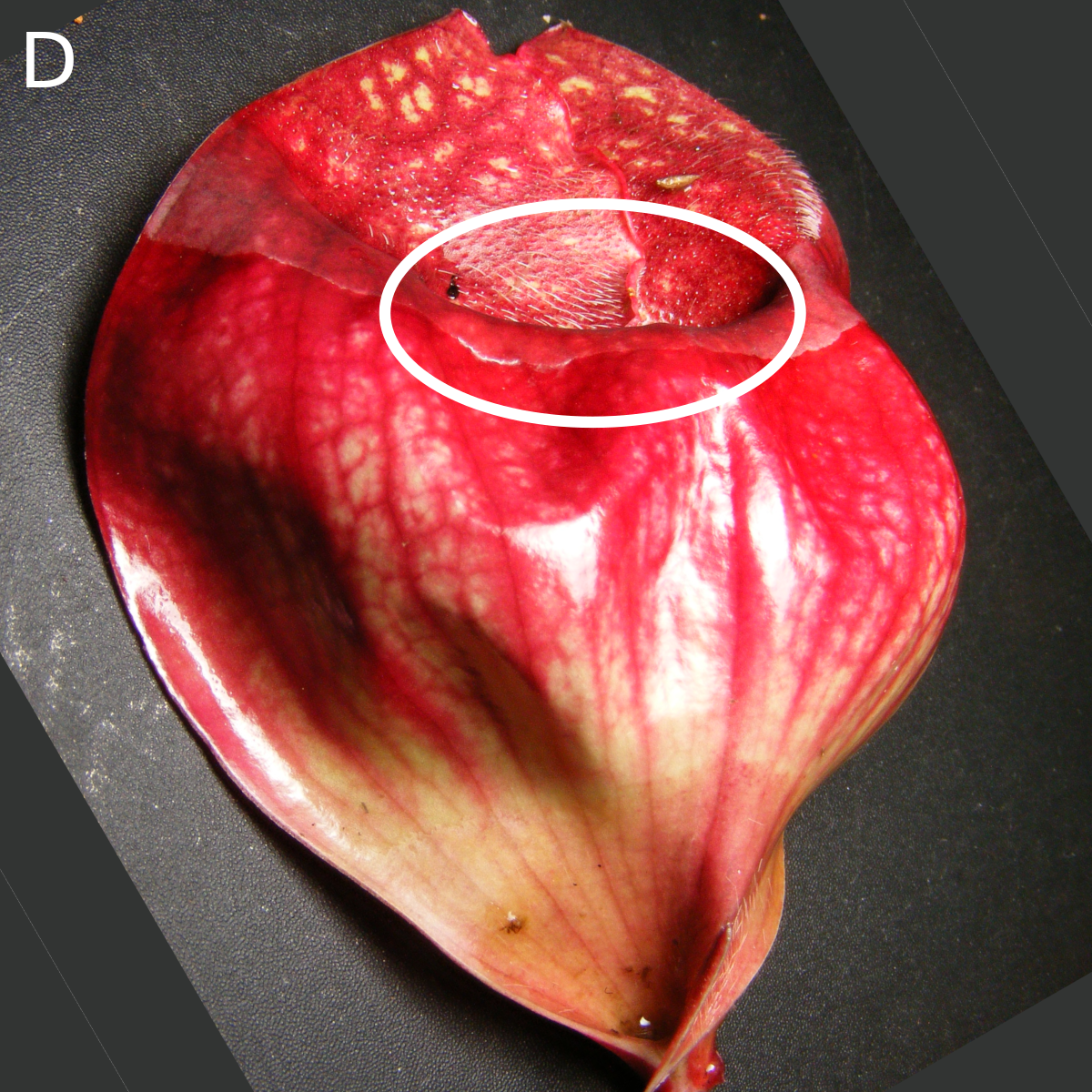

Supplement: Supplementary file 5 [file LSA-2018-00146_SdataF5.zip › Fig5D.tif]

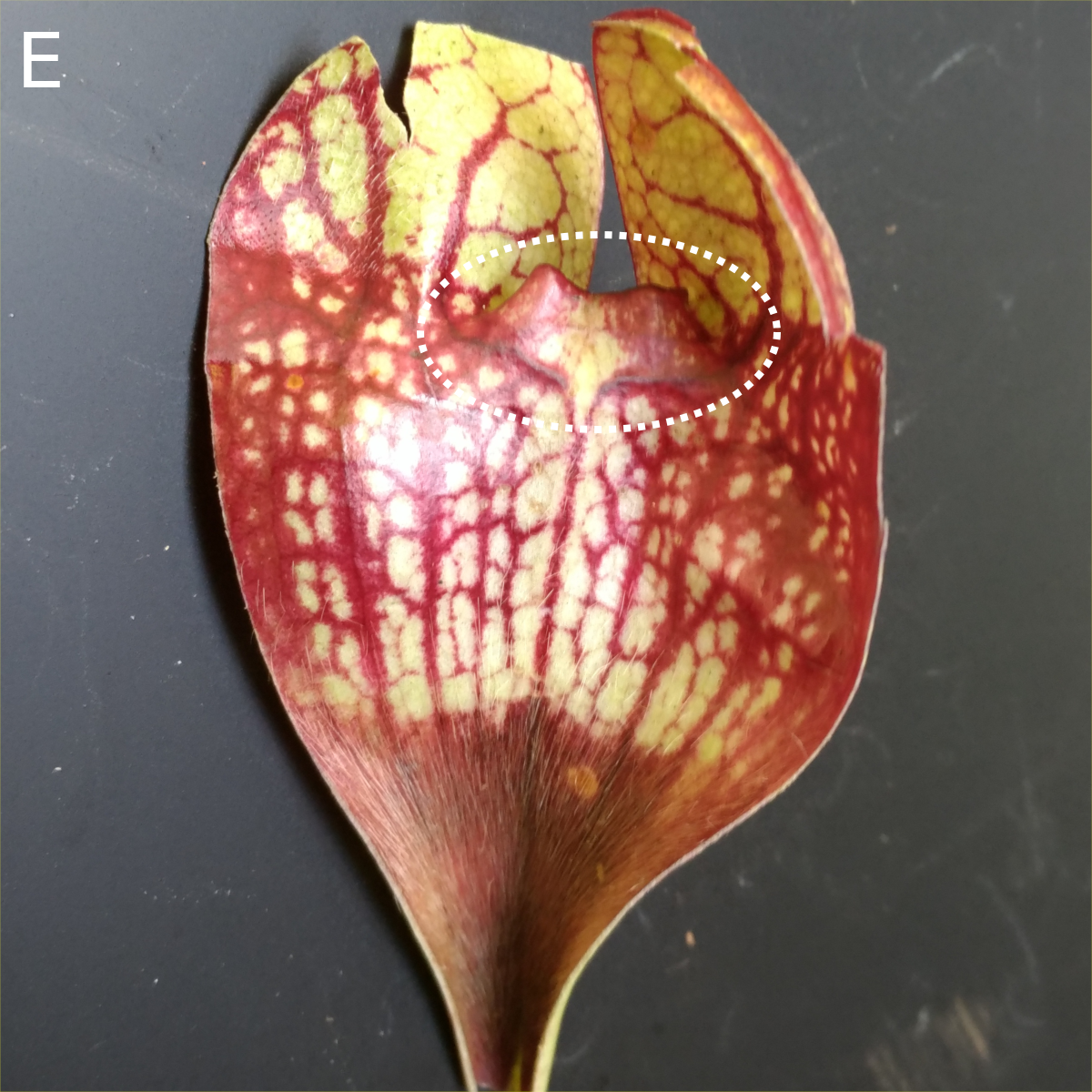

Supplement: Supplementary file 5 [file LSA-2018-00146_SdataF5.zip › Fig5E.tif]

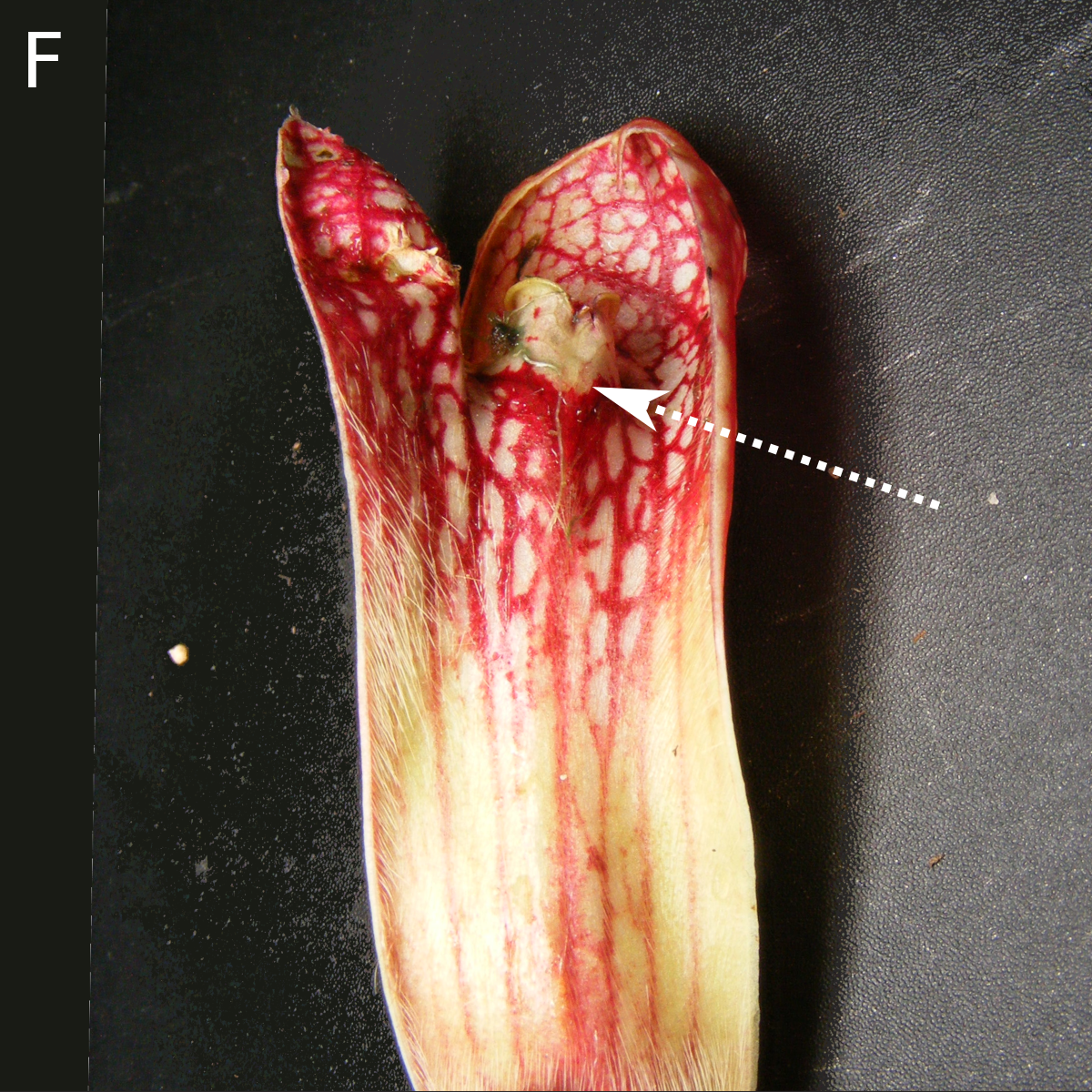

Supplement: Supplementary file 5 [file LSA-2018-00146_SdataF5.zip › Fig5F.tif]

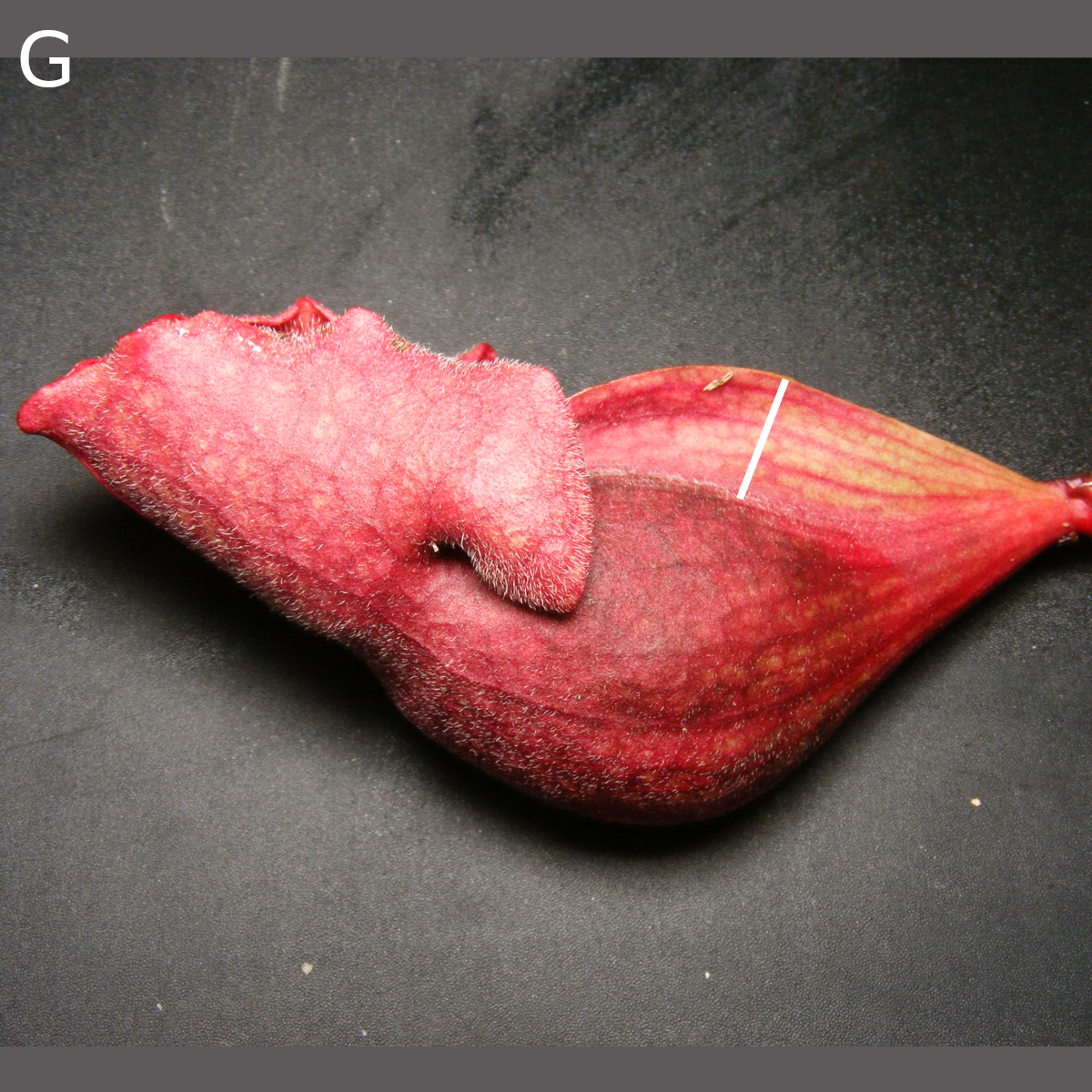

Supplement: Supplementary file 5 [file LSA-2018-00146_SdataF5.zip › Fig5G.tif]

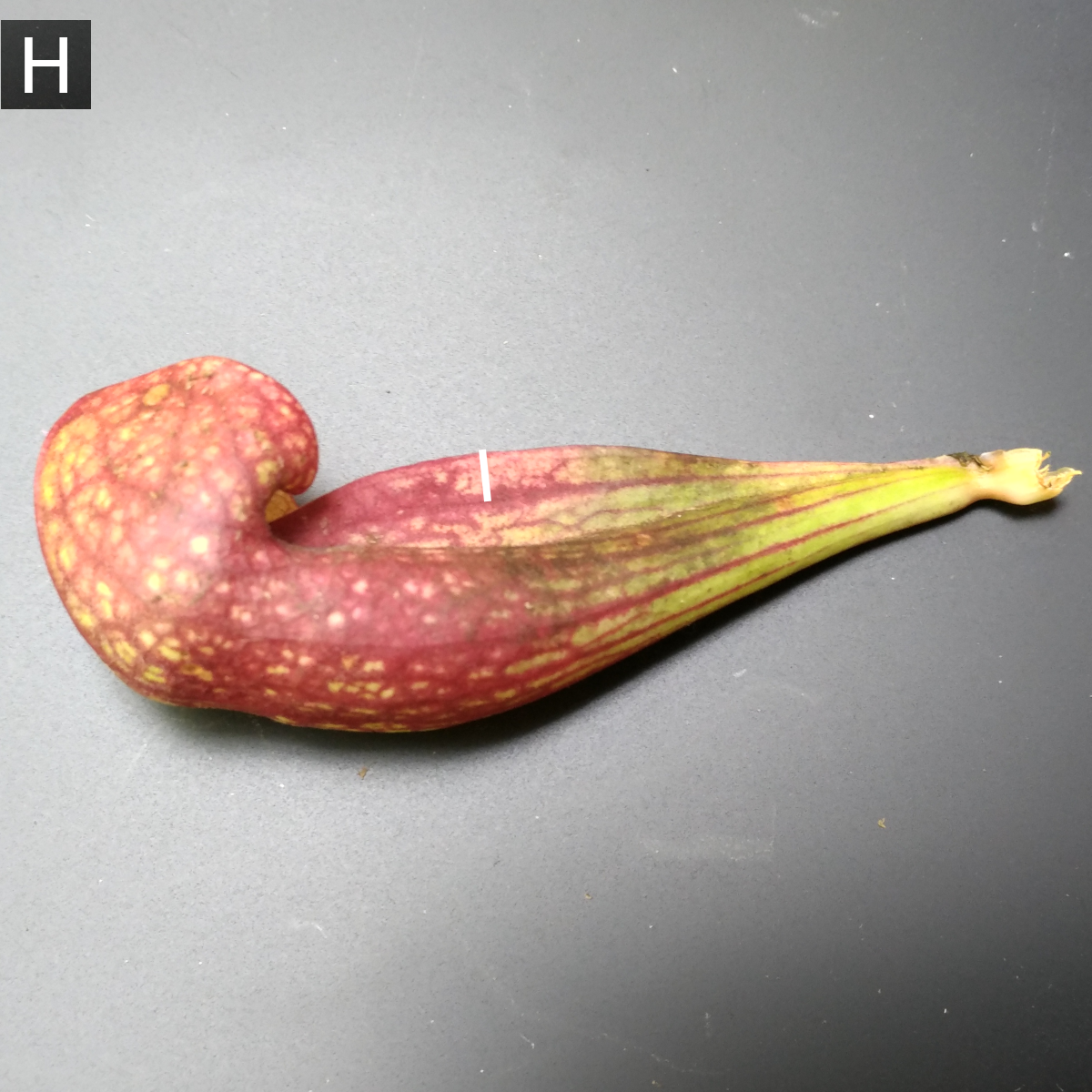

Supplement: Supplementary file 5 [file LSA-2018-00146_SdataF5.zip › Fig5H.tif]

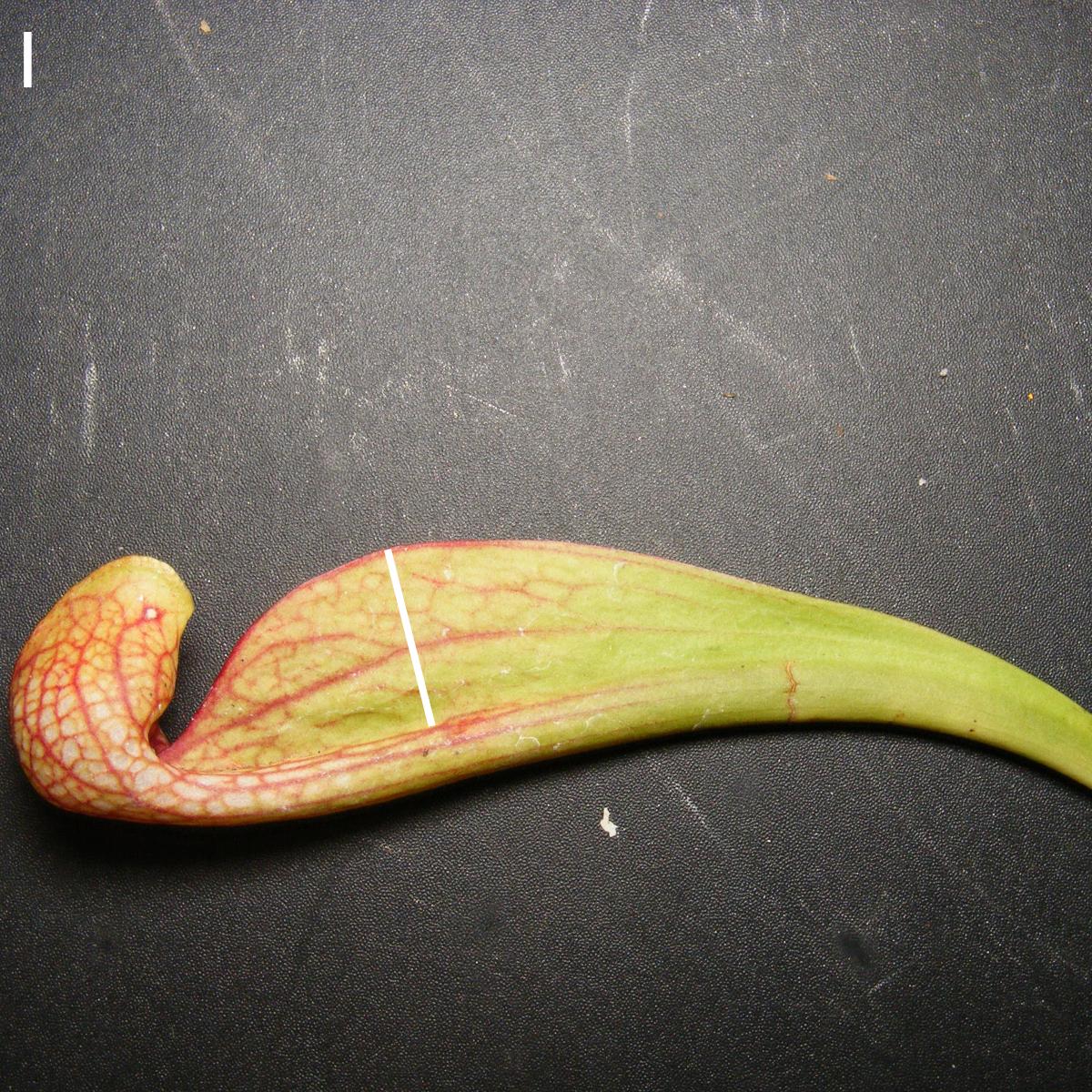

Supplement: Supplementary file 5 [file LSA-2018-00146_SdataF5.zip › Fig5I.tif]

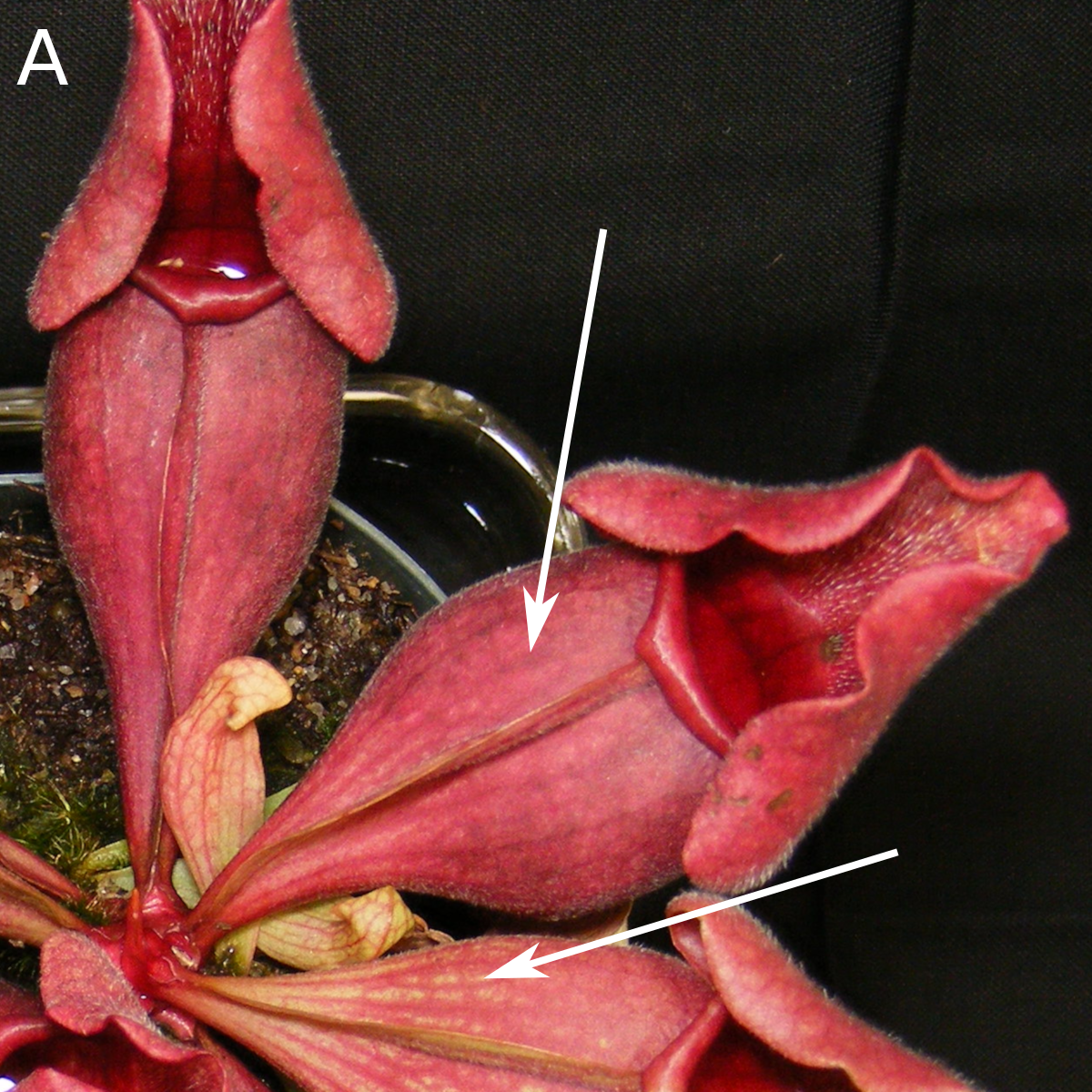

Supplement: Supplementary file 6 [file LSA-2018-00146_SdataF6.zip › Fig6A.tif]

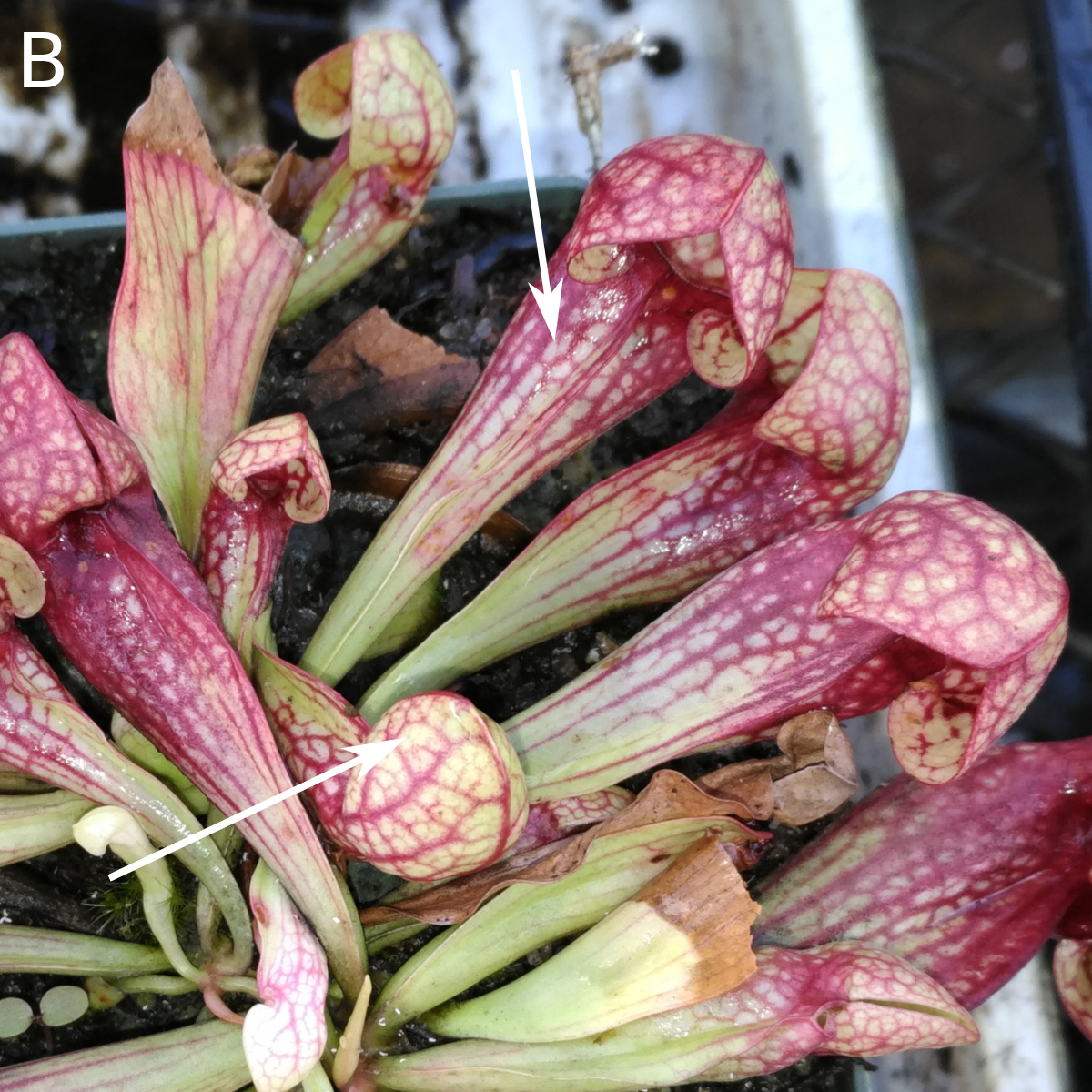

Supplement: Supplementary file 6 [file LSA-2018-00146_SdataF6.zip › Fig6B.tif]

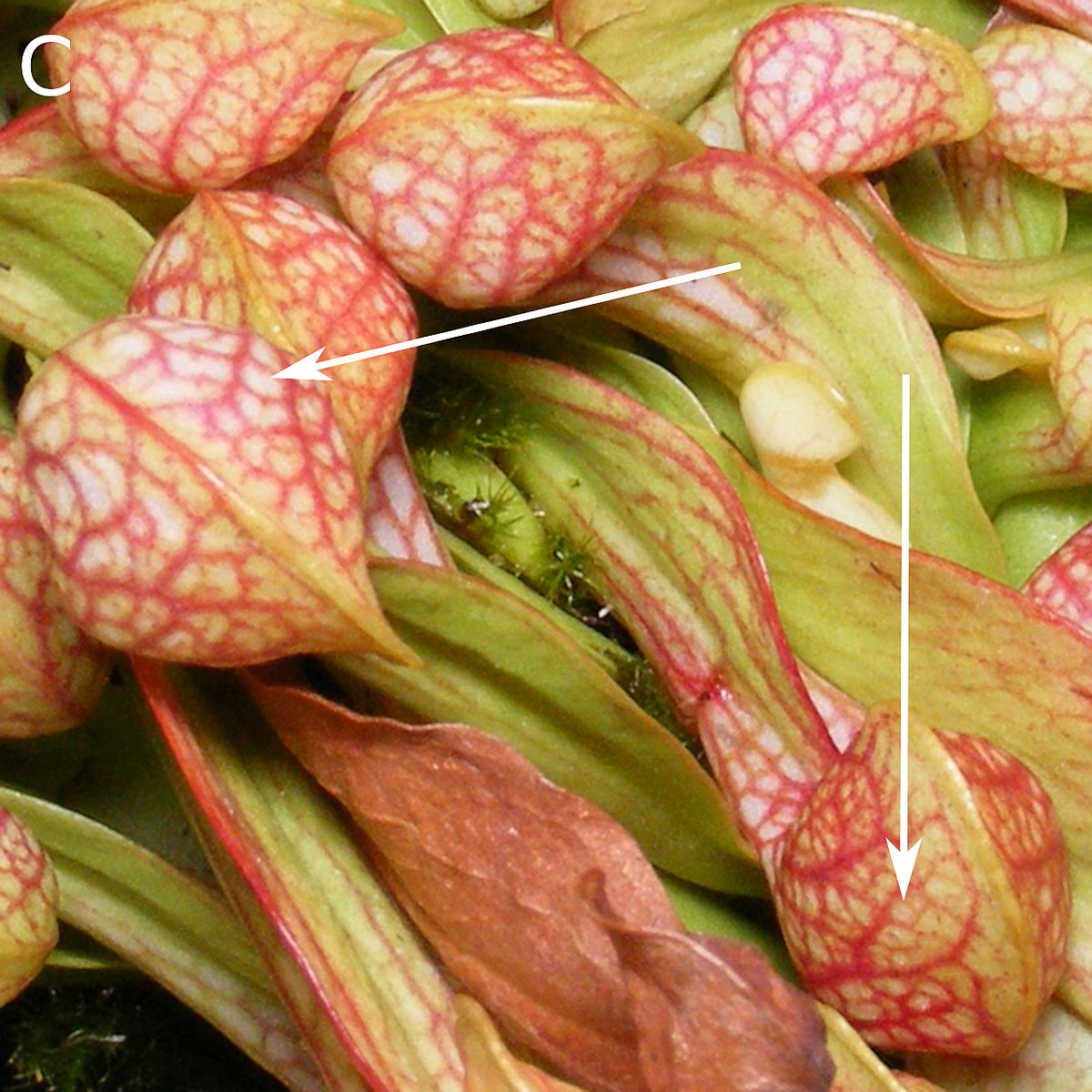

Supplement: Supplementary file 6 [file LSA-2018-00146_SdataF6.zip › Fig6C.tif]

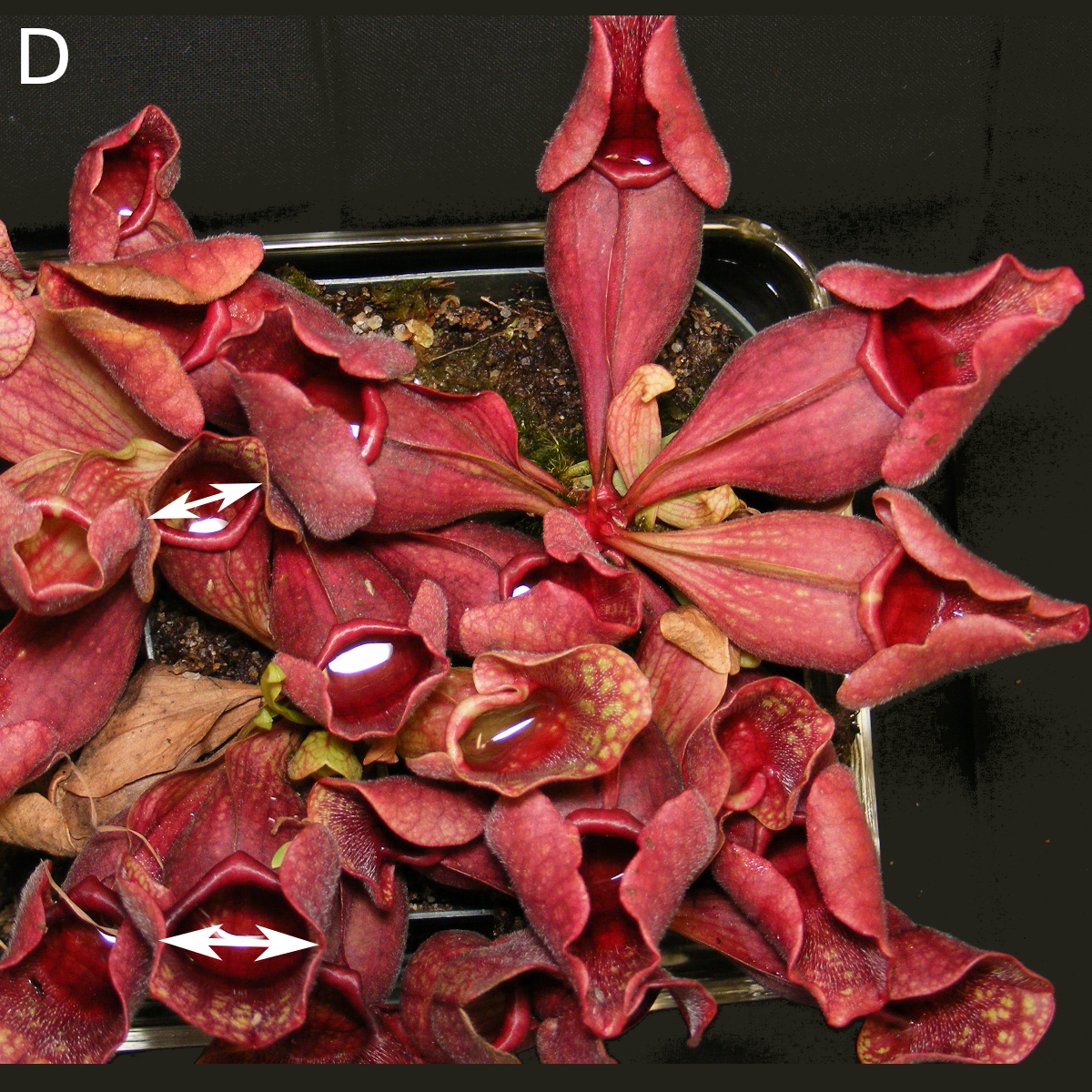

Supplement: Supplementary file 6 [file LSA-2018-00146_SdataF6.zip › Fig6D.tif]

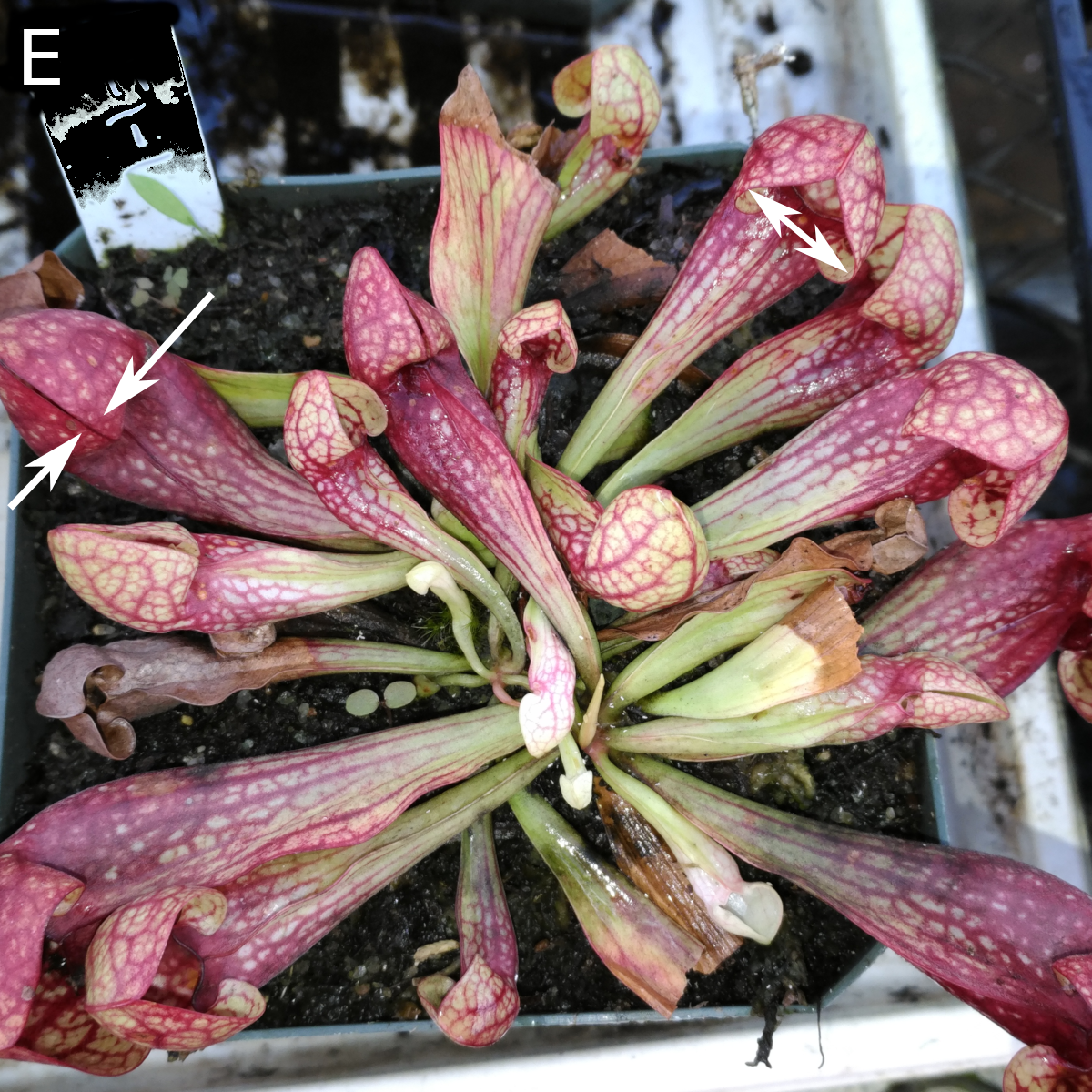

Supplement: Supplementary file 6 [file LSA-2018-00146_SdataF6.zip › Fig6E.tif]

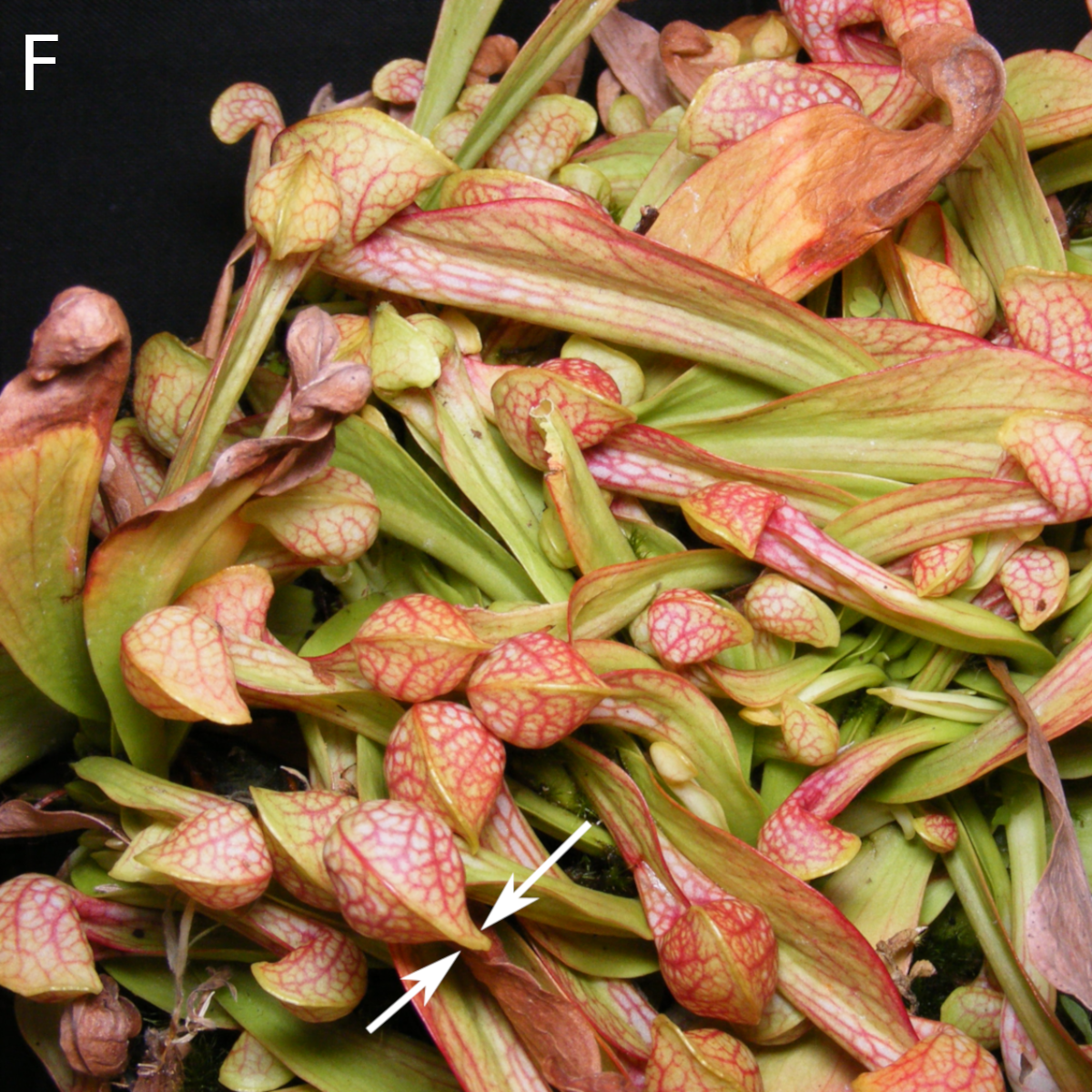

Supplement: Supplementary file 6 [file LSA-2018-00146_SdataF6.zip › Fig6F.tif]

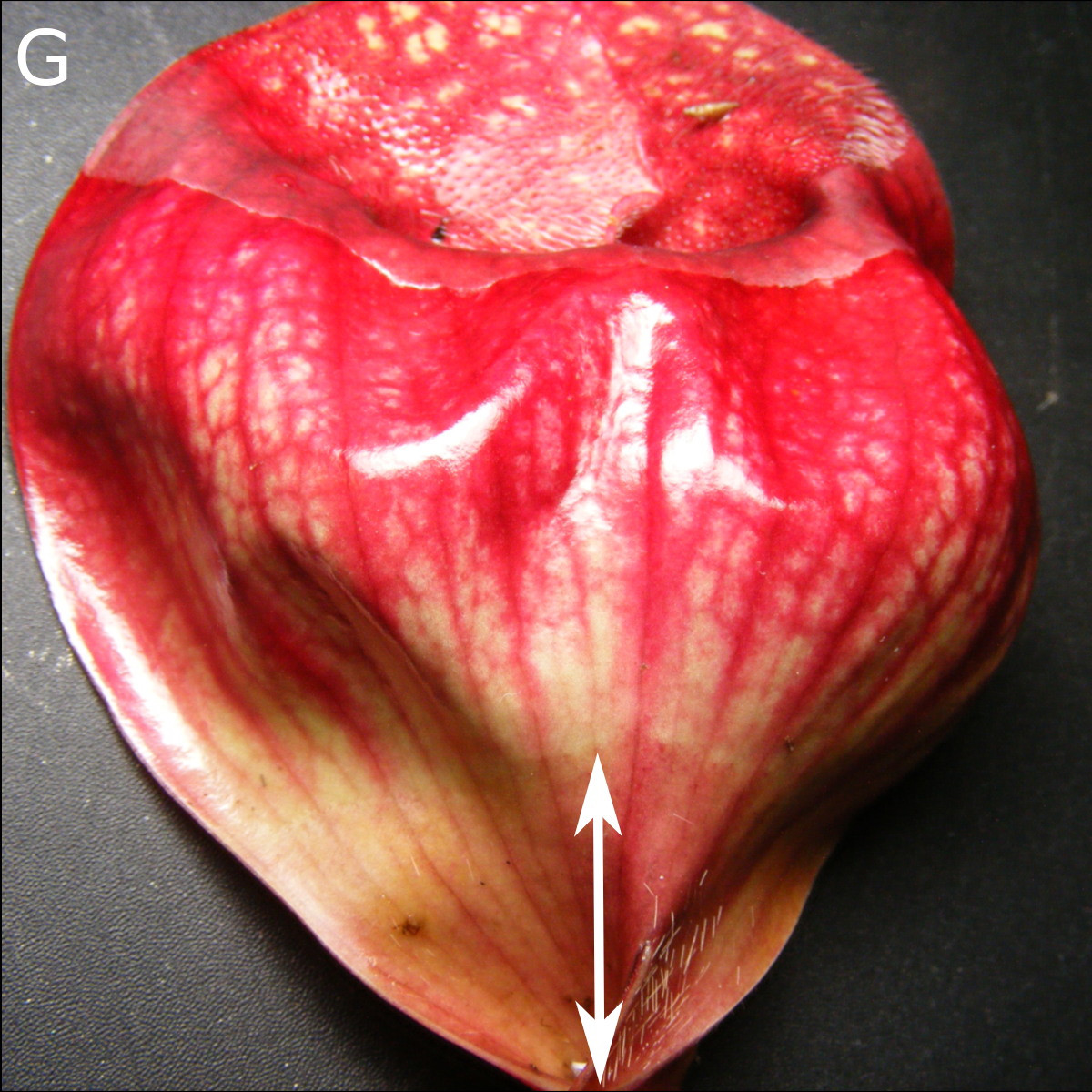

Supplement: Supplementary file 6 [file LSA-2018-00146_SdataF6.zip › Fig6G.tif]

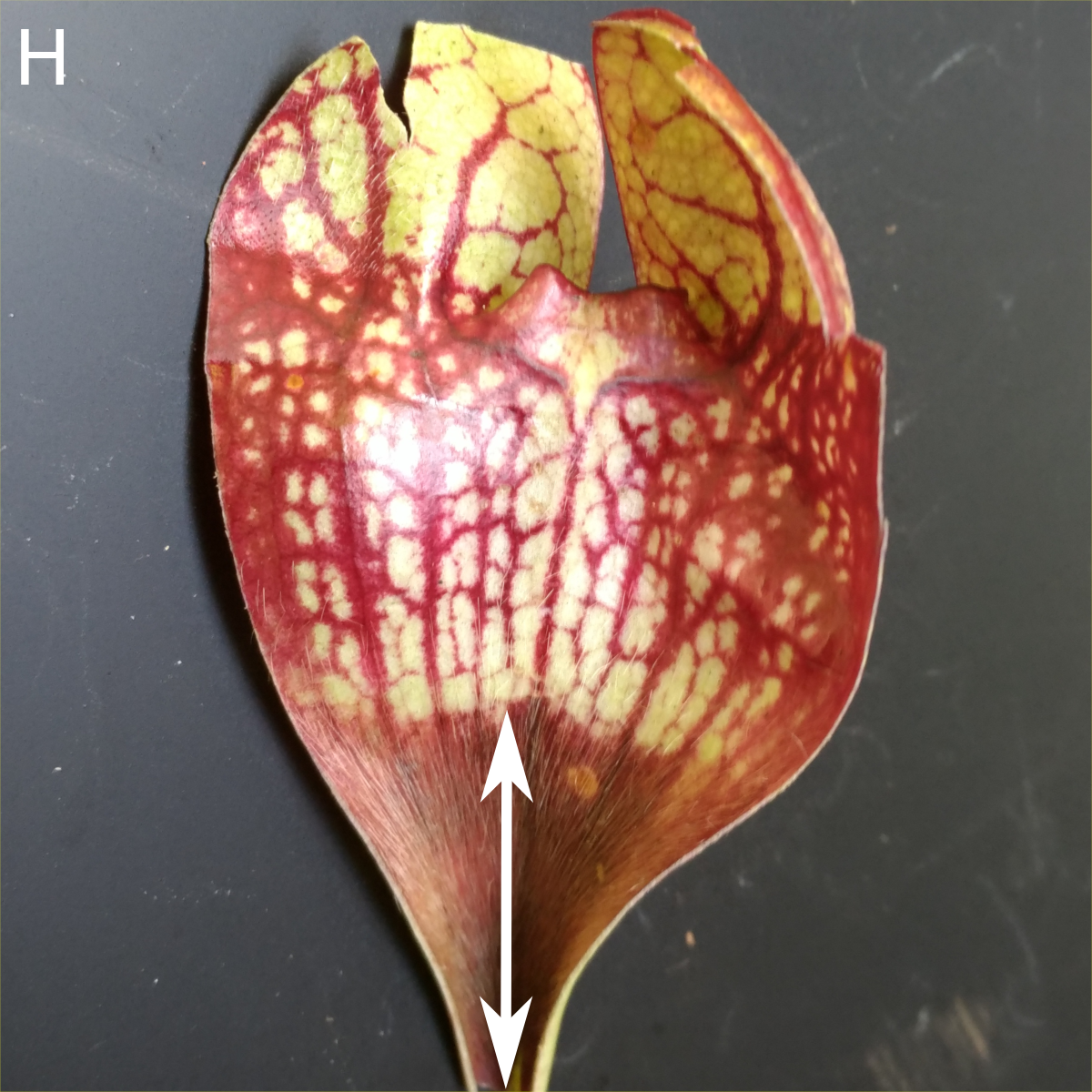

Supplement: Supplementary file 6 [file LSA-2018-00146_SdataF6.zip › Fig6H.tif]

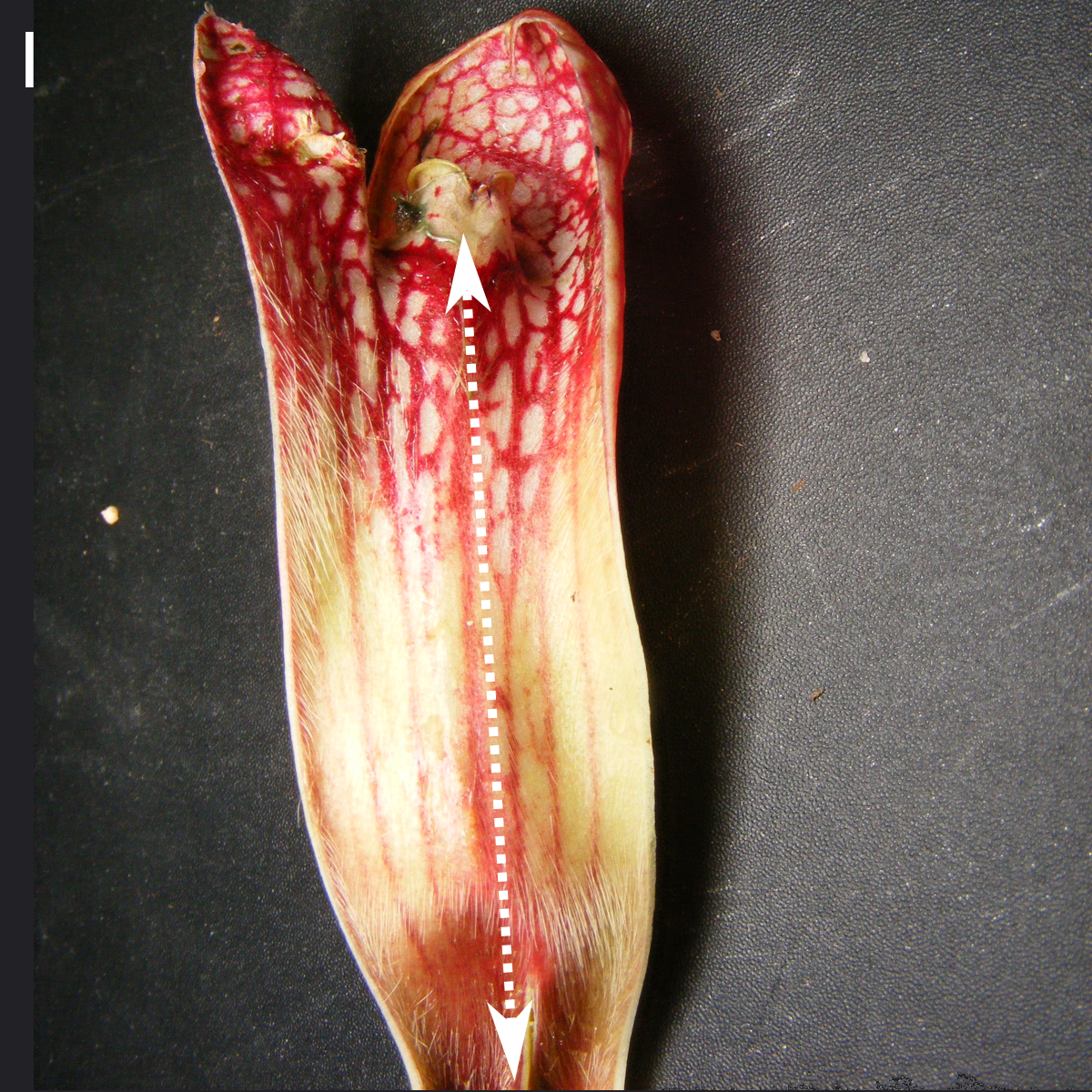

Supplement: Supplementary file 6 [file LSA-2018-00146_SdataF6.zip › Fig6I.tif]

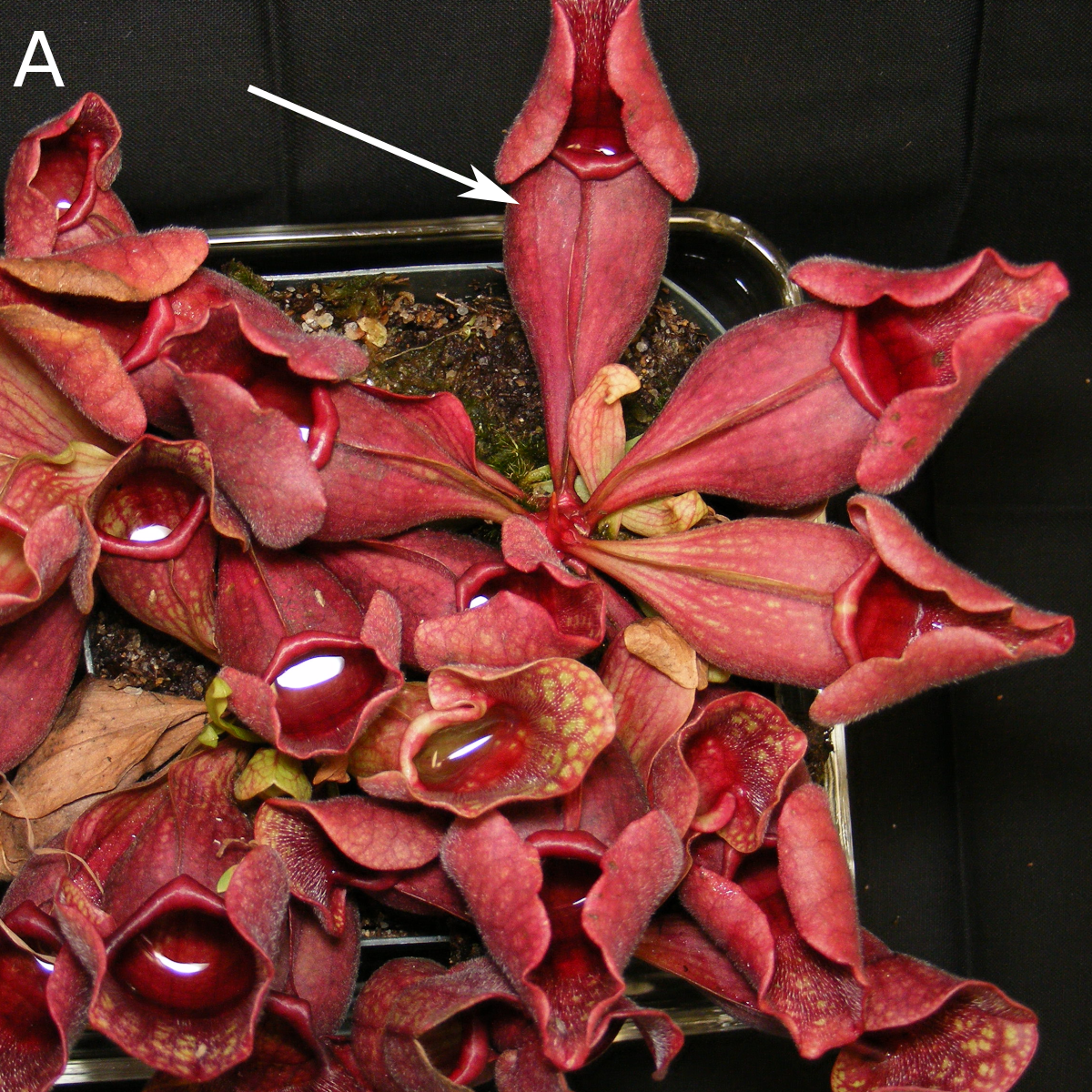

Supplement: Supplementary file 7 [file LSA-2018-00146_SdataF7.zip › Fig7A.tif]

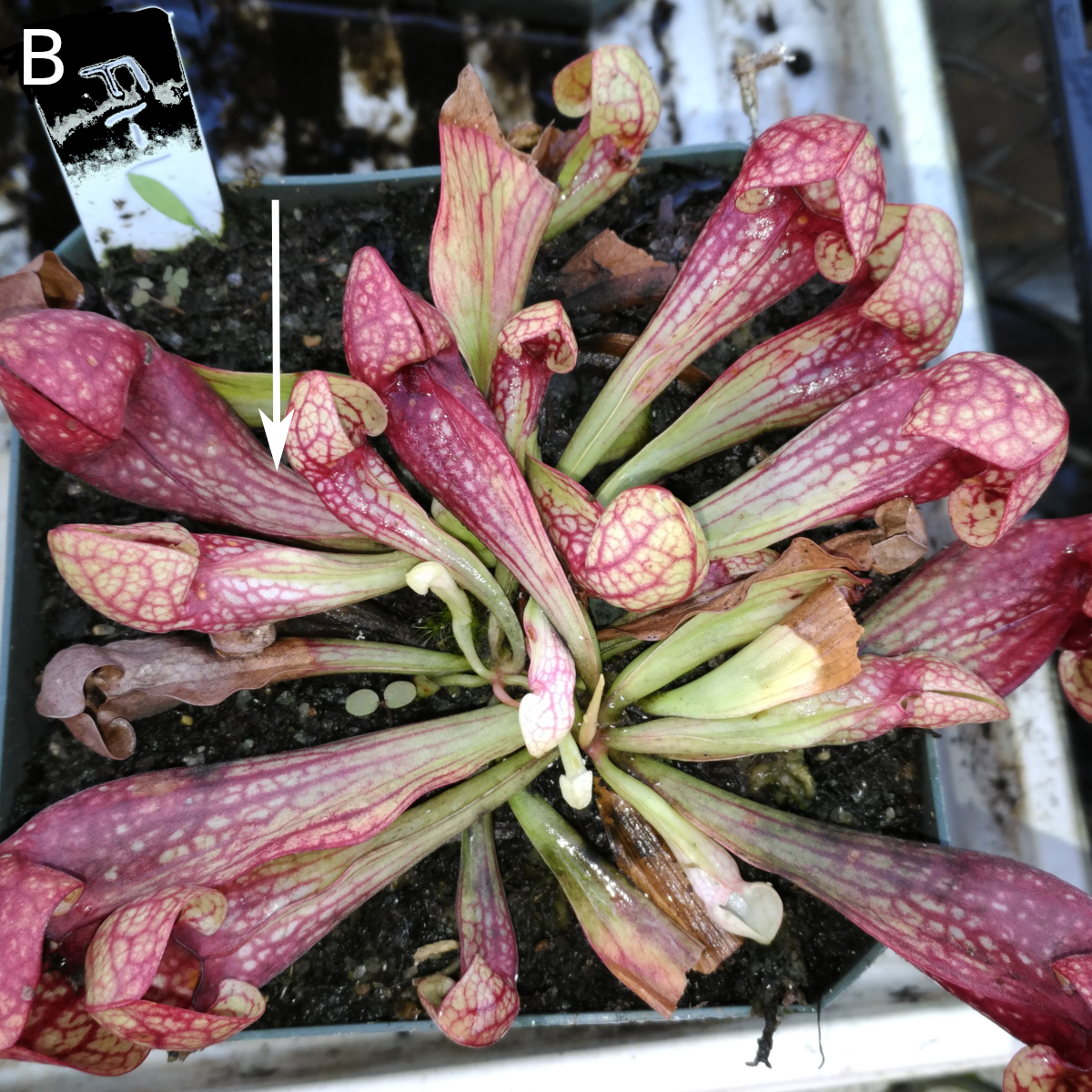

Supplement: Supplementary file 7 [file LSA-2018-00146_SdataF7.zip › Fig7B.tif]

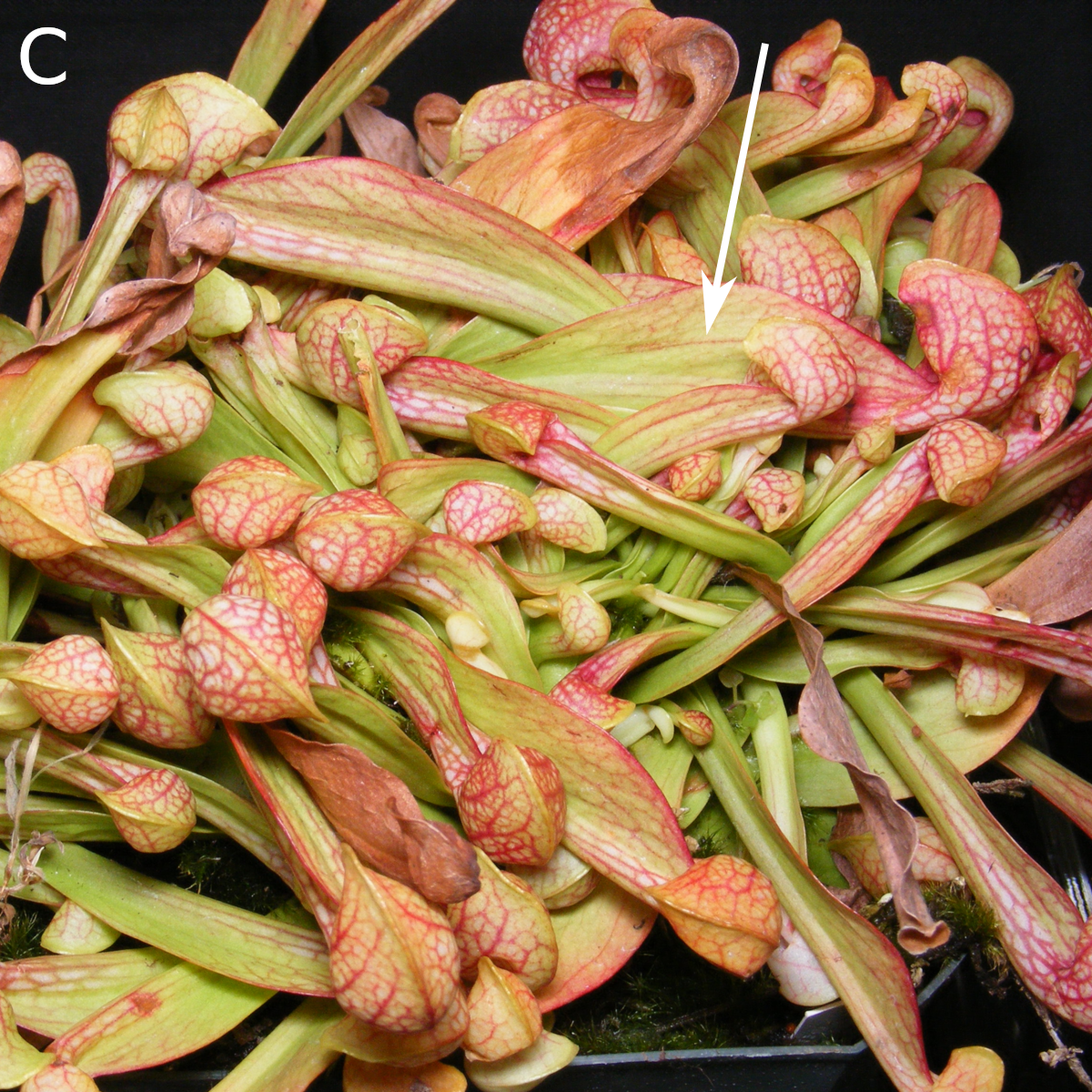

Supplement: Supplementary file 7 [file LSA-2018-00146_SdataF7.zip › Fig7C.tif]

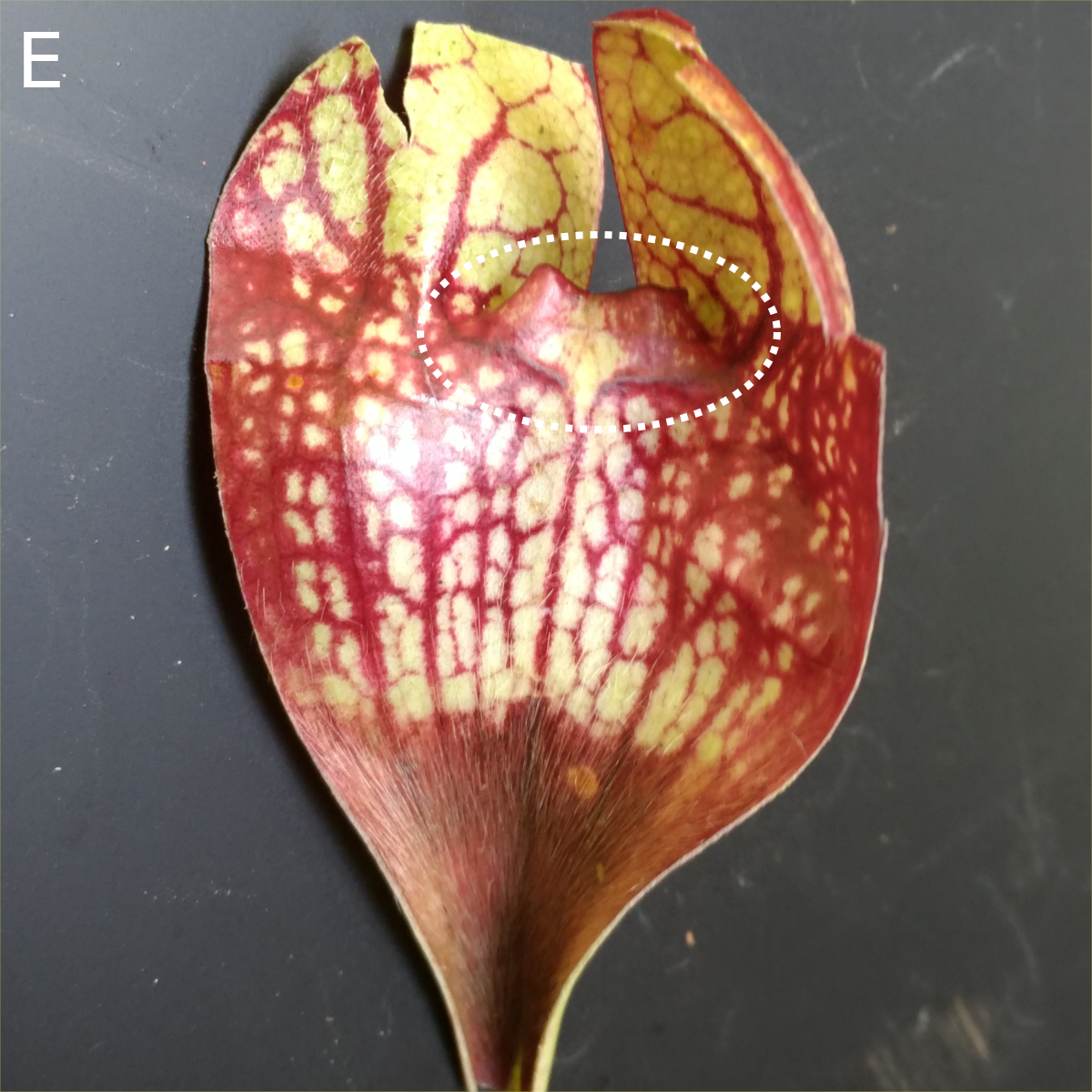

Supplement: Supplementary file 7 [file LSA-2018-00146_SdataF7.zip › Fig7E.tif]

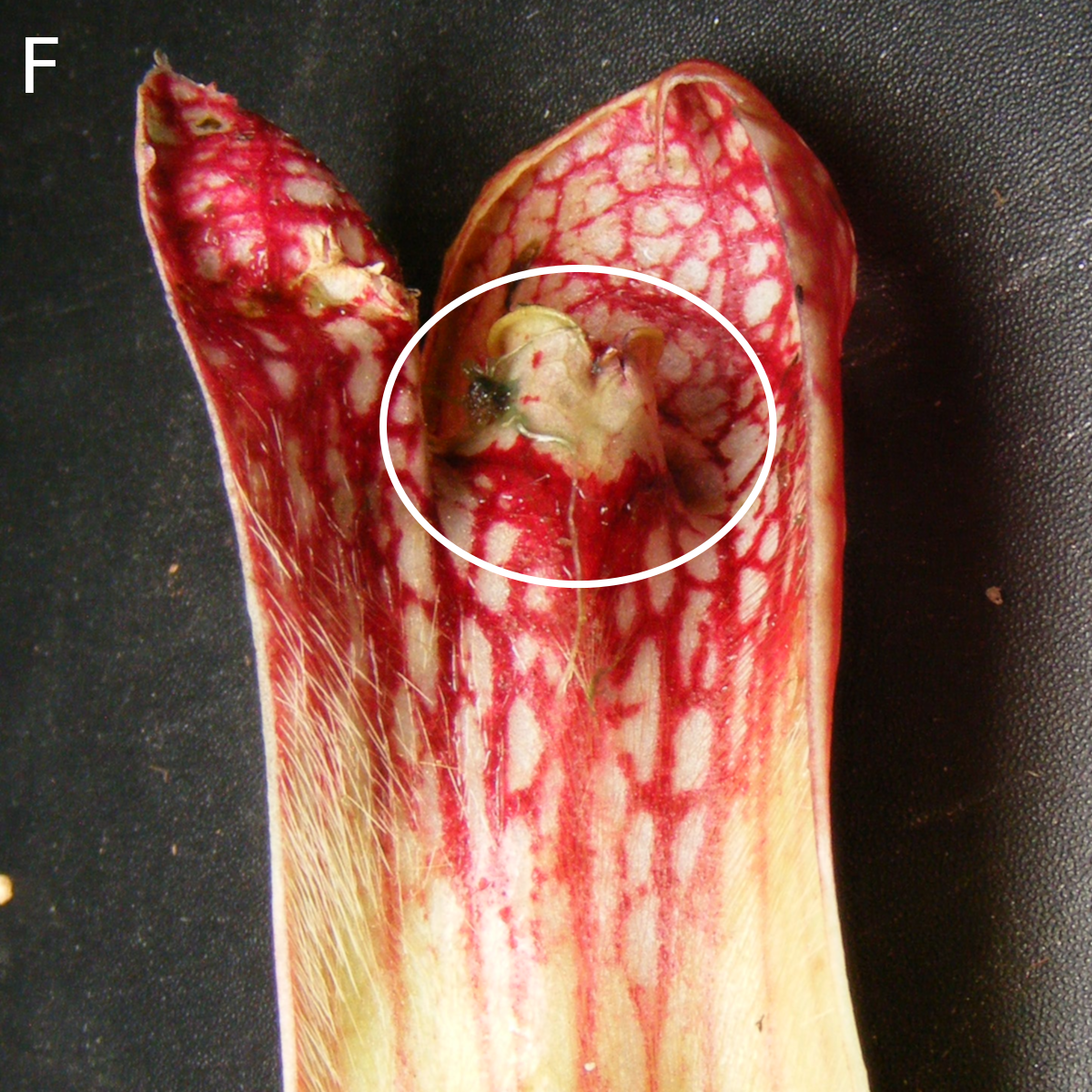

Supplement: Supplementary file 7 [file LSA-2018-00146_SdataF7.zip › Fig7F.tif]

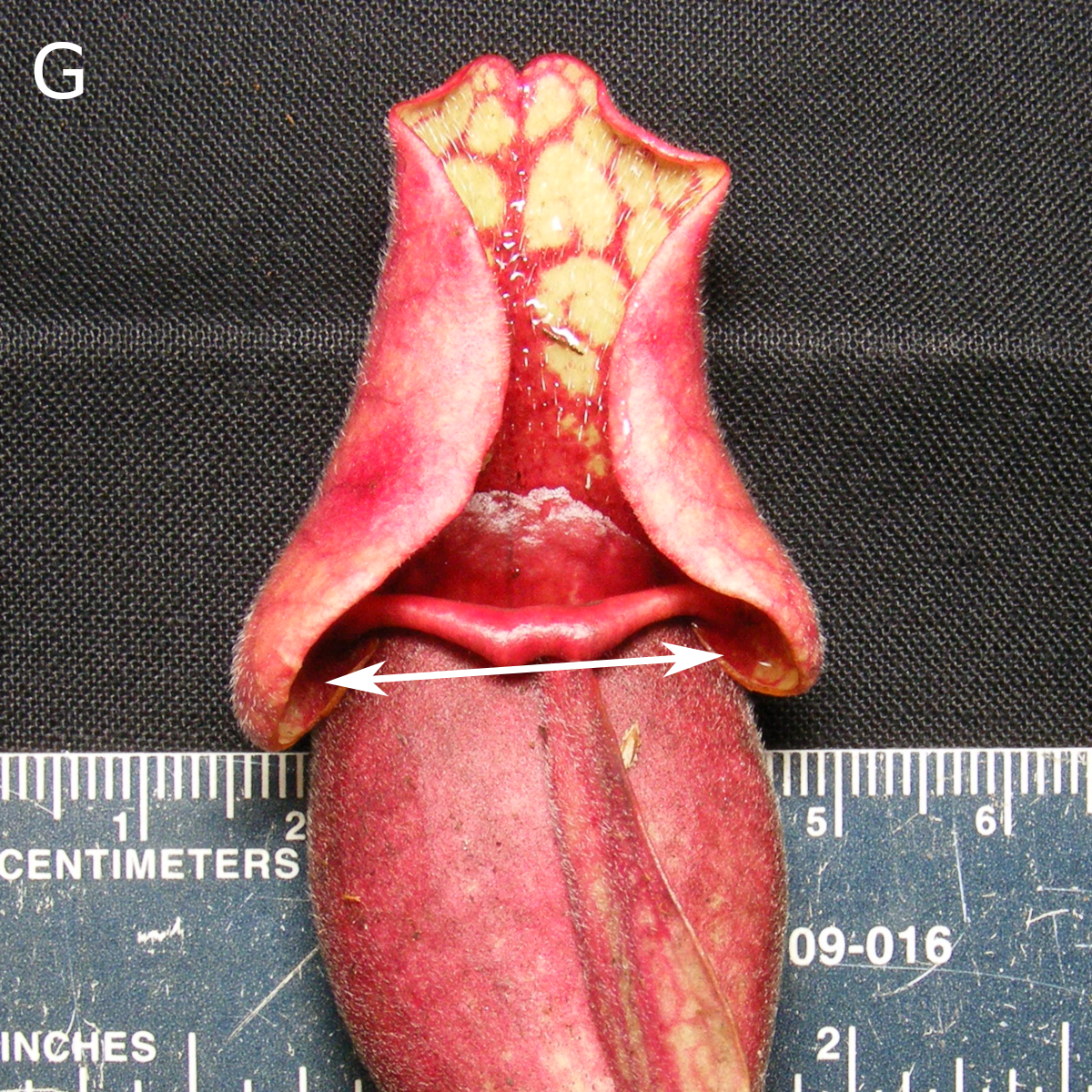

Supplement: Supplementary file 7 [file LSA-2018-00146_SdataF7.zip › Fig7G.tif]

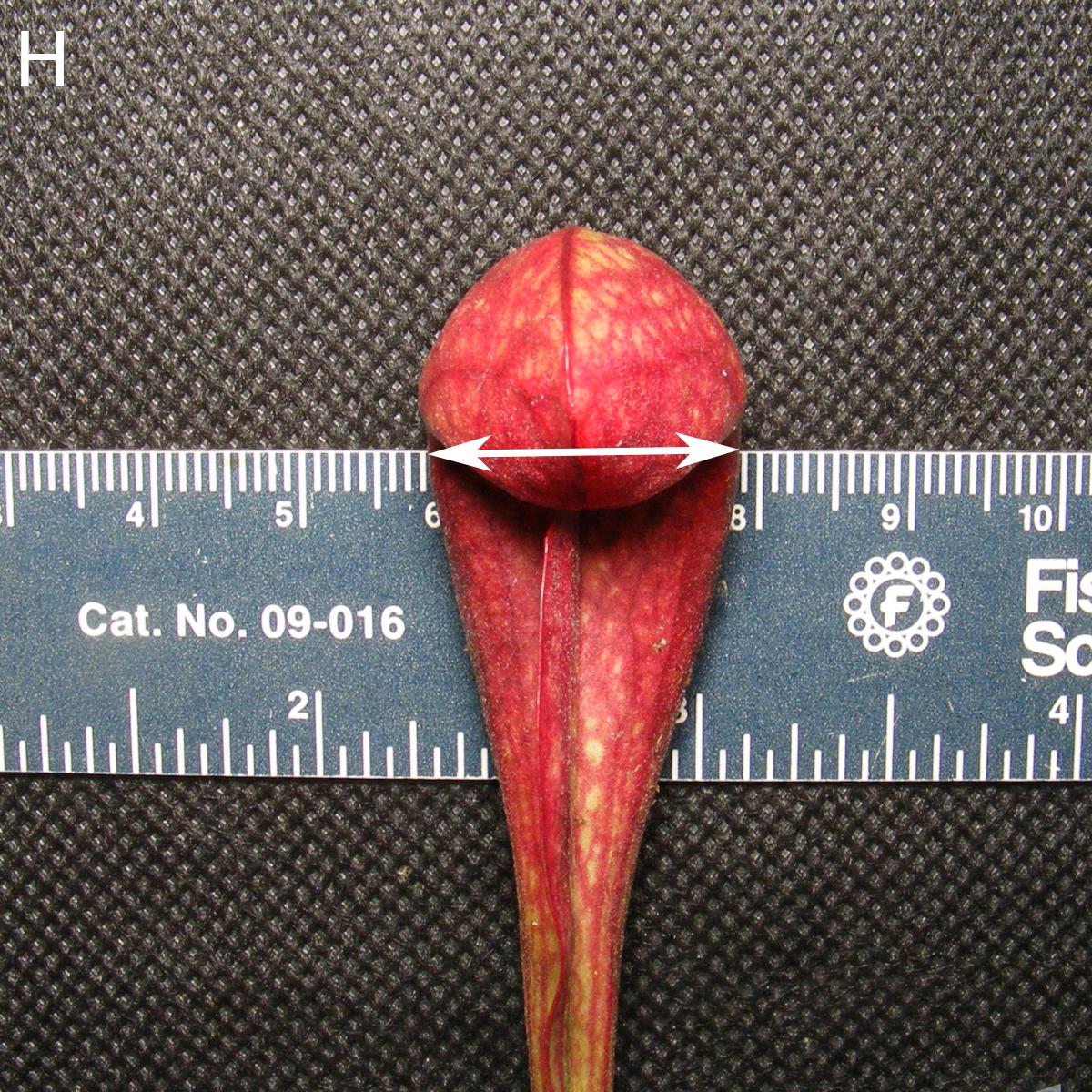

Supplement: Supplementary file 7 [file LSA-2018-00146_SdataF7.zip › Fig7H.tif]

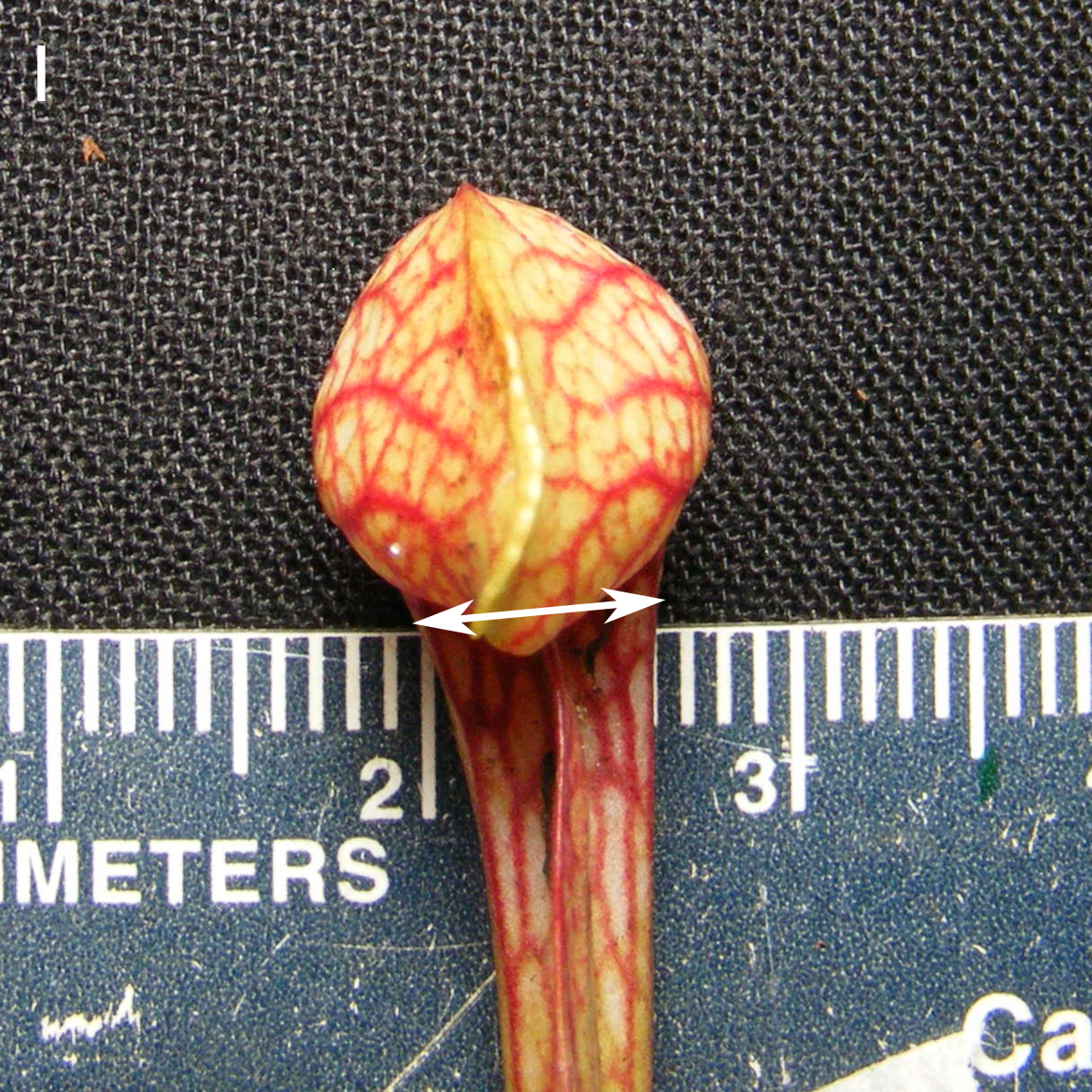

Supplement: Supplementary file 7 [file LSA-2018-00146_SdataF7.zip › Fig7I.tif]

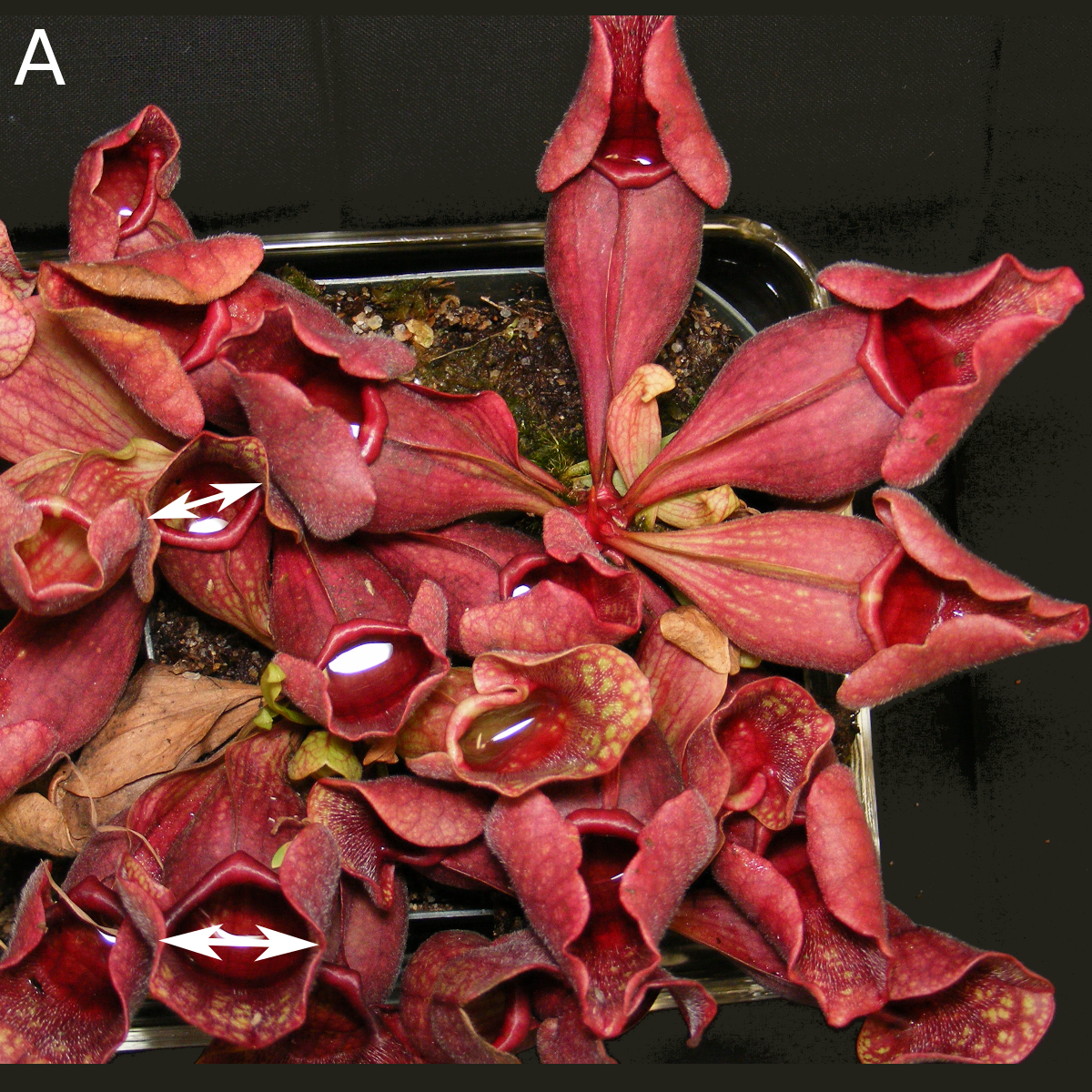

Supplement: Supplementary file 8 [file LSA-2018-00146_SdataF8.zip › Fig8A.tif]

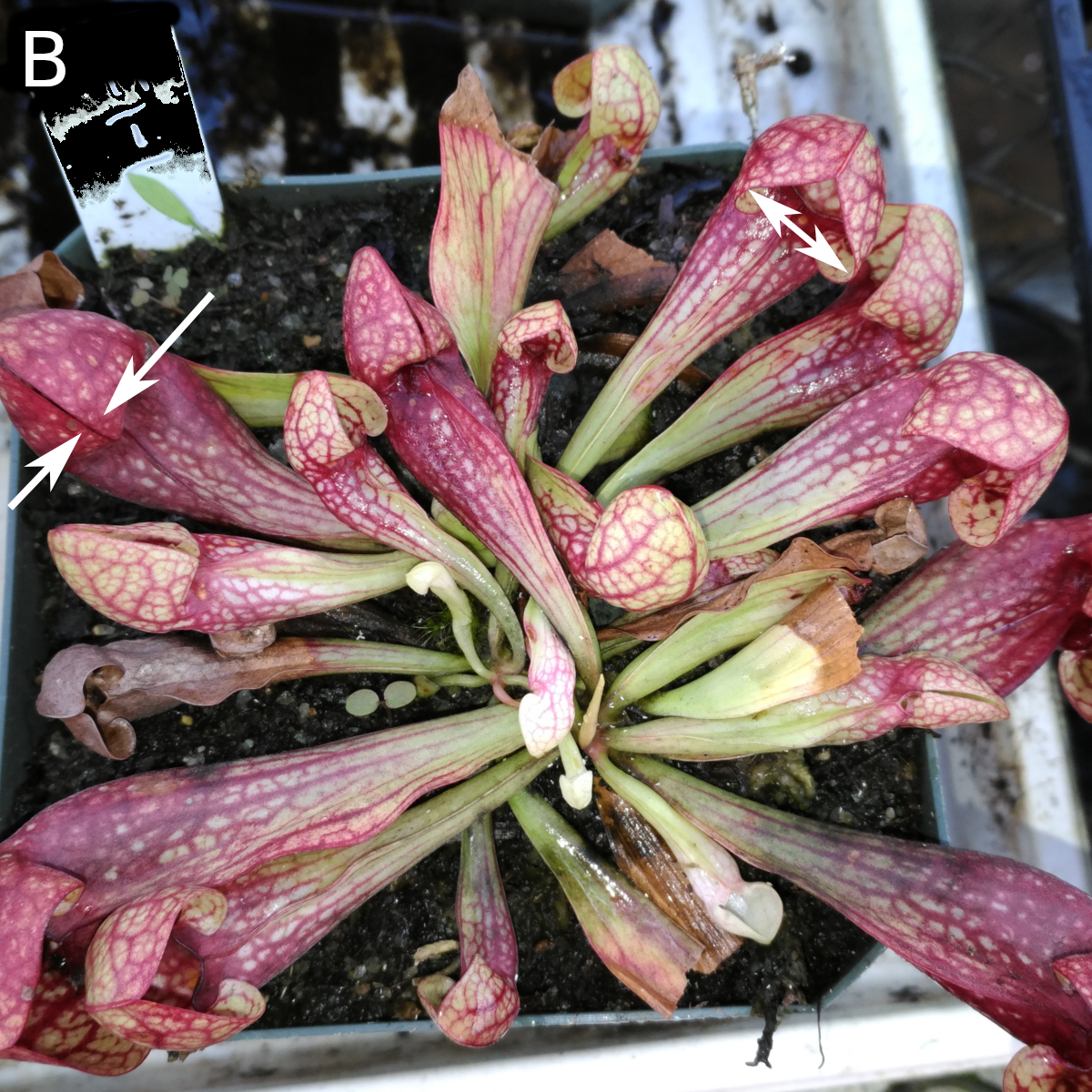

Supplement: Supplementary file 8 [file LSA-2018-00146_SdataF8.zip › Fig8B.tif]

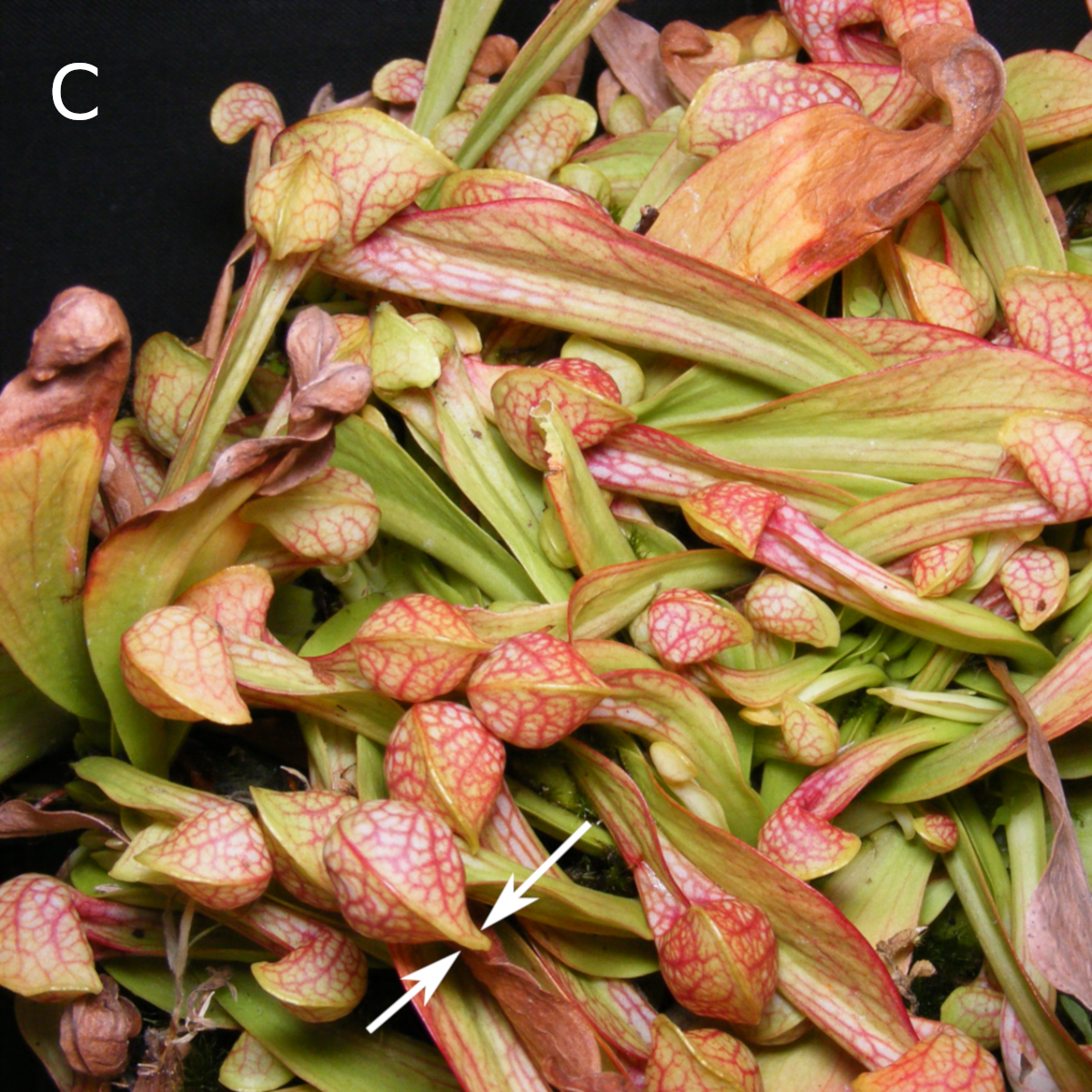

Supplement: Supplementary file 8 [file LSA-2018-00146_SdataF8.zip › Fig8C.tif]

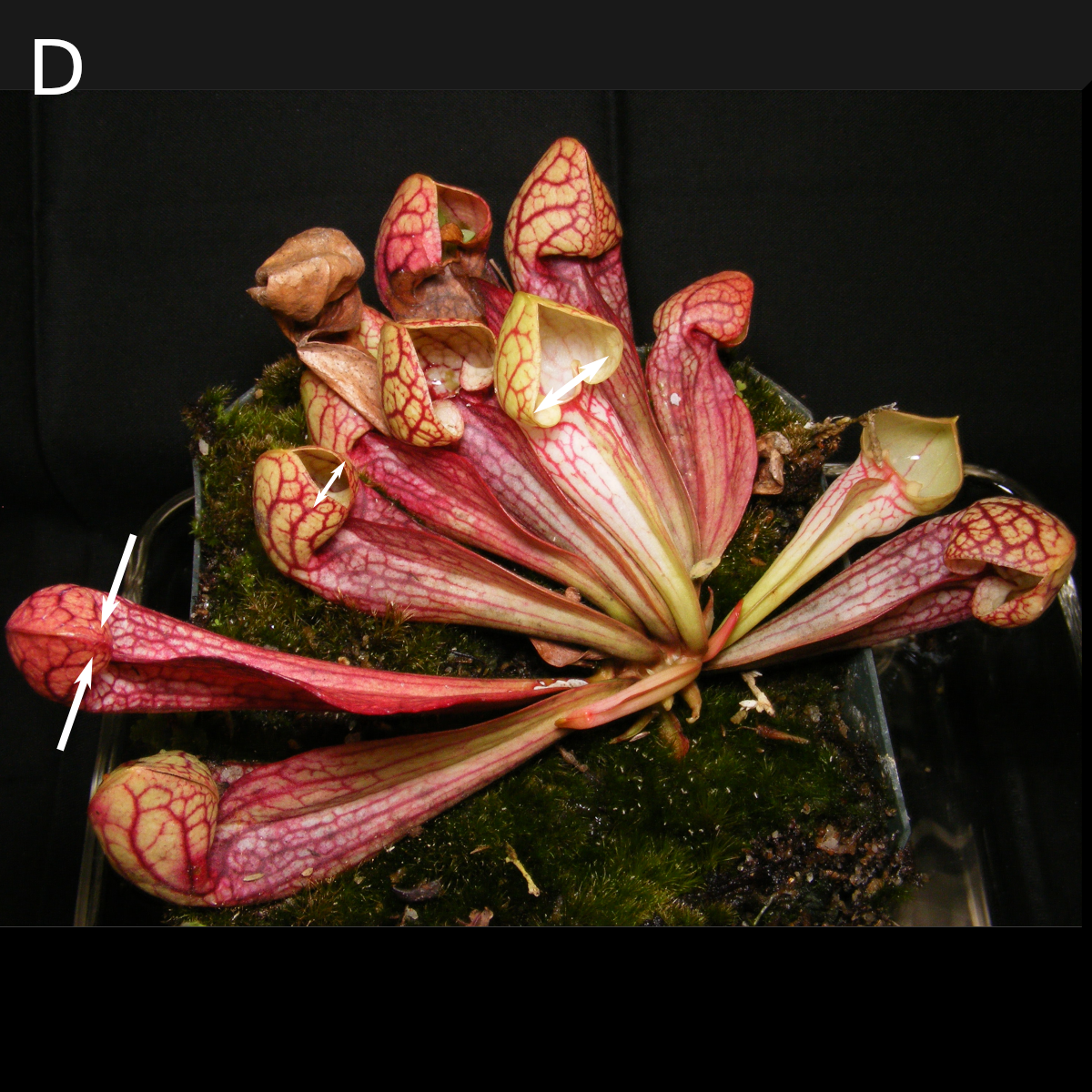

Supplement: Supplementary file 8 [file LSA-2018-00146_SdataF8.zip › Fig8D.tif]

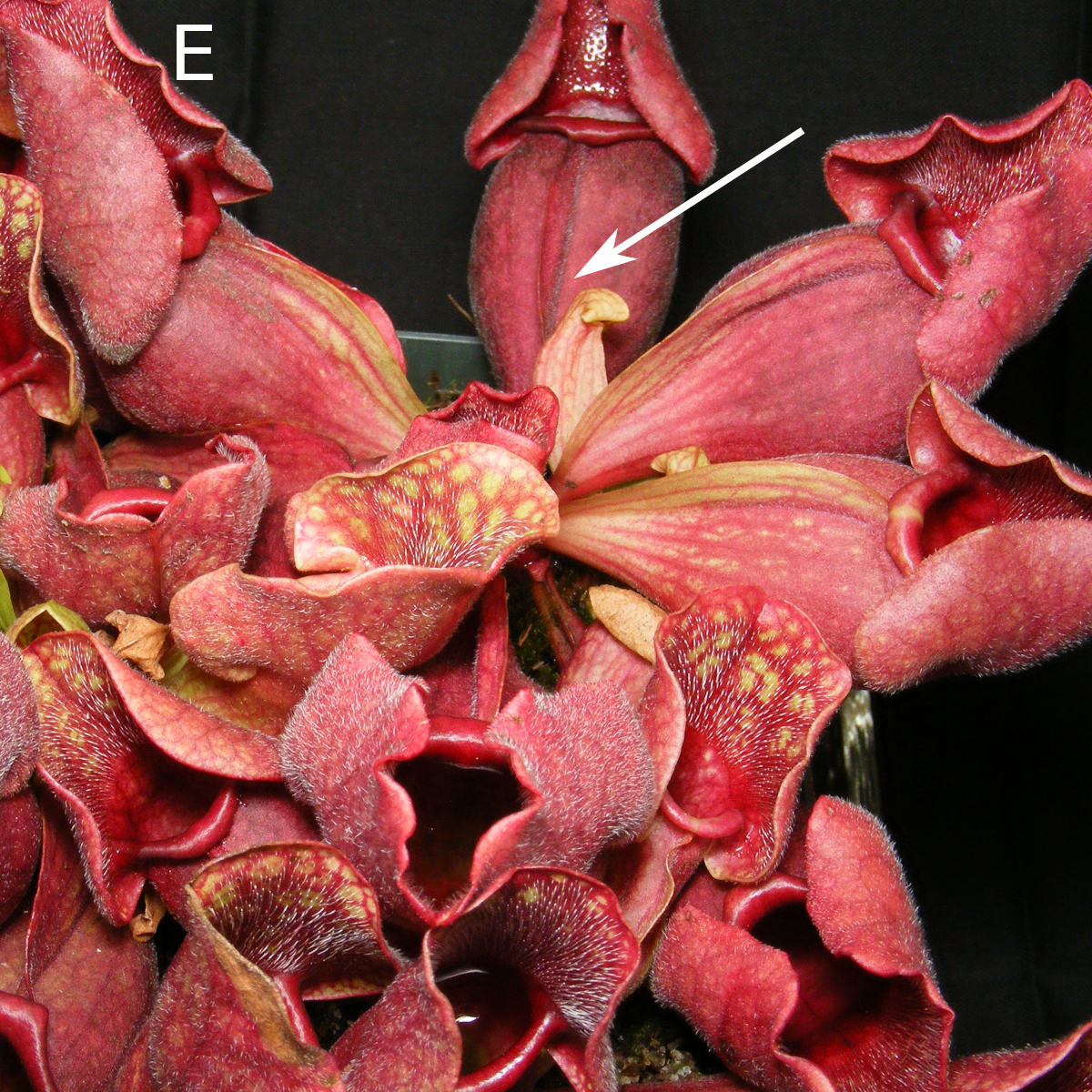

Supplement: Supplementary file 8 [file LSA-2018-00146_SdataF8.zip › Fig8E.tif]

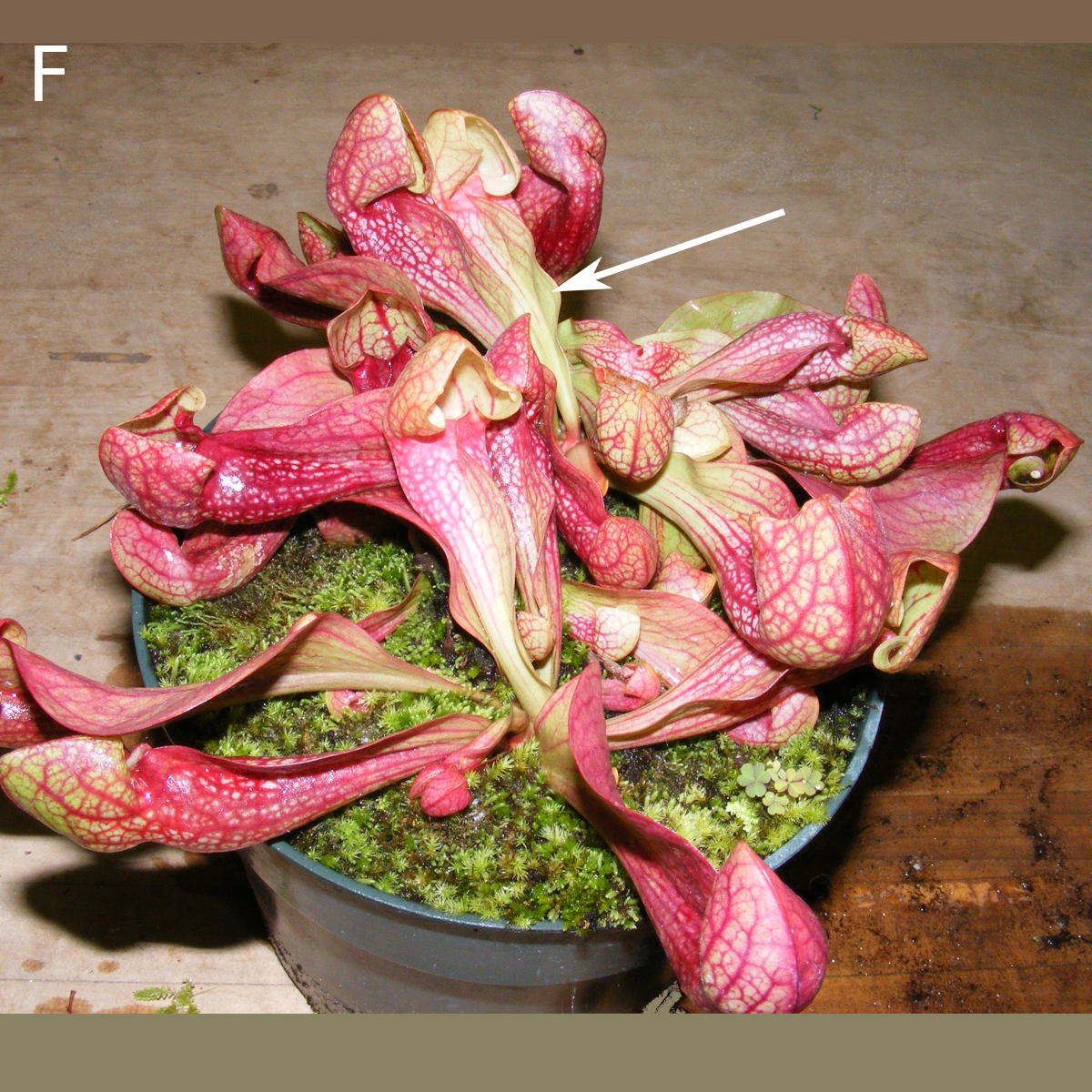

Supplement: Supplementary file 8 [file LSA-2018-00146_SdataF8.zip › Fig8F.tif]

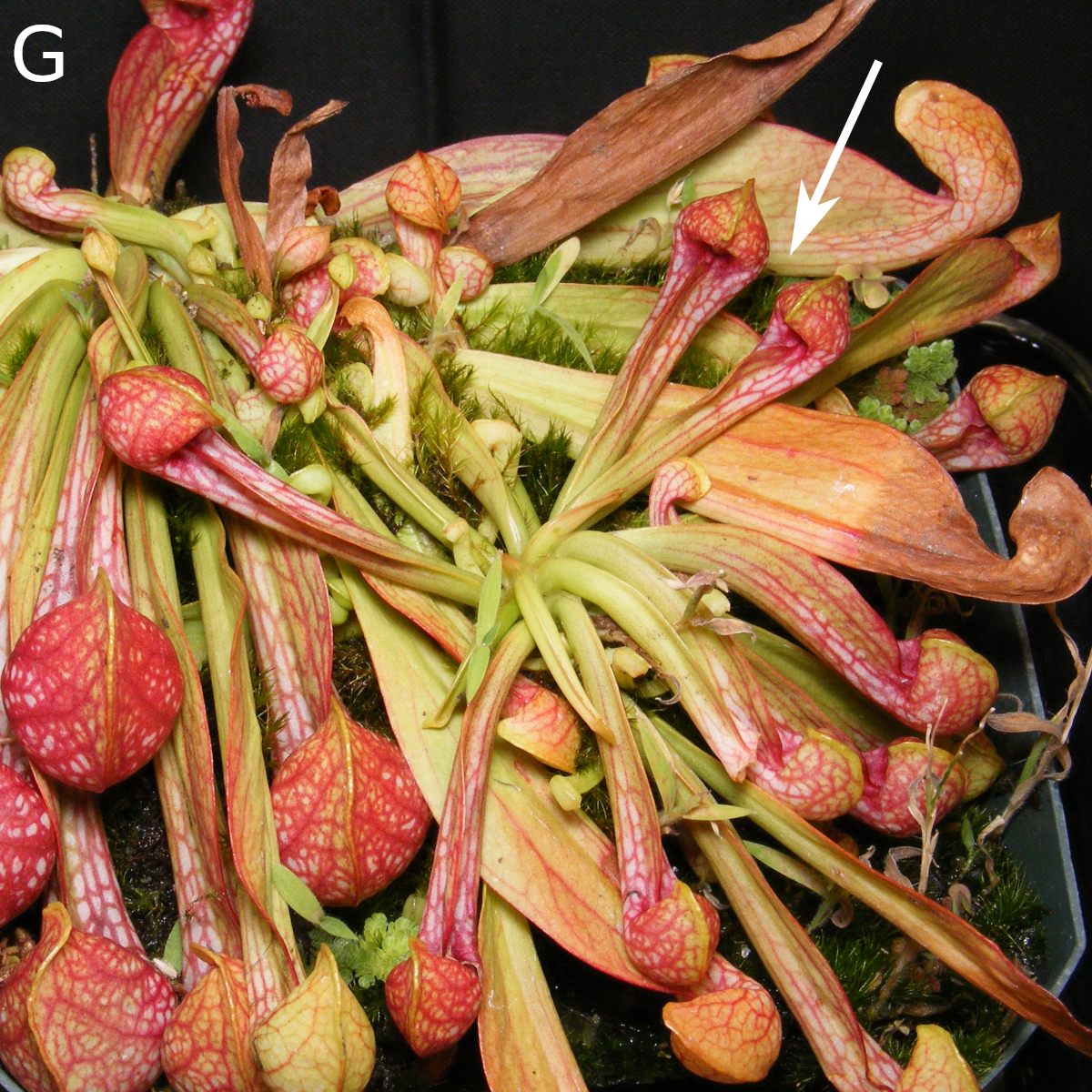

Supplement: Supplementary file 8 [file LSA-2018-00146_SdataF8.zip › Fig8G.tif]

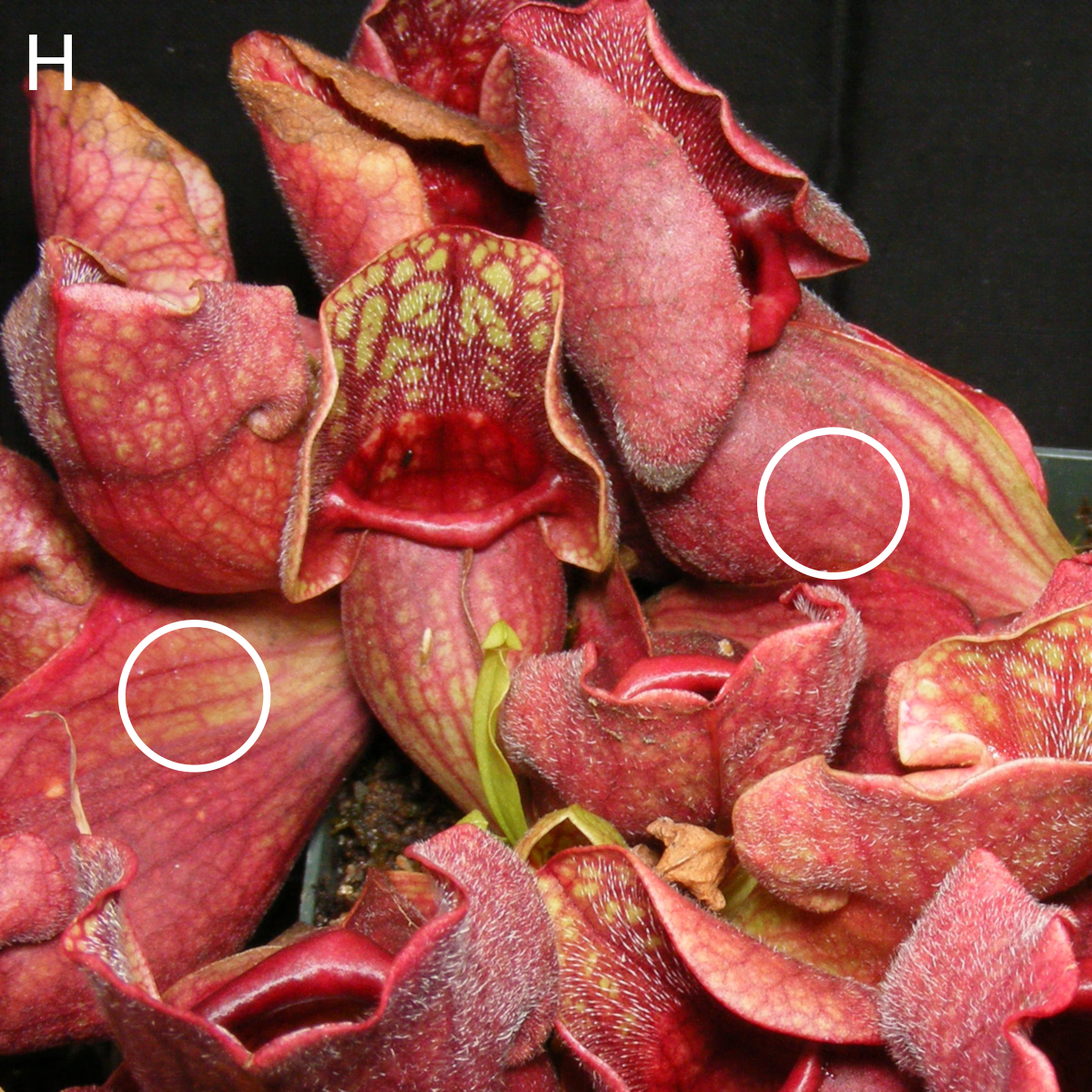

Supplement: Supplementary file 8 [file LSA-2018-00146_SdataF8.zip › Fig8H.tif]

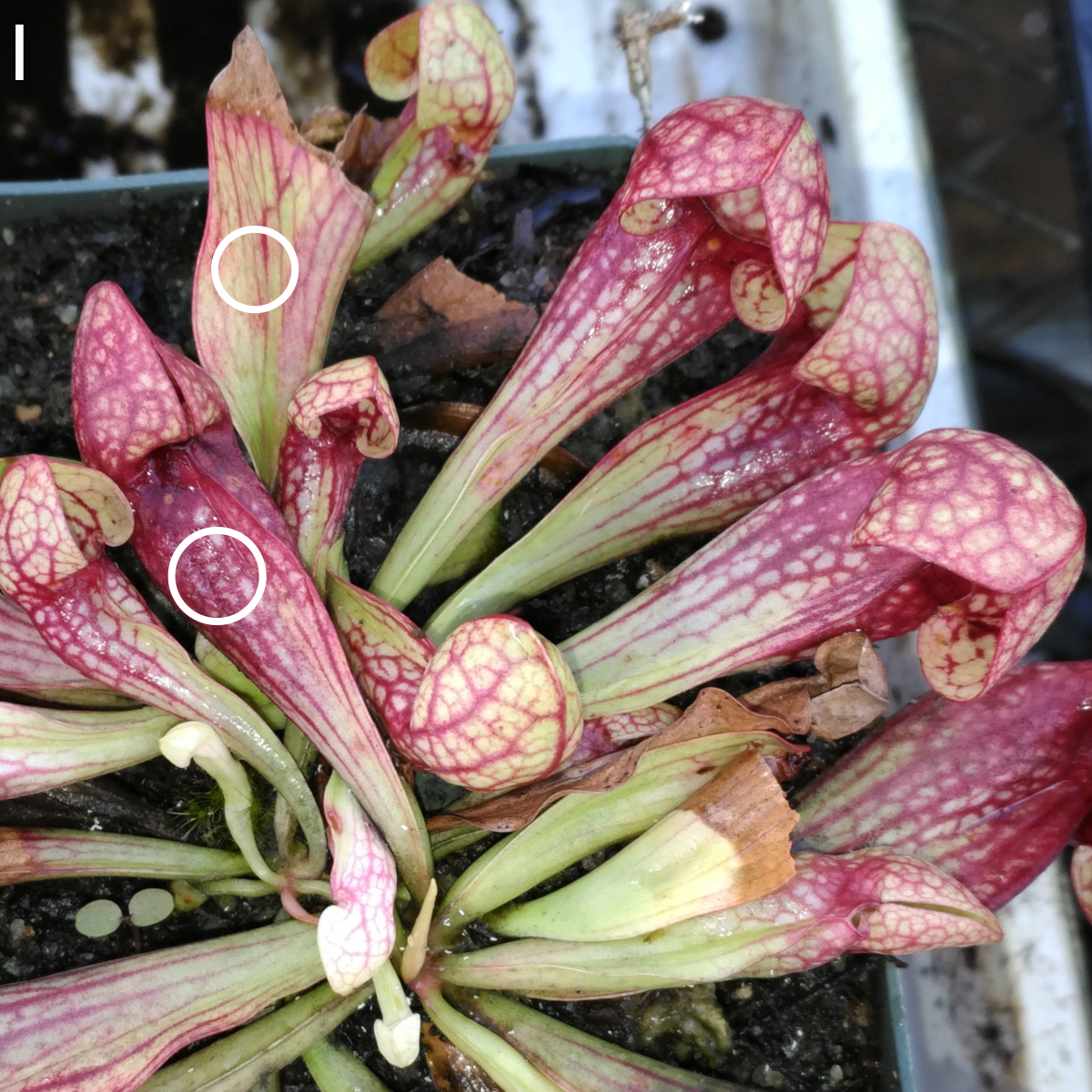

Supplement: Supplementary file 8 [file LSA-2018-00146_SdataF8.zip › Fig8I.tif]

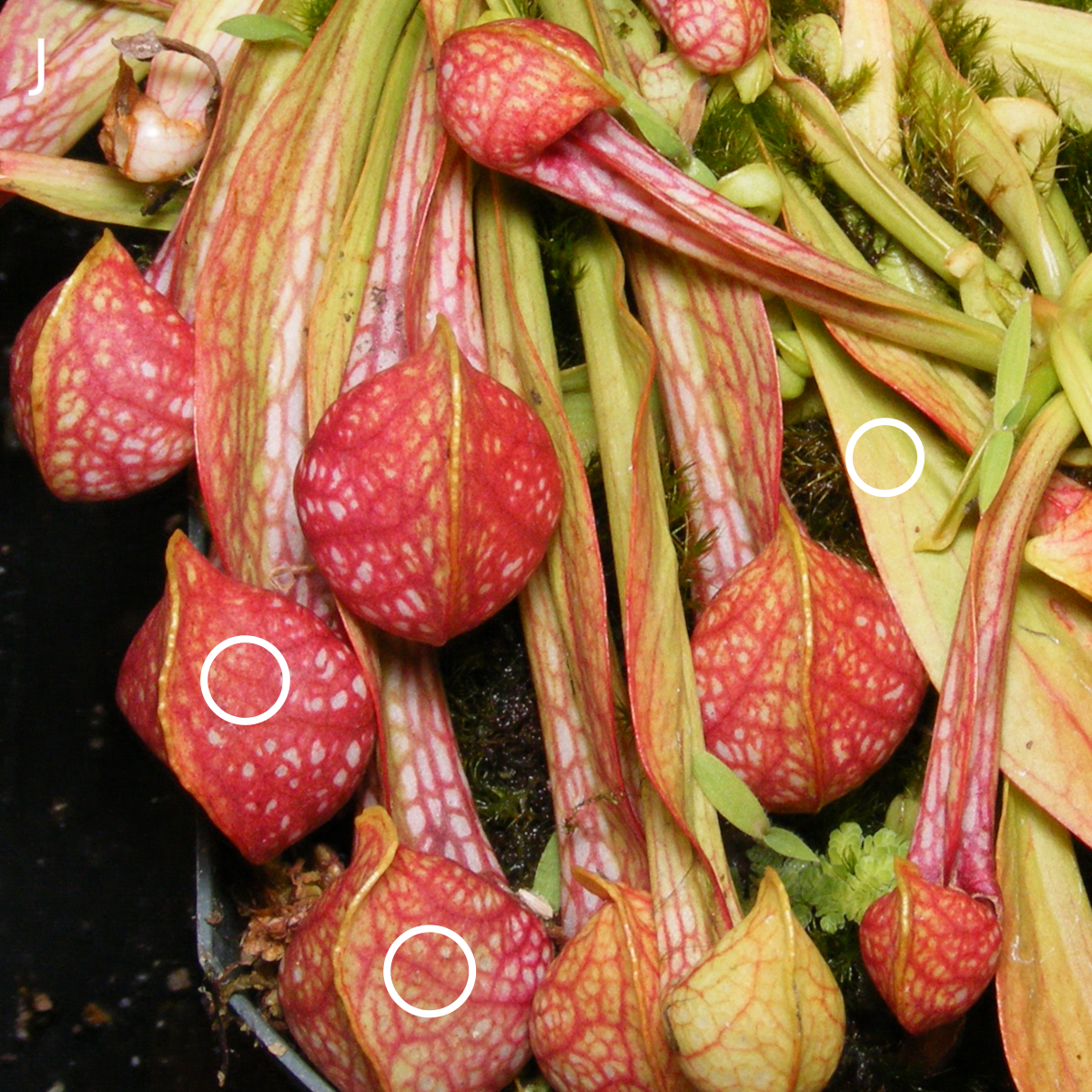

Supplement: Supplementary file 8 [file LSA-2018-00146_SdataF8.zip › Fig8J.tif]

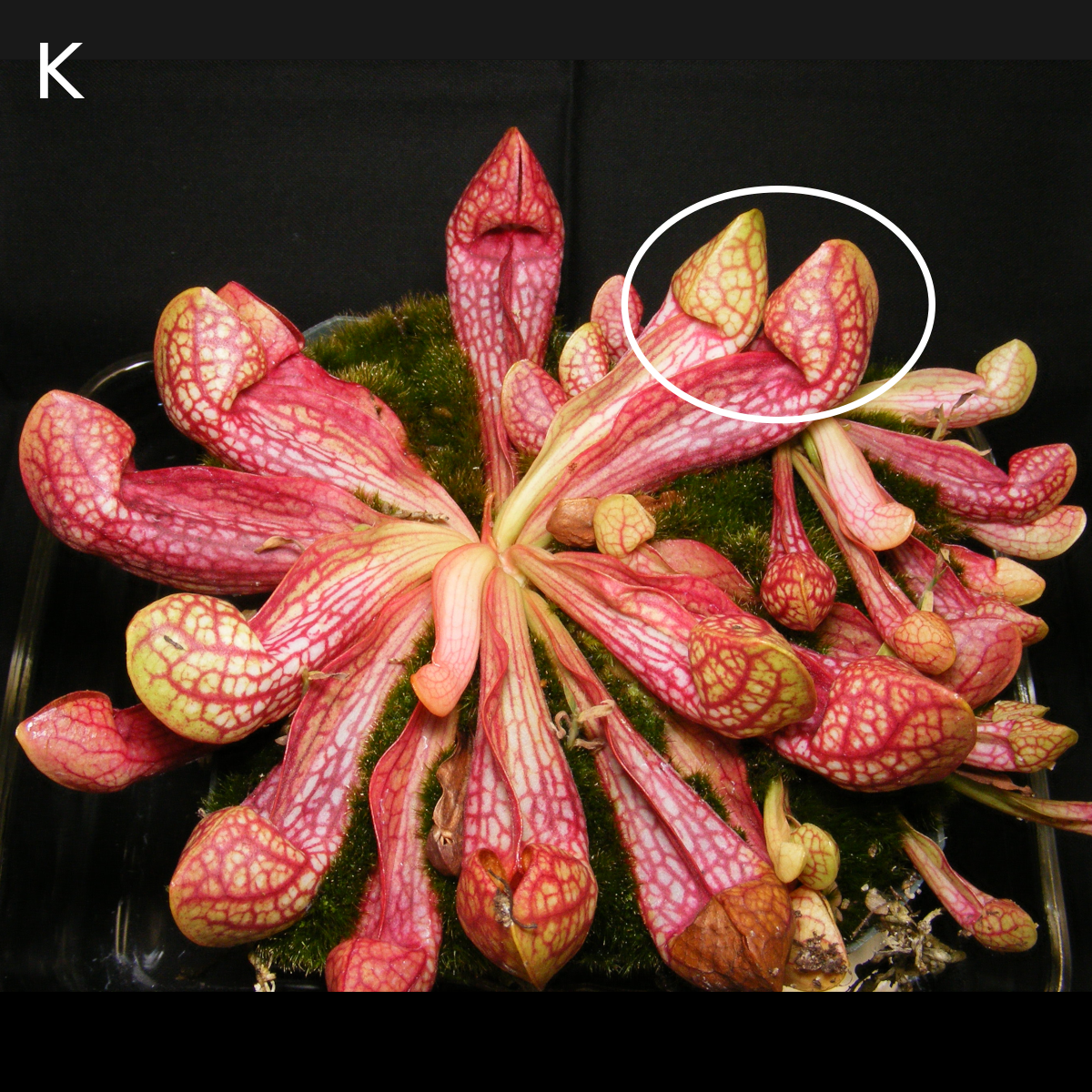

Supplement: Supplementary file 8 [file LSA-2018-00146_SdataF8.zip › Fig8K.tif]

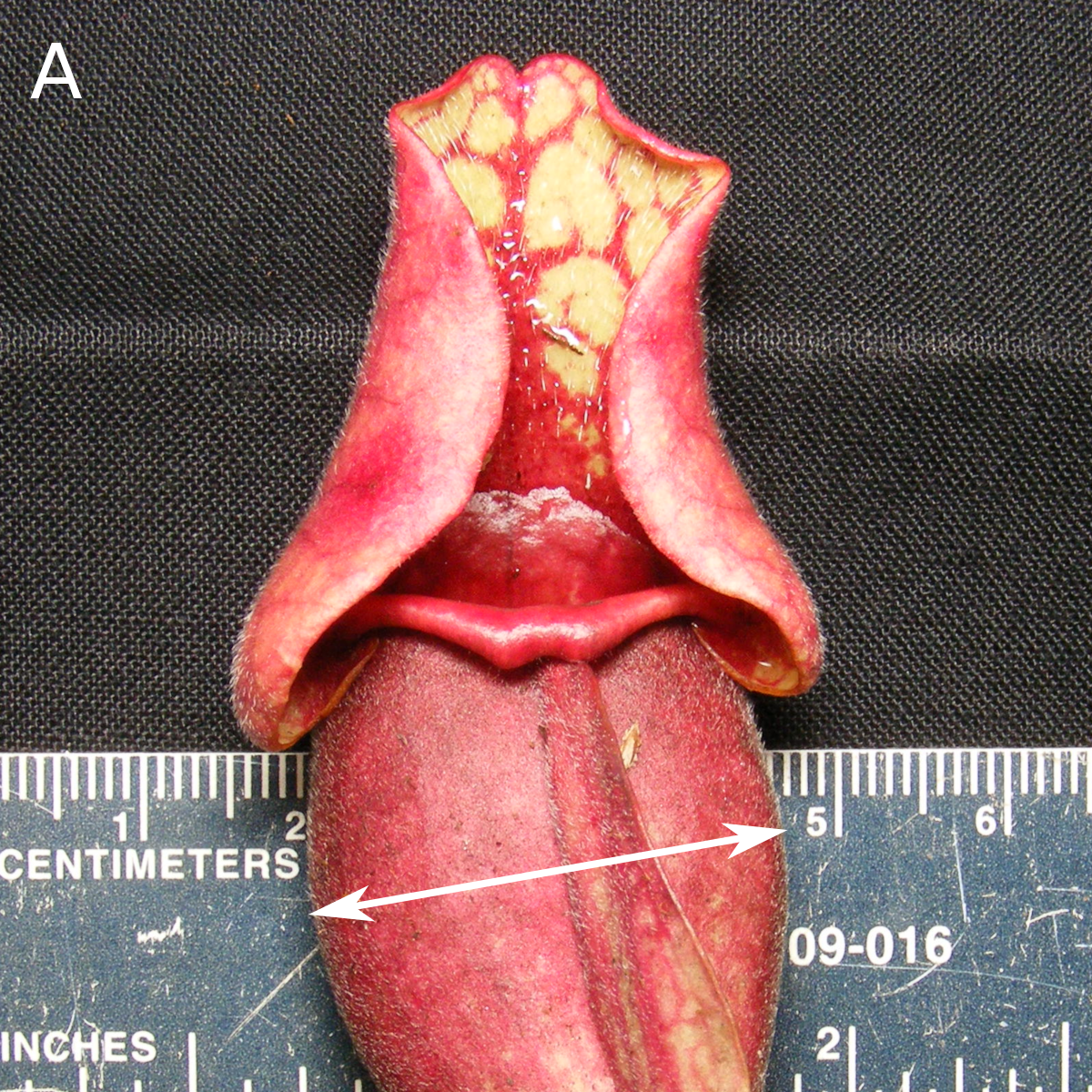

Supplement: Supplementary file 9 [file LSA-2018-00146_SdataF9.zip › Fig9A.tif]

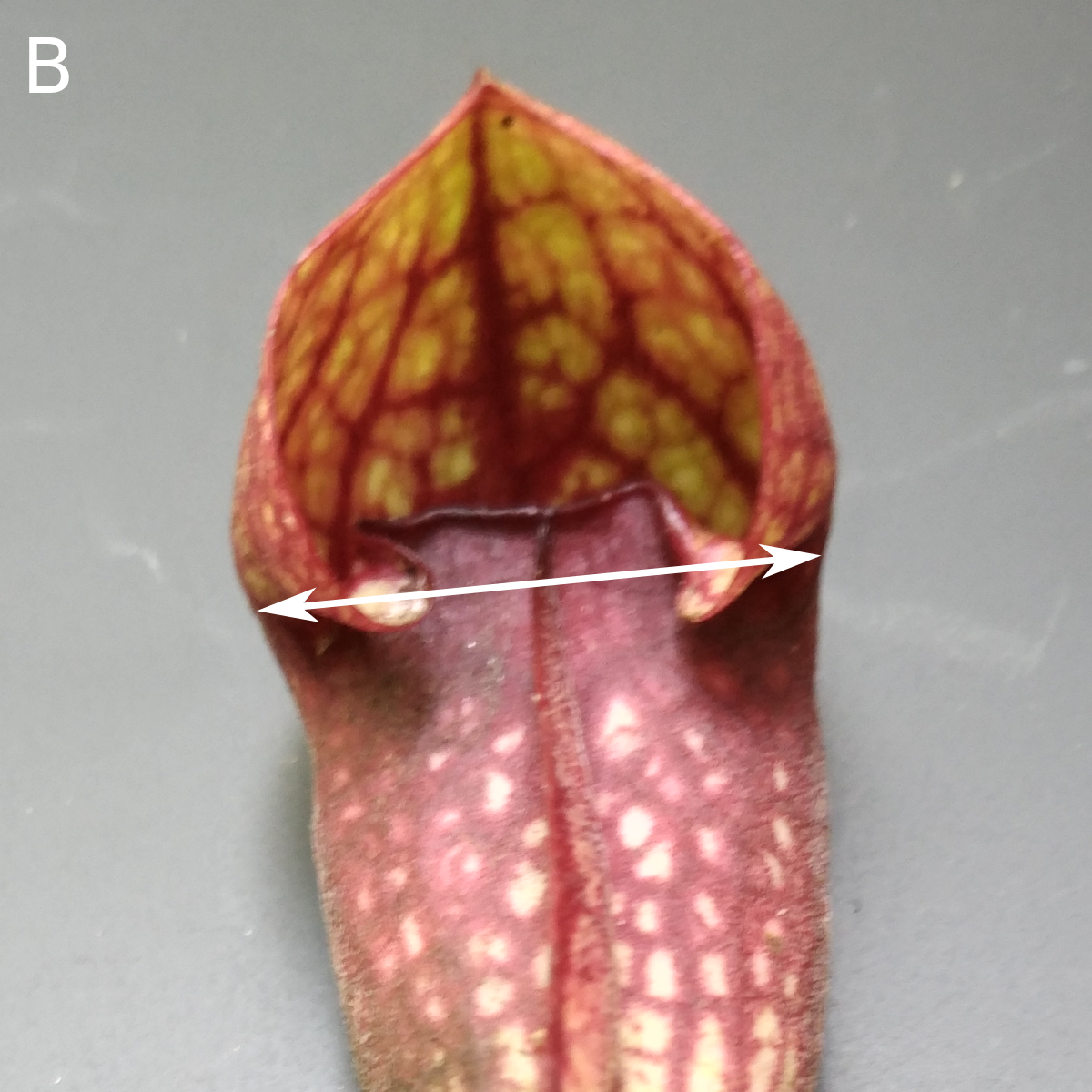

Supplement: Supplementary file 9 [file LSA-2018-00146_SdataF9.zip › Fig9B.tif]

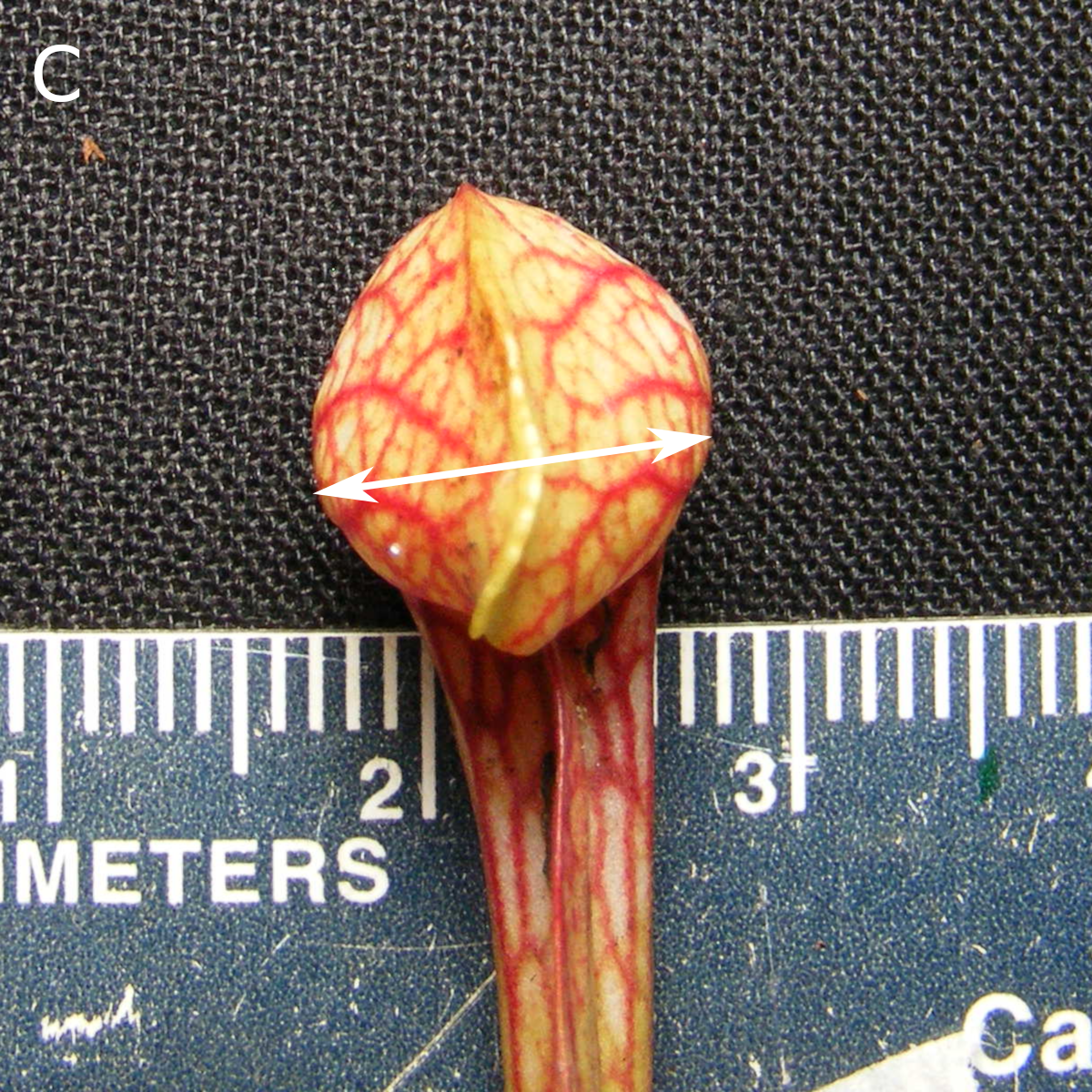

Supplement: Supplementary file 9 [file LSA-2018-00146_SdataF9.zip › Fig9C.tif]

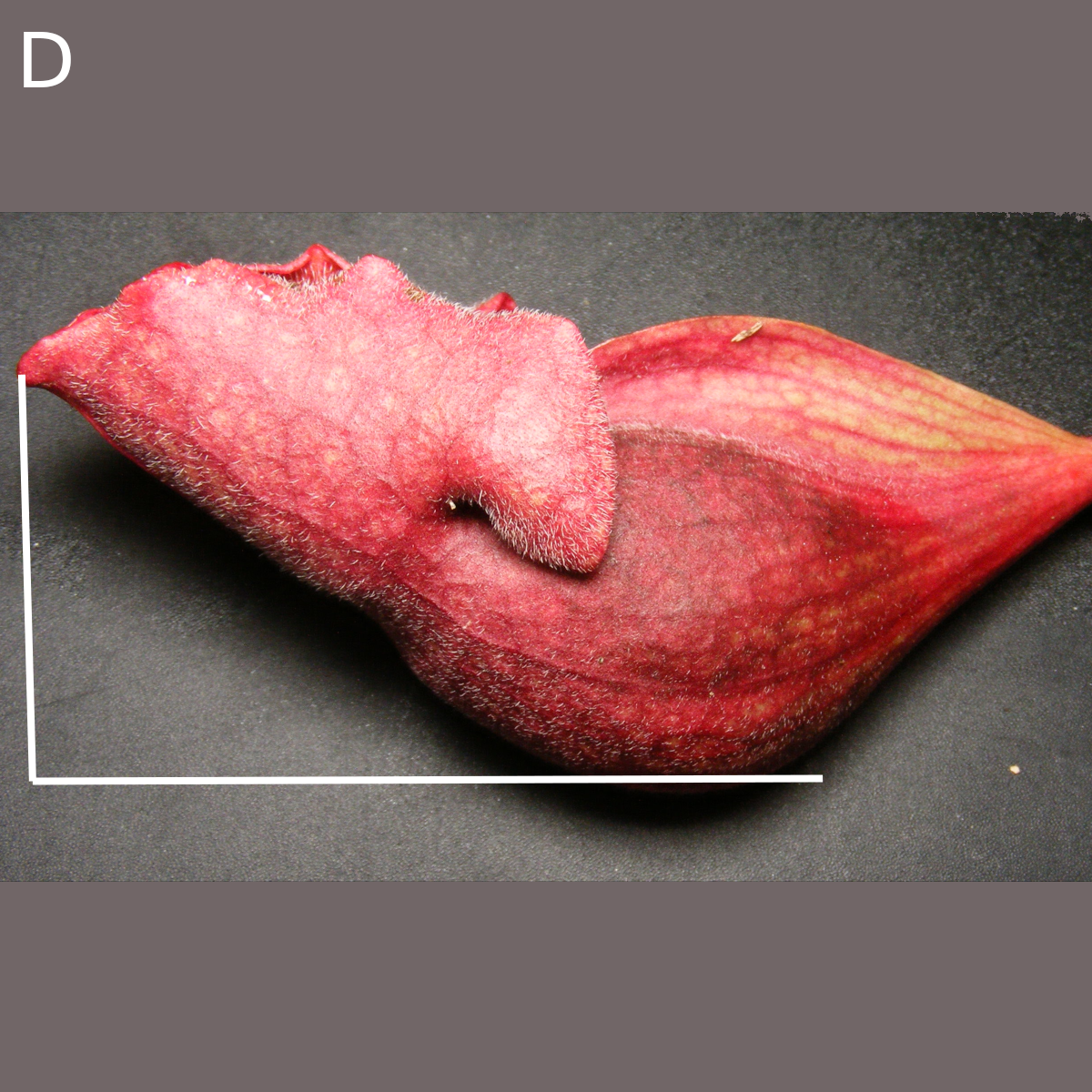

Supplement: Supplementary file 9 [file LSA-2018-00146_SdataF9.zip › Fig9D.tif]

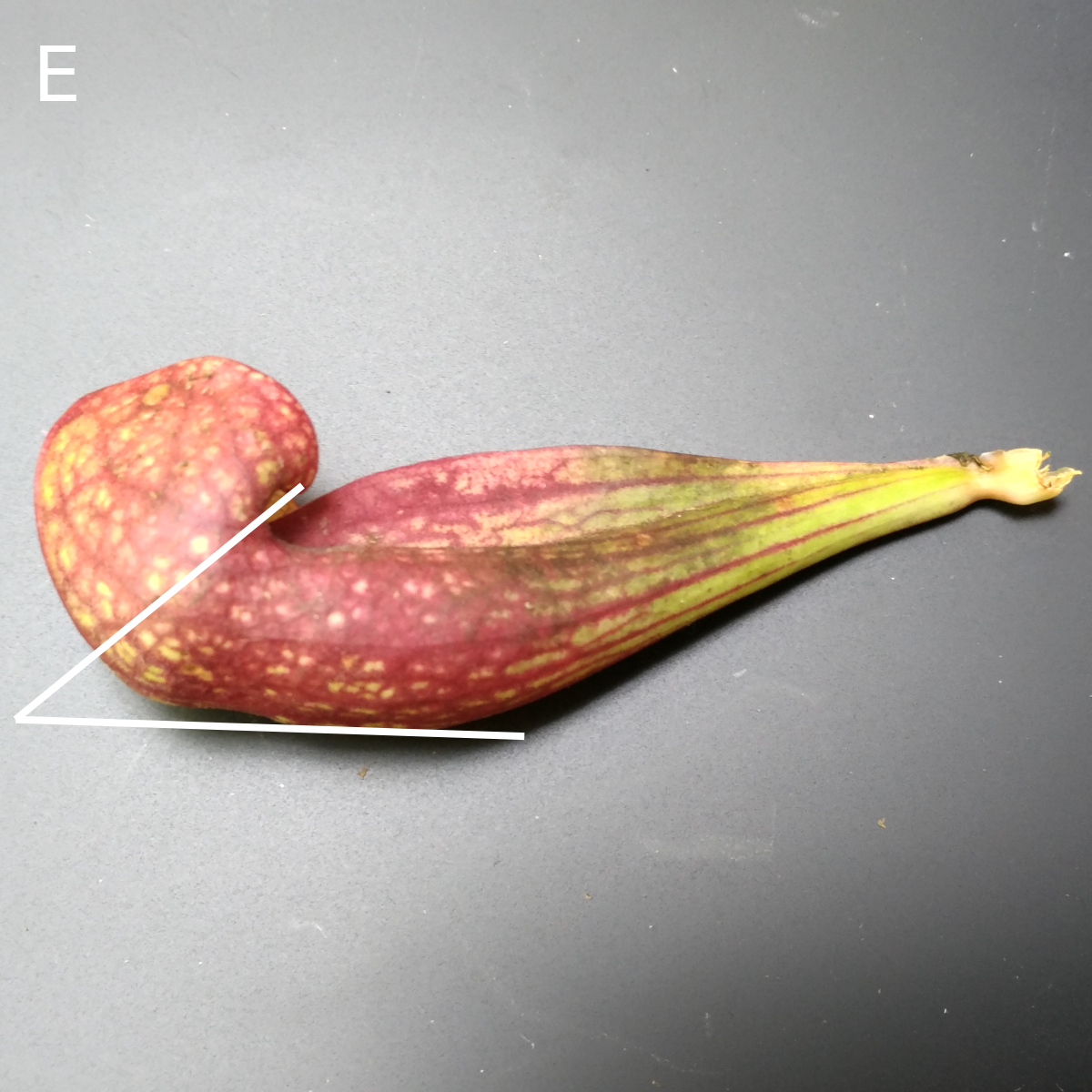

Supplement: Supplementary file 9 [file LSA-2018-00146_SdataF9.zip › Fig9E.tif]

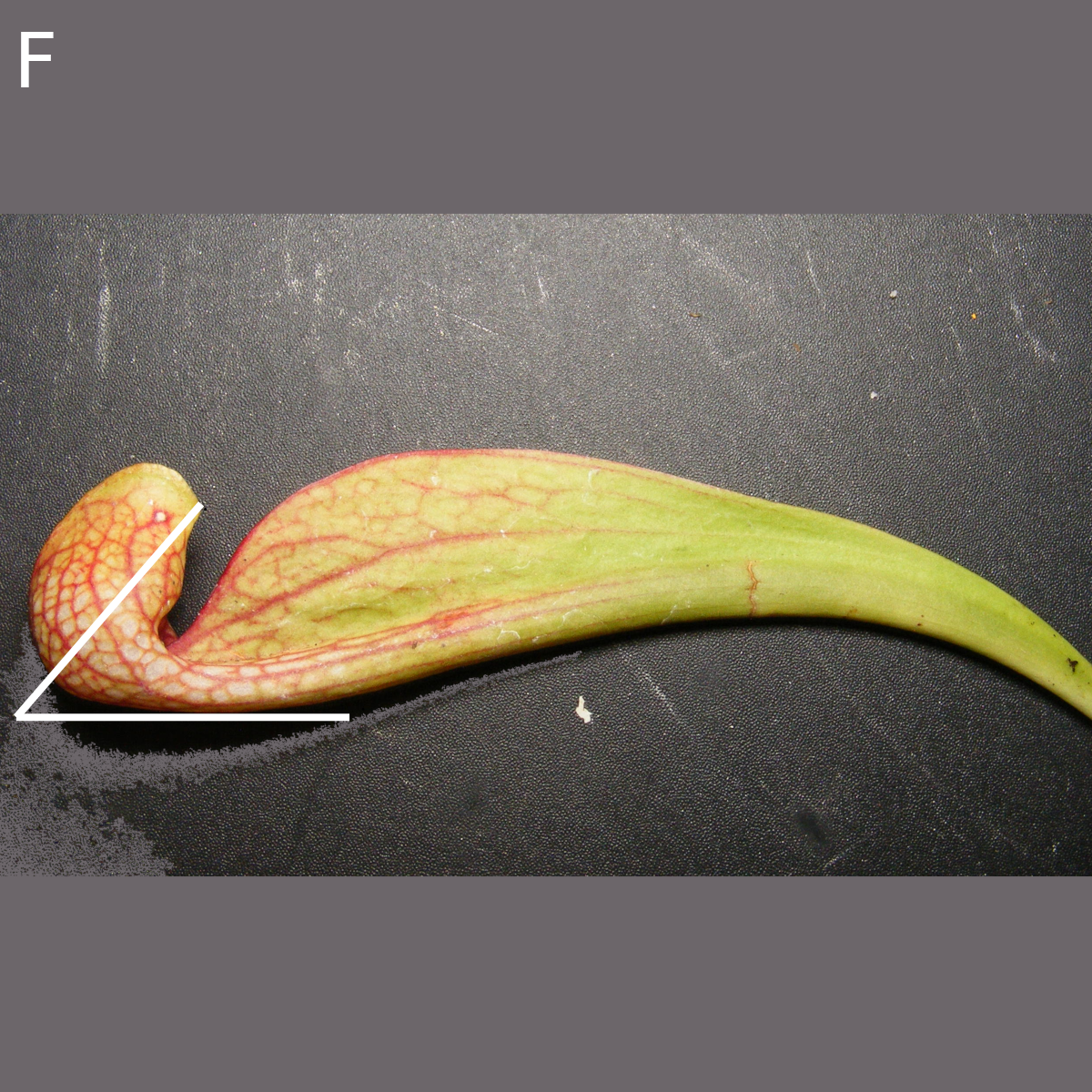

Supplement: Supplementary file 9 [file LSA-2018-00146_SdataF9.zip › Fig9F.tif]

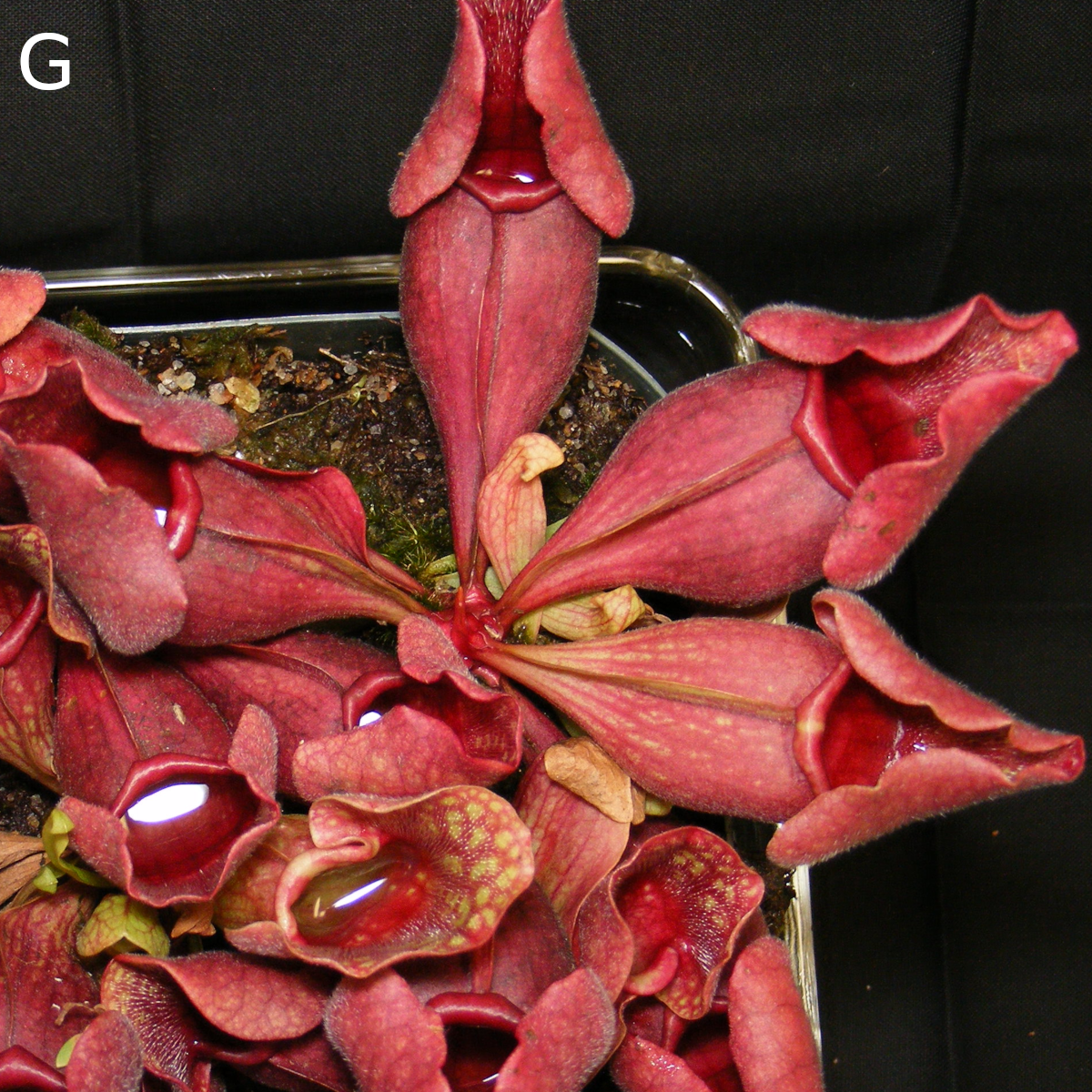

Supplement: Supplementary file 9 [file LSA-2018-00146_SdataF9.zip › Fig9G.tif]

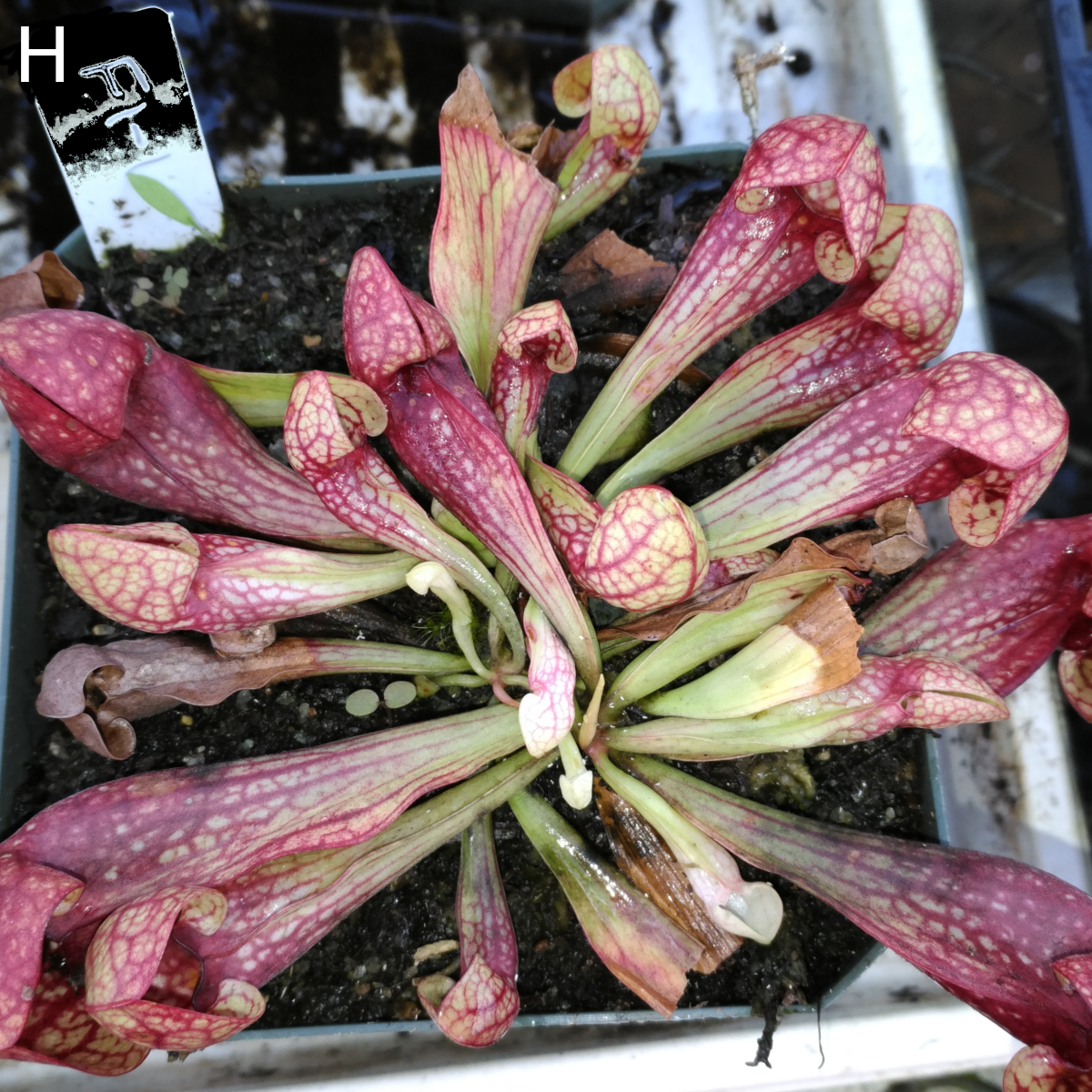

Supplement: Supplementary file 9 [file LSA-2018-00146_SdataF9.zip › Fig9H.tif]

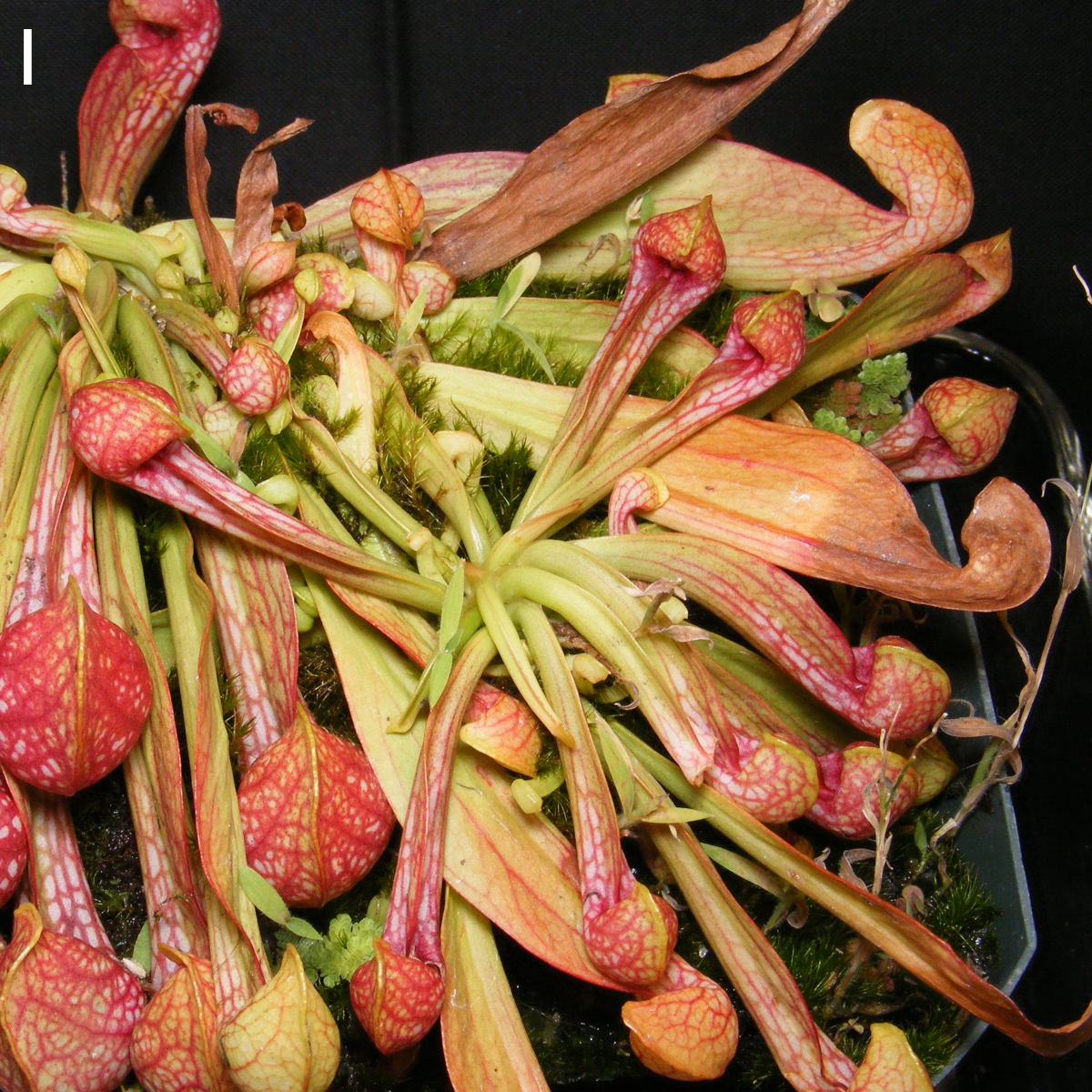

Supplement: Supplementary file 9 [file LSA-2018-00146_SdataF9.zip › Fig9I.tif]

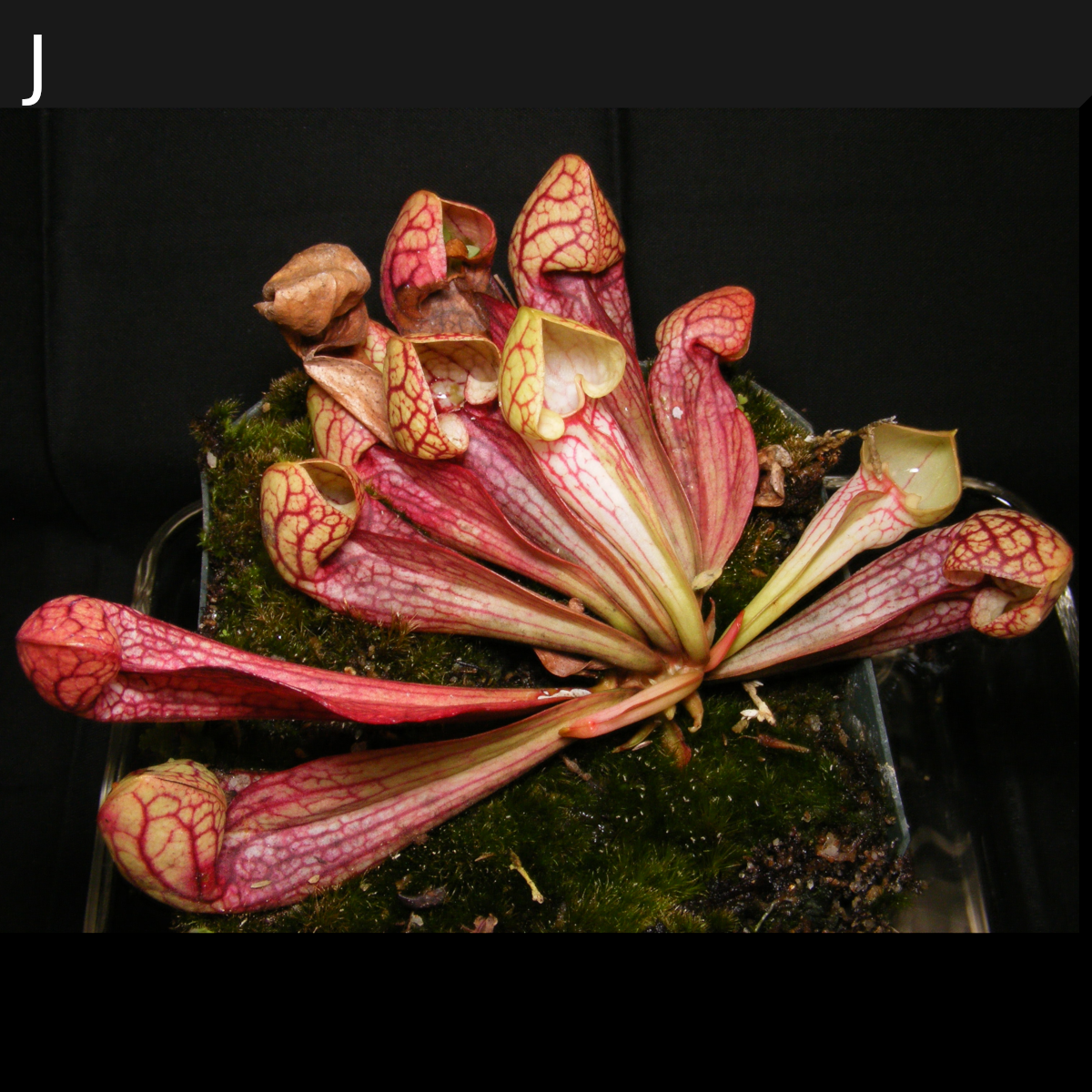

Supplement: Supplementary file 9 [file LSA-2018-00146_SdataF9.zip › Fig9J.tif]

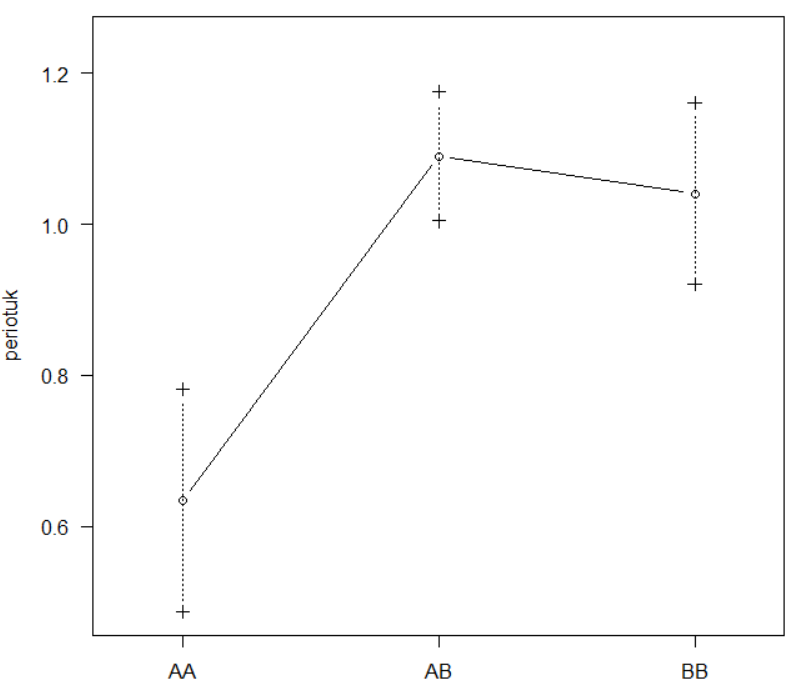

Supplement: Supplementary file 10 [file LSA-2018-00146_SdataF10.zip › FigS1A.tif]

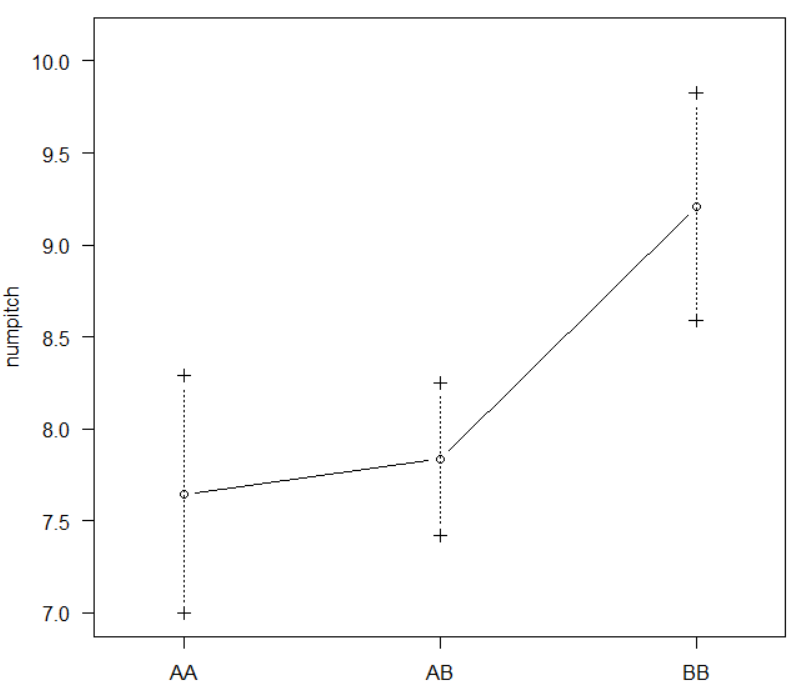

Supplement: Supplementary file 10 [file LSA-2018-00146_SdataF10.zip › FigS1B.tif]

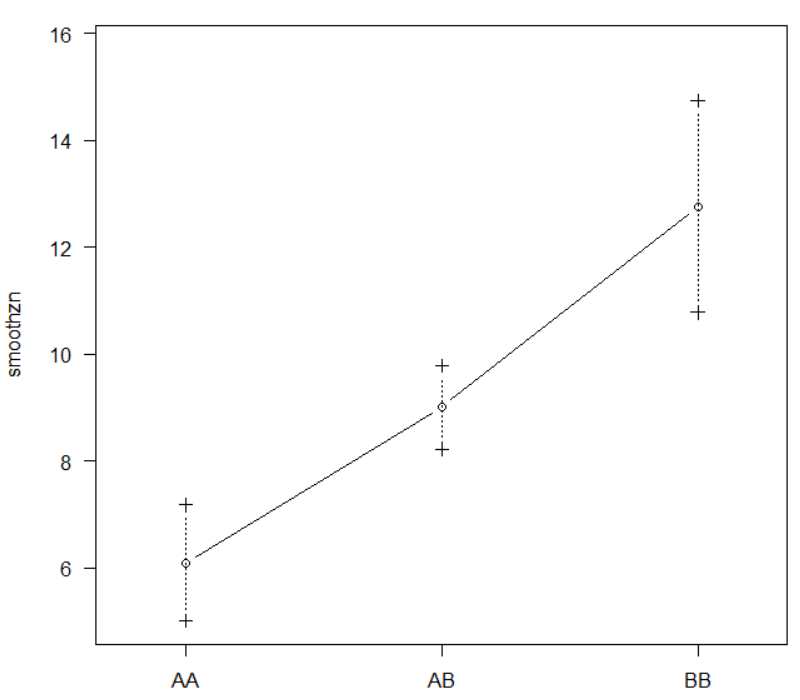

Supplement: Supplementary file 10 [file LSA-2018-00146_SdataF10.zip › FigS1C.tif]

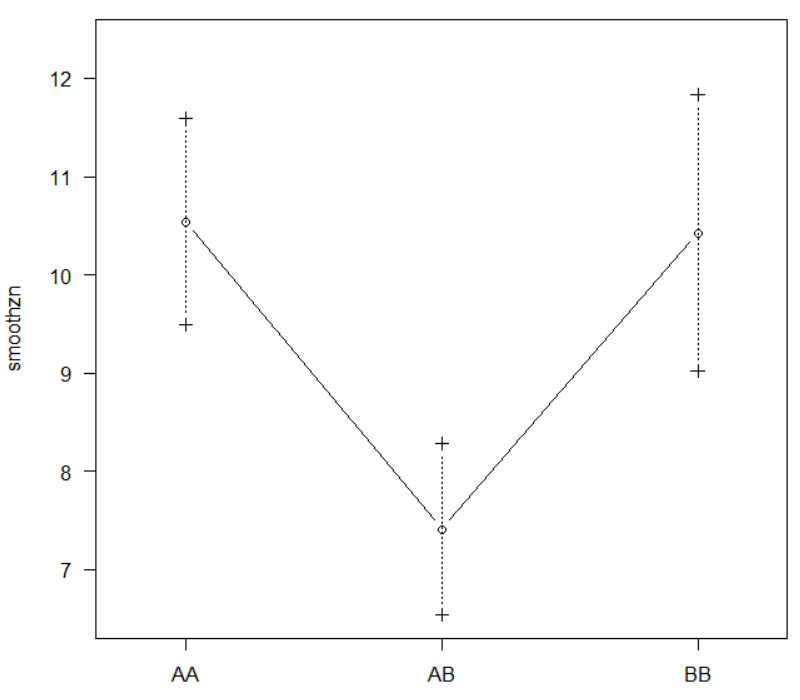

Supplement: Supplementary file 10 [file LSA-2018-00146_SdataF10.zip › FigS1D.tif]
